# GSTP1 improves CAR-T cell proliferation and cytotoxicity to combat lymphoma

**DOI:** 10.3389/fimmu.2025.1665407

**Published:** 2025-09-26

**Authors:** Guangsong Xu, Jiani Wang, Yuliang Qu, Jing Ning, Yanting Zhang, Guangxian Xu, Yunxia Shi, Ying Li, Le Guo, Xuebo Han, Hongxia Wang

**Affiliations:** ^1^ College of Laboratory Medicine, Ningxia Medical University, Yinchuan, Ningxia, China; ^2^ Hematology Department, General Hospital of Ningxia Medical University, Yinchuan, Ningxia, China; ^3^ Gastroenterology Department, General Hospital of Ningxia Medical University, Yinchuan, Ningxia, China; ^4^ Medical Technology College, Guangdong Medical University, Dongguan, Guangdong, China; ^5^ Key Laboratory of Clinical Pathogenic Microbiology, General Hospital of Ningxia Medical University, Yinchuan, Ningxia, China

**Keywords:** CAR-T cell exhaustion, BLIMP1, GSTP1, oxidative stress, reactive oxygen species

## Abstract

**Introduction:**

The exhaustion of chimeric antigen receptor T cells (CAR-T) hampers the efficacy of CAR-T cell therapy. Persistent antigen stimulation in T cells results in a surge of intracellular reactive oxygen species (ROS). ROS, as mitochondrial metabolites, alter the integrity of the mitochondrial membrane and promote T-cell exhaustion. Glutathione *S*-transferase Pi-1 (GSTP1), a member of the glutathione *S*-transferase family, is an important enzyme in the intracellular clearance of ROS. Overexpression of GSTP1 may enhance the antitumor capability of CAR-T cells.

**Methods:**

The correlations between GSTP1 and genes related to T-cell exhaustion were analyzed using the TIMER database. Peripheral blood mononuclear cells (PBMCs) were collected from patients with hematologic malignancies (*n* = 61) and healthy donors (*n* = 45) to measure GSTP1, B-lymphocyte maturation protein 1 (BLIMP1), and programmed cell death protein 1 (PD-1) expression by qRT-PCR. A T-cell exhaustion model was established to assess GSTP1 expression by Western blotting. The dual-luciferase assay and ChIP-qPCR were used to determine whether the transcription factor BLIMP1 negatively regulated the activity of the GSTP1 promoter. CD19 CAR-T, GSTP1 overexpressing CAR-T (GSTP1 CAR-T), and GSTP1-knockdown CAR-T (shGSTP1 CAR-T) cells were generated to evaluate their antitumor capacity.

**Results:**

GSTP1 expression was downregulated when BLIMP1 and PD-1 were upregulated in PBMCs of cancer patients and in the *in vitro* T-cell exhaustion model. Meanwhile, ROS levels in the T-cell exhaustion model increased. Mechanistically, the BLIMP1 transcription factor negatively regulated the activity of the GSTP1 promoter. Based on these findings, we engineered GSTP1 CAR-T cells, which exhibited improved functionality. GSTP1 CAR-T cells increased the TEMRA population, enhanced proliferation and cytotoxicity, elevated antioxidant capacity, increased IL-2 and IFN-γ secretion, reduced the expression of immune checkpoints, and decreased apoptosis. *In vivo*, the residual levels of GSTP1 CAR-T cells were higher than those of Cluster of Differentiation 19 (CD19) CAR-T cells and shGSTP1 CAR-T cells, indicating that GSTP1 CAR-T cells exhibited a strong antitumor capacity.

**Conclusion:**

BLIMP1 directly suppressed GSTP1 transcription, whereas GSTP1 overexpression enhanced the antitumor capacity of CAR-T cells and maintained redox homeostasis, providing a novel therapeutic strategy to improve CAR-T cell immunotherapy.

## Introduction

Chimeric antigen receptor T-cell (CAR-T) immunotherapy has revolutionized the treatment of hematological malignancies, offering durable complete remissions and high objective response rates for heavily pretreated refractory patients (1, 2). However, high relapse rates still occur in patients with hematological tumors following CAR-T therapy. CAR-T cell exhaustion, a major hurdle in cancer immunotherapy, is the main factor reducing the cure rate and hampering antitumor efficacy (3). Various strategies to improve CAR-T exhaustion have been attempted, including optimizing CAR-T cell structure and culture systems (4), targeting the tumor microenvironment, and modulating epigenetic and transcriptomic regulation (5).

Studies have indicated that sustained antigen stimulation increases intracellular reactive oxygen species (ROS) levels in T cells, damaging adenosine triphosphate (ATP)-coupled oxidative phosphorylation (6). This bioenergetic impairment limits nucleotide triphosphate synthesis, blocking proliferation and affecting T-cell self-renewal (6). Continuous antigen stimulation also disrupts T-cell function, as evidenced by elevated expression of TIM-3 and programmed cell death protein 1 (PD-1) (7). ROS include superoxide anions, hydrogen peroxide, hydroxyl radicals, ozone, and singlet oxygen (8). Physiologically, ROS are derived from mitochondrial respiration and Nicotinamide Adenine Dinucleotide Phosphate (NADPH) oxidases, and they act as signaling molecules in the activation, proliferation, and development of T cells. However, under hypoxic or stress conditions, ROS production surges and damages transcription factors, proteins, lipids, and DNA (9). Glutathione *S*-transferase Pi-1 (GSTP1) helps maintain ROS redox balance, thereby preventing damage induced by oxidative stress (10).

GSTP1 promotes tumor cell proliferation (11), but its role in T cells remains poorly understood, especially when T cells are stimulated by antigens. Reduced expression of GSTP1, leading to an increase in mitochondrial ROS levels, weakens cellular function, while overexpression of GSTP1 decreases mitochondrial ROS and restores cellular function (12). To maintain redox balance, effective antioxidant mechanisms involve reduced glutathione (GSH), NADPH, and redox-regulating proteins, including electrophilic ROS (such as lipid peroxides and 4-hydroxynonenal) (13). GSH, a key endogenous antioxidant, serves as the primary ROS scavenging pathway in cells. GSTP1 catalyzes GSH conjugation with electrophilic ROS to form nontoxic GSH conjugates, which are subsequently exported out of the cell, thereby mitigating ROS-induced oxidative damage to cellular components (10).

Our study demonstrated that GSTP1 was downregulated in the peripheral blood mononuclear cells (PBMCs) of cancer patients and in the T-cell exhaustion model. After overexpressing GSTP1 in CAR-T cells, multiple functions improved, including an increase in the population of terminal effector memory T cells, reduced expression of immune checkpoints, decreased apoptosis, and enhanced proliferative capacity. Additionally, GSTP1-overexpressing CAR-T cells increased cytotoxicity, elevated secretion of interleukin (IL)-2 and IFN-γ, and enhanced antioxidant capacity. These findings were further validated in a mouse lymphoma model, where GSTP1-overexpressing CAR-T (GSTP1 CAR-T) cells significantly improved the survival rate of mice. These results suggested that modification of GSTP1 is a promising therapeutic strategy to counteract CAR-T cell exhaustion and improve antitumor efficacy.

## Materials and methods

### Clinical samples

Tumor patient samples (multiple myeloma: *n* = 31, diffuse large B-cell lymphoma: *n* = 30) and healthy donor samples (individuals without underlying diseases: *n* = 45) were obtained from the clinic. Peripheral blood mononuclear cells were isolated by density gradient centrifugation with Ficoll separation medium. Total RNA was extracted from PBMCs using TRIzol (Invitrogen, USA, Waltham, America), and cDNA was synthesized via reverse transcription (Thermo Fisher, USA, Waltham, America). Real-time quantitative PCR (qRT-PCR) was performed to detect the expression levels of GSTP1, PD-1, B-lymphocyte maturation protein 1 (BLIMP1), and glyceraldehyde-3-phosphate dehydrogenase (GAPDH) (SYBR Green, Germany, Waltham, America).

Primer sequences were as follows:

GSTP1: Forward, ACTATGAGGCGGGCAAGGATG; Reverse, GCAGCAAGTCCAGCAGGTTG. PD-1: Forward, ACTGCCGCTTCCGTGTCAC; Reverse, AGGTAGGTGCCGCTGTCATTG. BLIMP1: Forward, TCACAGCAGCCCTGGGAATAC; Reverse, GCGTTCAAGTAAGCGTAGGAGTC. GAPDH: Forward, CAGGAGGCATTGCTGATGAT; Reverse, GAAGGCTGGGGCTCATTT (Sangon, China, Shanghai, China).

All primers were human-specific. This study was approved by the institutional ethics review board of The General Hospital of Ningxia Medical University (approval number: KYLL-2025-1370). The study was conducted in accordance with the guidelines of the Declaration of Helsinki and relevant regulations. Verbal informed consent was obtained from all participants, and written informed consent was also provided. The studies were conducted in accordance with local legislation and institutional requirements.

### Isolation of primary CD3^+^ T cells

Peripheral blood from healthy donors was subjected to density gradient centrifugation using lymphocyte isolation medium (Solarbio, China, Beijing, China) to isolate peripheral blood mononuclear cells. After two washes with phosphate-buffered saline (PBS), CD3^+^ T cells were purified using MS Columns (Miltenyi Biotec, Germany, Bergisch Gladbach, Germany) and CD3^+^ magnetic beads (Miltenyi Biotec, Germany). CD3^+^ T cells were then activated with antihuman CD3 (2 μg/mL) and antihuman CD28 (2 μg/mL) (BioGems, USA, Westlake Village, America) for 48 h. The activated CD3^+^ T cells were subsequently used to generate CAR-T cells or to construct an exhaustion model.

### Construction of the T-cell exhaustion model

Firstly, a primary CD3^+^ T cell was isolated and activated twice with antihuman CD3 (2 μg/mL) and antihuman CD28 (2 μg/mL). The CD3^+^ T cells were then stimulated every 2 days with antihuman CD3 (4 μg/mL) and antihuman CD28 (4 μg/mL). The condition of the T cells was monitored to prevent apoptosis due to overstimulation. On day 10, cells were collected to assess PD-1 expression (Absin, China, Shanghai, China) and ROS levels (Solarbio, China) via flow cytometry, and total protein was extracted from T cells to evaluate GSTP1 expression.

### Transduction of 293T cells

Firstly, lentivirus (GENE, China, Shanghai, China) carrying the target gene was used to transfect 293T cells. The 293T cells were cultured in high-glucose DMEM complete medium (Bioscience, China) until they reached optimal conditions. Cells were then seeded in a 24-well culture plate, and lentiviral transduction was performed at a multiplicity of infection (MOI) = 5 (adjusted as needed based on viral titer). RNA and protein were extracted on day 7 posttransduction for subsequent experiments.

### Construction of CAR-T cells

T cells were activated for 48 h and then transduced with lentiviral particles encoding CAR (GENE, China). Retronectin (3.6 μg/mL) was coated on the plate a day before. Each well of a 24-well culture plate was seeded with 3 × 10^5^ T cells and transduced with lentiviral particles at MOI = 6. Polybrene (0.8 μg/mL) was added to enhance transduction efficiency. After centrifugation at 1,000×*g* for 2 h, T cells were cultured for 7 days, and positive CAR-T cells were measured by flow cytometry (BD, USA, Franklin Lakes, America).

### Knockdown of shRNA

The cells were collected, and RNA was extracted using TRIzol (Invitrogen, USA). The RNA was then reverse-transcribed into cDNA (Thermo Fisher, USA), and q-PCR (SYBR Green, Germany) was performed to assess the knockdown efficiency of shGSTP1<ns/>1, shGSTP1<ns/>2, and shGSTP1<ns/>3. The GSTP1 primer sequence was as follows:

Forward, ACTATGAGGCGGGCAAGGATG; Reverse, GCAGCAAGTCCAGCAGGTTG. Target Sequence: shGSTP1<ns/>1, CGCTGACTACAACCTGCTGGA; shGSTP1<ns/>2, CCTCACCCTGTACCAGTCCAA; shGSTP1<ns/>3, ACTCAAAGCCTCCTGCCTATA (Sangon, China).

### Western blot assay

Firstly, cells were collected, and total protein was extracted (KeyGEN Bio TECH, China, Jiangsu, China). A BCA standard curve (KeyGEN Bio TECH, China) was prepared to determine protein concentration. For Western blot analysis, 20 μg of protein samples were mixed with 6 × loading buffer (LabLEAD, Jiangsu, China) and electrophoresed using SDS-PAGE (LabLEAD, Shanghai, China) at 150 V for 80 min, with a 180-kDa protein marker (LabLEAD, China) as a reference. Proteins were transferred to a PVDF membrane (Vazyme, Jiangsu, China) at 400 mA for 30 min. The membrane was blocked with 5% skim milk for 90 min at room temperature and then washed with 1 × Tris-buffered saline with Tween-20 (TBST) (SEVEN, England, Beijing, China) for 30 min. It was subsequently incubated overnight at 4°C with primary antibodies: human antirabbit GAPDH (CST, USA), human antirabbit GSTP1 (Cell Signaling Technology (CST), USA), and human antirabbit BLIMP1 (ProteinTech, Wuhan, China), all diluted 1:1,000 in 5% skim milk. The following day, the membrane was washed with 1 × TBST for 30 min and incubated with goat antirabbit HRP secondary antibody (ProteinTech, China), diluted 1:10,000 in 5% skim milk, for 1 h at room temperature. The membrane was then washed with 1 × TBST for 30 min, and bands were visualized using chemiluminescent substrate (NCM Biotech, Jiangsu, China) on an Amersham Imager 680 instrument.

### ChIP-qPCR assay

293T and T cells were expanded for the chromatin immunoprecipitation followed by quantitative polymerase chain reaction (ChIP-qPCR) experiment, following the Enzymatic Chromatin IP Kit (agarose beads) protocol (CST, USA). The main steps were as follows (1): Cell culture cross-linking and sample preparation (2). Nuclei preparation and chromatin digestion (3). Analysis of chromatin digestion and determination of concentration (4). Chromatin immunoprecipitation using a ChIP-grade BLIMP1 antibody (ProteinTech, China) (5). Elution of chromatin from antibody/protein G agarose beads and reversal of cross-links (6). DNA purification using spin columns (7). Quantification of DNA by qPCR to evaluate the ChIP enrichment efficiency. CHIP-grade GSTP1 primer sequences were as follows: Forward, CTGCTGTCTGTTTACCTAGG; Reverse, CCTGGAGTCCCCGGAGTC (Sangon, China). Amplified PCR products were subjected to agarose gel electrophoresis, using a 100-bp DNA ladder maker as a reference. The gel was run in 1 × TAE buffer at 150 V for 30 min, and an imaging system was used to capture the gel image.

### Cell proliferation assay

Firstly, Daudi cells were pretreated with mitomycin C (10 μg/mL) and K562 cells were pretreated with mitomycin C (30 μg/mL) (America (MCE), Monmouth Junction, USA) for 12 h. CAR-T cells were then stained with 5 μM Cell Trace Far Red reagent (Thermo Fisher, USA) at 37°C in the dark for 25 min. CAR-T cells were cocultured with Daudi or K562 cells at an effector-to-target ratio of 5:1 in T-cell complete culture medium (LOZONA, USA, Walkersville, America) for 5 days. Finally, CAR-T cells were collected, washed once with PBS, resuspended in 500 μL PBS, and analyzed via flow cytometry.

### Flow cytometry analysis

On day 7 post expansion of CAR-T cells, cells were collected and resuspended in PBS at a density of 1 × 10^6^/mL. For the memory phenotype test, 2 × 10^5^ cells per sample were incubated with antibodies, including antihuman CD3, APC (5 μL; Invitrogen, USA); antihuman CD4, RPA-T4-APC (5 μL; Thermo Fisher, USA); antihuman CD8, Per-CP (5 μL; BioLegend, USA, San Diego, America); antihuman BV421, CCR7 (5 μL), (Thermo Fisher, USA); antihuman CD45RA, PE (5 μL; BioLegend, USA); mouse antihuman PD-1 (5 μL; Absin, China); and BV-421 mouse antihuman CD197 (5 μL; BD, USA). Samples were incubated at 4°C in the dark, after which 500 μL of PBS was added to stop the incubation. Cells were centrifuged at 1,500 rpm, the supernatant was discarded, and cells were resuspended in 500 μL of PBS for analysis using a BD Selesta flow cytometer. For the ROS assay (Solarbio, China), cells were adjusted to a density of 1 × 10^6^/mL, and 1 μL of dihydroethidium was added. Cells were incubated at 37°C in the dark for 30 min, followed by the addition of 500 μL of PBS to stop the reaction. Cells were centrifuged at 1,500 rpm, the supernatant was discarded, and cells were resuspended in 500 μL of PBS for flow cytometry analysis with a BD Selesta. For the apoptosis assay (Absin, China), 3 × 10^5^ cells were collected and mixed with 500 μL of solution containing 10 μL 7-AAD and 5 μL Annexin V. The mixture was incubated at room temperature in the dark for 5 min and then analyzed with a BD C6 Plus flow cytometer. For the cell cycle assay (Absin, China), 1 × 10^6^ CAR-T cells were fixed in 1 mL of 75% ethanol at 4°C for over 2 h. After fixation, 500 μL of PBS was added to stop the process, cells were centrifuged to discard the supernatant, washed with 500 μL of PBS, centrifuged to discard the supernatant, washed with and resuspended in 500 μL staining buffer containing 25 μL PI and 10 μL RNase. The mixture was incubated at 37°C in the dark for 30 min and subsequently analyzed using a BD C6 Plus flow cytometer.

### LDH cytotoxicity assay

The cytotoxicity of CAR-T cells was measured using the CytoTOX 96 Non-Radioactive Cytotoxicity Kit (Promega, Beijing, China). CAR-T cells were cocultured with Daudi or K562 cells at effector-to-target (E:T) ratios of 1:1, 5:1, 10:1, and 20:1 in 96-well plates. After 6 h of coculture, 50 μL of substrate and 50 μL of stop solution were added to the supernatants. Absorbance was measured at 450 nm using a Thermo Scientific microplate reader, and the cytotoxicity percentage was calculated according to the kit formula:


$$\begin{array}{c} \text{Cytotoxicity }\left(\%\right)\;\\ =\;[(\text{Experimental OD }\\ -\text{ Spontaneous OD})/\left(\text{Maximum OD }-\text{ Spontaneous OD}\right)]\times 100\% \end{array}$$

### ELISA

CAR-T cells were cocultured with Daudi and K562 cells at an E:T ratio of 10:1 in RPMI 1640 complete medium for 24 h, without adding additional cytokines. After coculture, the supernatants were collected, and the concentrations of IL-2 and IFN-γ were measured according to the manufacturer’s instructions (Absin, China).

### GSSG and GSH assay kits

Oxidized glutathione (GSSG) and GSH assay kits were used to measure the content of GSSG and GSH in CAR-T cells (Nanjing Jiancheng Bioengineering Institute, Jiangsu, China). Cells were lysed using a sonicator (GD-X40) at 100 W with cycles of 5 s on and 5 s off for 10 min. The lysates were then centrifuged at 3,500 rpm for 10 min at 4°C, and the supernatants were collected. Absorbance was measured at 450 nm using a Thermo Scientific microplate reader, and the results were calculated using the following formulas:


$$\begin{array}{c} \text{GSSG }\left(\text{μmol}/\text{L}\right)\;=\;\left[{\text{GSSG}}_{\text{test}}\left({\text{A}}_{2}-{\text{A}}_{1}\right)\right]/\left[{\text{GSSG}}_{\text{standard}}\left({\text{A}}_{2}-{\text{A}}_{1}\right)\right]\\ \times {\text{C}}_{\text{GSSG }(\text{standard})}\times \text{ dilution fold} \end{array}$$

$$\begin{array}{c} \text{GSH }\left(\text{μmol}/\text{L}\right)\;=\;\left({\text{A}}_{\text{test}}-{\text{A}}_{\text{blank}}\right)/\left({\text{A}}_{\text{standard}}-{\text{A}}_{\text{blank}}\right)\;\times {\text{C}}_{\text{standard}}\\ \times \text{ dilution fold} \end{array}$$

### Tumor model *in vivo*

4-6 weeks of female NOD/ShiltJGpt-*Prkdc^em26Cd52^Il2rg^em26Cd22^*/Gpt mice (NCG mice) ( Jicui Biology, Jiangsu, China) were fed for 7 days to adapt the environment, then, they were injected with 1×10^6^ Daudi-luc (expressing firefly luciferase) cells via tail vein. 7 days later, 1.5×10^7^ CAR-T cells were administered through the tail vein. At various time points, *in vivo* imaging was performed using an *in vivo* imaging system (IVIS). Mice were anesthetized with 2% isoflurane in oxygen, and 200 μL of d-luciferin potassium salt (Promega, China) was injected intraperitoneally per mouse. Bioluminescent imaging was conducted 5 min after d-luciferin administration. Mouse survival was recorded throughout the experiment. Euthanasia was performed using a CO_2_ chamber at a flow rate of 30% of the chamber volume per minute if mice exhibited hair loss or rapid weight loss. For CAR-T cell detection in the peripheral blood of mice, orbital blood was collected into heparin-coated tubes, and red blood cell lysis buffer (Solarbio, China) was added. The remaining white blood cells were resuspended in PBS and analyzed for CAR-T cells by flow cytometry. For CAR-T cell detection in the spleen, mice were anesthetized using a CO_2_ chamber at a flow rate of 30% of the chamber volume per minute, and the spleens were harvested. The spleen tissue was ground with a pestle, and the resulting suspension was filtered through a 0.77-µm filter. White blood cells were isolated using Ficoll density gradient centrifugation, and CAR-T cells were analyzed by flow cytometry.

### Ethics approval and consent to participate

All animal experimental procedures were approved by the Ningxia Medical University Medical Institutional Animal Care and Use Committee (Approval number: IACUC-NYLAC-2024-245). The approved study period for the protocol was from December 2024 to April 2025.

### Statistical analysis

All statistical analyses were performed using SPSS 26.0, and graphs were generated with GraphPad Prism version 9.0. Comparisons between two groups were conducted using unpaired two-tailed *t*-test. For comparisons involving three or more groups, one-way analysis of variance (ANOVA) with multiple comparisons was applied. Statistical significance was defined as ^*^ *p* < 0.05, ^**^ *p* < 0.01, ^***^ *p* < 0.001, ^****^ *p* < 0.0001, and not significant (ns) as *p* > 0.05. All data are presented as the mean ± SD from three independent experiments.

## Results

### GSTP1 was downregulated in exhausted T cells

**Figure 1:**
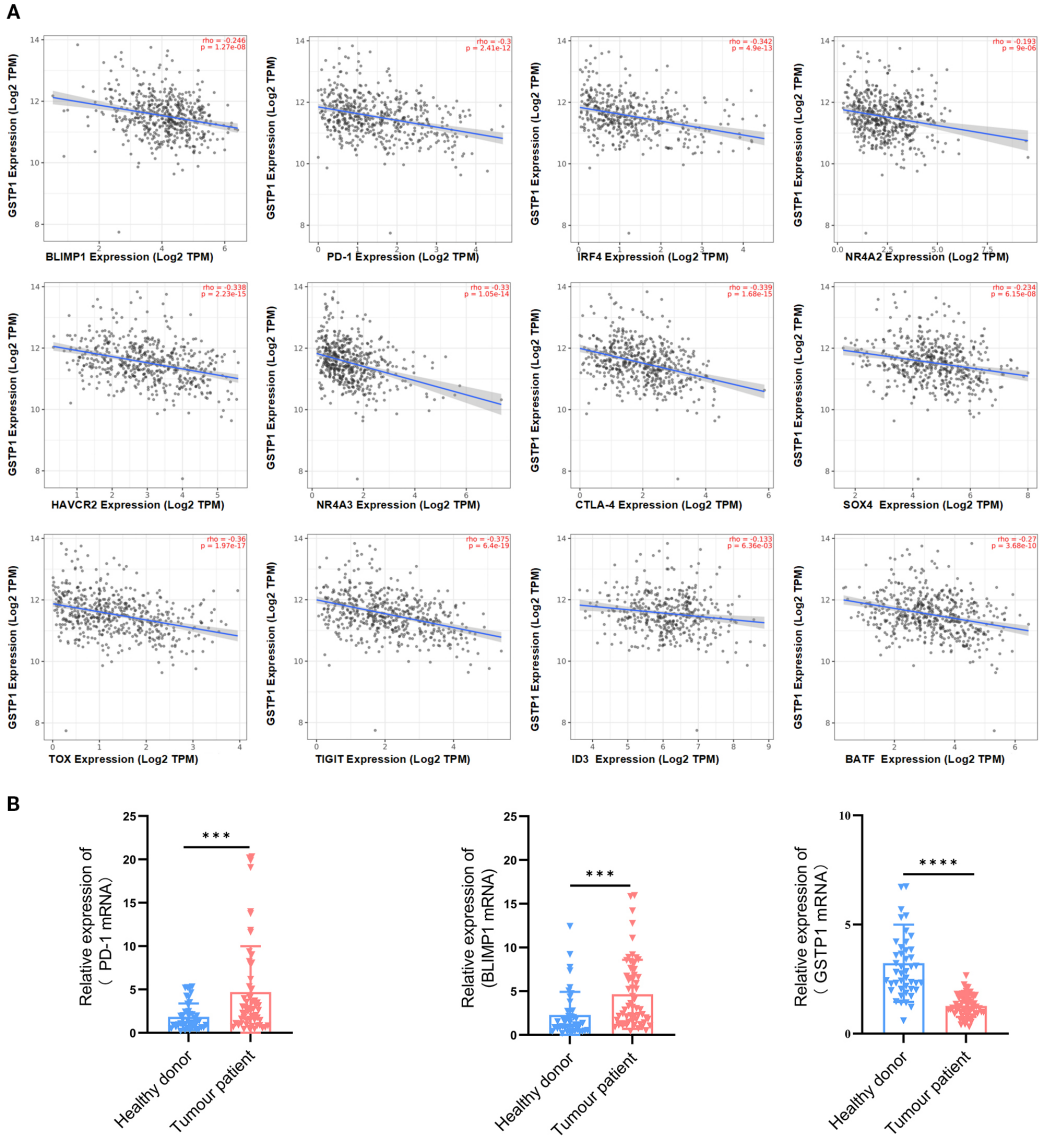
GSTP1 was downregulated in the PBMCs of cancer patients. **(A)** The TIMER database was used to analyze the relationship between GSTP1 and genes associated with T-cell exhaustion. **(B)** Peripheral blood mononuclear cell RNA was extracted from cancer patients (*n* = 61) and healthy individuals (*n* = 45), and GSTP1, PD-1, and BLIMP1 mRNA expression levels were measured by qRT-PCR with three replicates for each sample. An unpaired two-tailed *t*-test was used for comparisons between two groups. ***p < 0.001, ****p < 0.0001.

To clarify the role of GSTP1 in exhausted T cells, we first analyzed the correlation between GSTP1 and genes associated with T-cell exhaustion using the TIMER database. GSTP1 expression was negatively correlated with BLIMP1, HAVCR2, IRF4, NR4A2, PD-1, NR4A3, cytotoxic T-lymphocyte-associated protein 4 (CTLA-4), SOX4, TOX, TIGIT, ID3, and BATF levels (**Figure 1A**). Among these candidates, BLIMP1 was selected for further investigation due to its known function in T-cell exhaustion. PBMCs were collected from patients with hematological malignancies (multiple myeloma: *n* = 31; diffuse large B-cell lymphoma: *n* = 30) to analyze GSTP1 expression. We found that GSTP1 was significantly downregulated in patients compared with healthy donors, whereas the expression of both PD-1 and BLIMP1 was significantly elevated (**Figure 1B**). These findings suggest that low GSTP1 expression may be associated with mechanisms underlying CAR-T cell exhaustion.

**Figure 2:**
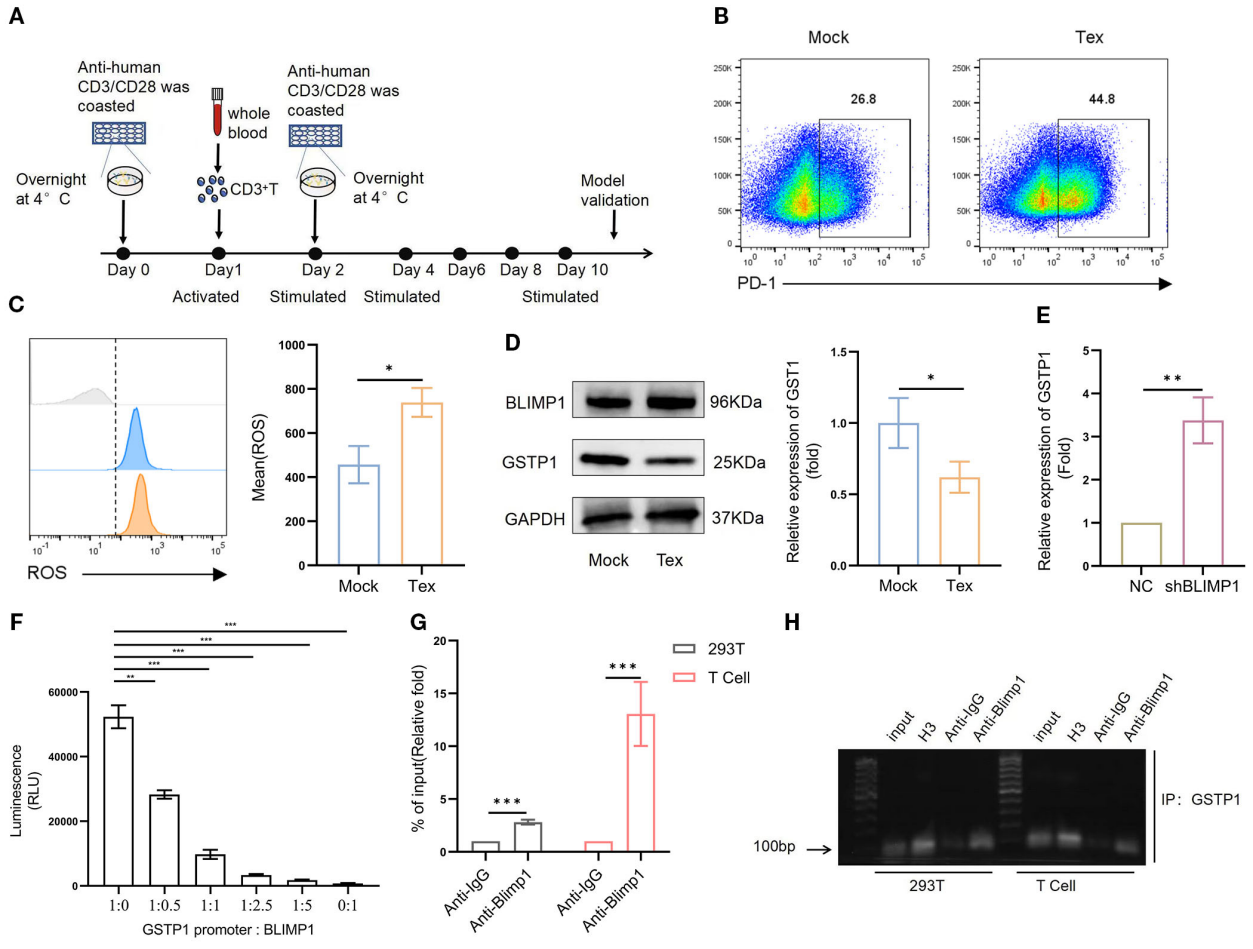
GSTP1 is downregulated in a T-cell exhaustion model. **(A)** CD3^+^ T cells isolated from the peripheral blood of healthy donors were activated using anti-CD3 and anti-CD28 antibodies. After 24 h, cells were reactivated, and the Tex group was subsequently stimulated every 2 days with CD3 and CD28. **(B)** On day 10, T cells were collected to validate the exhaustion model, and flow cytometry was used to assess PD-1 expression (*n* = 3). **(C)** ROS levels were measured using a DHE probe by flow cytometry (*n* = 3). **(D)** Western blotting was used to detect GSTP1 expression in the T-cell exhaustion model (*n* = 3). **(E)** shBLIMP1 was transduced into T cells, and GSTP1 expression was measured by qRT-PCR (*n* = 3). **(F)** BLIMP1 and GSTP1 plasmids were cotransfected into 293T cells at different ratios, and a dual-luciferase assay was used to test the activity of the GSTP1promoter (*n* = 3). **(G)** For the ChIP-qPCR assay, after culturing sufficient quantities of 293T and T cells, chromatin immunoprecipitation was performed to verify the binding of transcription factor BLIMP1 to the GSTP1promoter region. H3 was used as a positive control, anti-IgG as the negative control, and anti-BLIMP1 as the experimental group (*n* = 3). **(H)** ChIP-PCR products analyzed by nucleic acid electrophoresis were used to further confirm that transcription factor BLIMP1 bound to the GSTP1 promoter (*n* = 3). The unpaired two-tailed *t*-test was used for comparisons between two groups. *p < 0.05, **p < 0.01, ***p < 0.001.

To verify this hypothesis, we established an *in vitro* T-cell exhaustion model (14–16). Specifically, activated CD3^+^ T cells were stimulated with antihuman CD3/CD28 every 2 days for a total of 10 days (**Figure 2A**). On day 10, T cells were collected, and PD-1 expression was analyzed to evaluate exhaustion. The results showed that PD-1 expression in the stimulated group was significantly increased, confirming the successful establishment of the T-cell exhaustion model (**Figure 2B**). Subsequently, ROS levels were significantly elevated (**Figure 2C**), and GSTP1 was markedly reduced in the exhausted group (**Figure 2D**). In addition, BLIMP1 was highly expressed in exhausted T cells, consistent with the findings in clinical samples. BLIMP1 is a key transcriptional regulator that critically controls immune cell differentiation and function, playing essential and multifaceted roles in T-cell development, activation, and exhaustion processes (17, 18). We hypothesized that BLIMP1 may inhibit GSTP1 expression at the transcriptional level. Transduction of shBLIMP1 into T cells resulted in upregulation of the GSTP1 gene expression (**Figure 2E**). A dual-luciferase assay was used to further verify the relationship between BLIMP1 and GSTP1, and the results indicated that BLIMP1 suppressed the activity of the GSTP1 promoter (**Figure 2F**). Based on analysis from the JASPAR database, we inferred that the transcription factor BLIMP1 may bind to the GSTP1 promoter. We used ChIP-qPCR to identify the binding site of BLIMP1 on the GSTP1 promoter and found that it was located in the upstream region from – 1,788 to – 1,802 bp (**Figures 2G, H**). BLIMP1 was significantly bound to the GSTP1 promoter, indicating that GSTP1 is related to exhaustion in T-cell exhaustion and is negatively regulated by BLIMP1.

### Generation of GSTP1 CAR-T cell workflow

**Figure 3:**
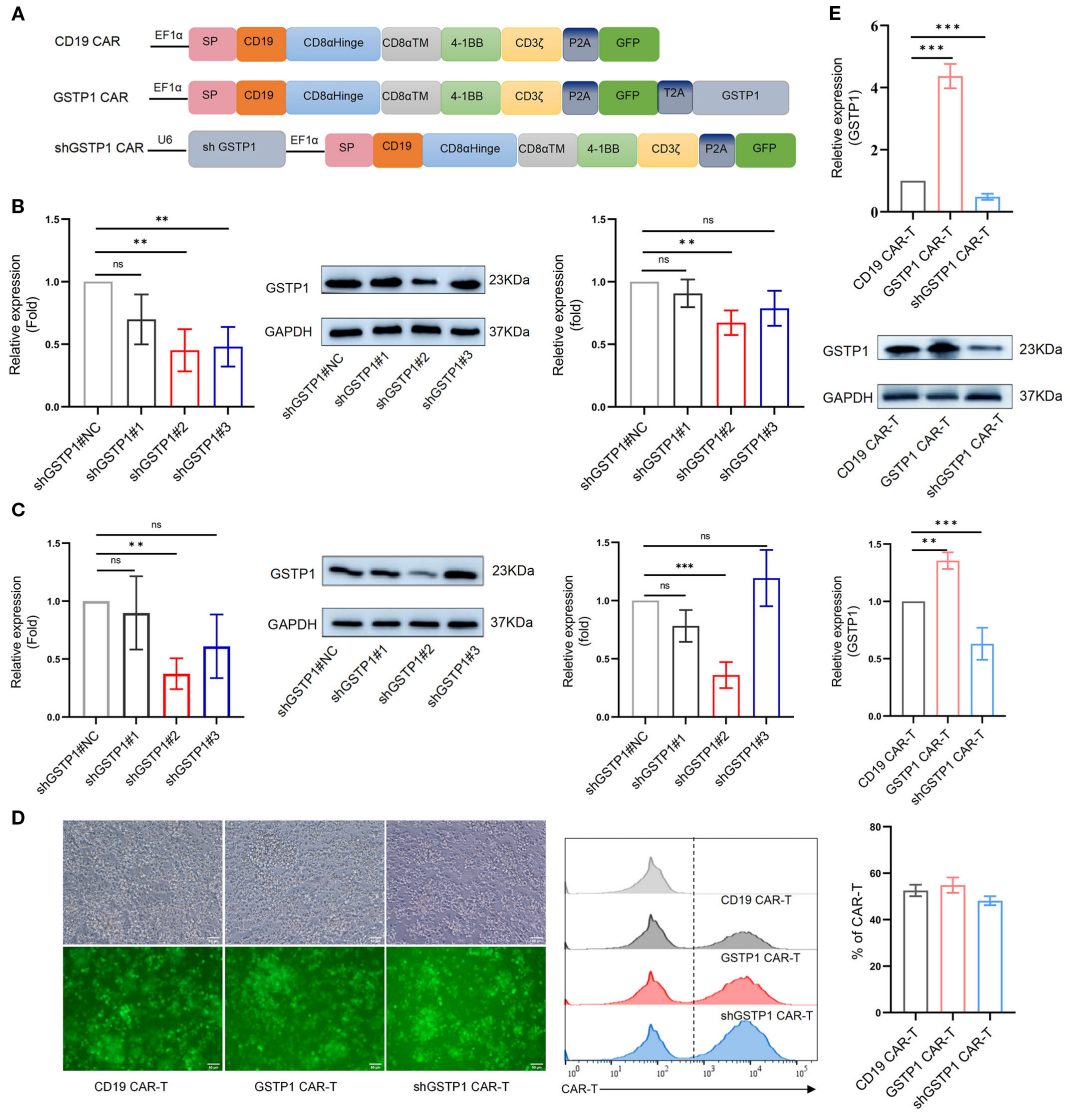
The CAR-T cells were constructed. **(A)** The structural framework of CD19 CAR, GSTP1 CAR, and shGSTP1 CAR. **(B)** Lentivirus-packaged shGSTP1<ns/>1, shGSTP1<ns/>2, and shGSTP1<ns/>3 were transfected into 293T cells to assess the knockdown efficiency of GSTP1, from left to right: qRT-PCR validation, Western blotting (WB) verification, and grayscale value analysis of WB (*n* = 3). **(C)** Lentivirus-packaged shGSTP1<ns/>1, shGSTP1<ns/>2, and shGSTP1<ns/>3 were transfected into primary T cells to evaluate the knockdown efficiency of GSTP1, from left to right: qRT-PCR validation, WB verification, and grayscale value analysis of WB (*n* = 3). **(D)** Three types of CAR-T cells were successfully constructed to test the expression of GFP using fluorescence imaging, and flow cytometry was applied to test the percentage of the three types of CAR-T cells (*n* = 3). **(E)** The overexpression efficiency of GSTP1 was verified in CAR-T cells. From top to bottom: qRT-PCR validation, WB verification, and grayscale value analysis of WB (*n* = 3). Experiments were repeated independently three times. The unpaired two-tailed *t*-test was used for comparisons between two groups. ^*^ *p* < 0.05; **P < 0.01, ***P < 0.001 and not significant (ns) is *P* > 0.05.

To elucidate the role of GSTP1 in CAR-T cells, we generated three distinct variants of CD19-targeted CAR-T cells: control CD19 CAR-T cells, GSTP1 CAR-T, and GSTP1 knockdown CAR-T cells (shGSTP1 CAR-T) (**Figure 3A**). For the construction of shGSTP1 CAR-T cells, we first validated the efficiency of shRNA in 293T cells and T cells, respectively. The preliminary results showed that shGSTP1#2 significantly decreased the expression of GSTP1 by approximately 60%, which was sufficient for the subsequent experiments (**Figures 3B, C**). A high transduction rate and positive percentage of CAR-T cells of approximately 60% were generated (**Figure 3D**). Meanwhile, GSTP1 overexpression was also observed at the mRNA and protein levels (**Figure 3E**). These results verified that we successfully constructed CD19, GSTP1, and shGSTP1 CAR-T cells.

### GSTP1 CAR-T cells exhibited an advantage in memory phenotype

**Figure 4:**
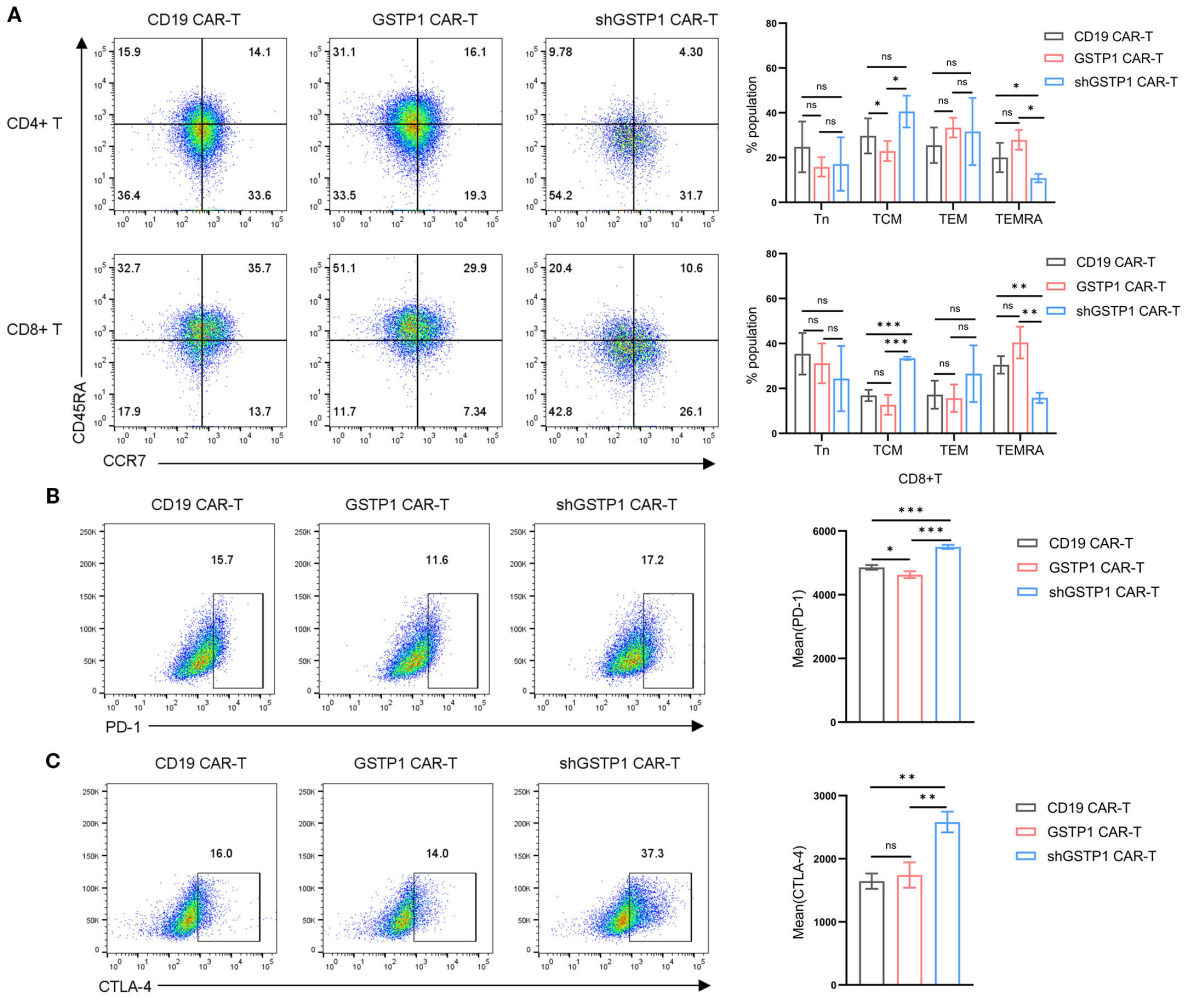
The proportion of the terminal effector memory subset increased in GSTP1 CAR-T cells, and the immune inhibitory marker increased in the shGSTP1 CAR-T cells. **(A)** After 7 days of CAR-T cell culture, cells were collected to test markers CCR7 and CD45RA by flow cytometry (*n* = 3). The expression of CD45RA and CCR7 was analyzed within the populations of CD4^+^CAR-T and CD8^+^CAR-T cells, respectively (Tn: CD45RA^+^CCR7^+^; TCM: CD45RA^−^CCR7^+^; TEM: CD45RA^−^CCR7^−^; TEMRA: CD45RA^+^CCR7^−^). **(B**, **C)** After 7 days of CAR-T cell expansion, cells were collected and analyzed by flow cytometry to detect PD-1 and CTLA-4 expression levels (*n* = 3). The unpaired two-tailed *t*-test was used for comparisons between two groups. *P < 0.05, **P < 0.01, ***P < 0.001 and not significant (ns) is *P* > 0.05.

Considering that the memory population is essential for the self-renewal of T cells (3), we explored the role of GSTP1 in the shift of the memory phenotype within CAR-T cells. The terminally differentiated effector memory T-cell (TEMRA) population increased in CD4^+^GSTP1 CAR-T cells and CD8^+^GSTP1 CAR-T cells, despite the lack of significant statistical differences (**Figure 4A**). TEMRA is responsible for an acute immune reaction in the target cell. This phenomenon suggested that GSTP1 endowed an antitumor advantage to CAR-T cells. We also examined the expression of the immune inhibitory checkpoint molecules PD-1 and CTLA-4 in GSTP1 CAR-T cells. The results showed that the expression of PD-1 decreased slightly in the GSTP1 CAR-T group, while it increased significantly in the shGSTP1 CAR-T group (**Figures 4B, C**). These preliminary results illustrated that overexpressing GSTP1 may enhance the antitumor ability of CAR-T cells by conferring a memory phenotype advantage.

### GSTP1 augmented the proliferation capacity of the CAR-T cells

**Figure 5:**
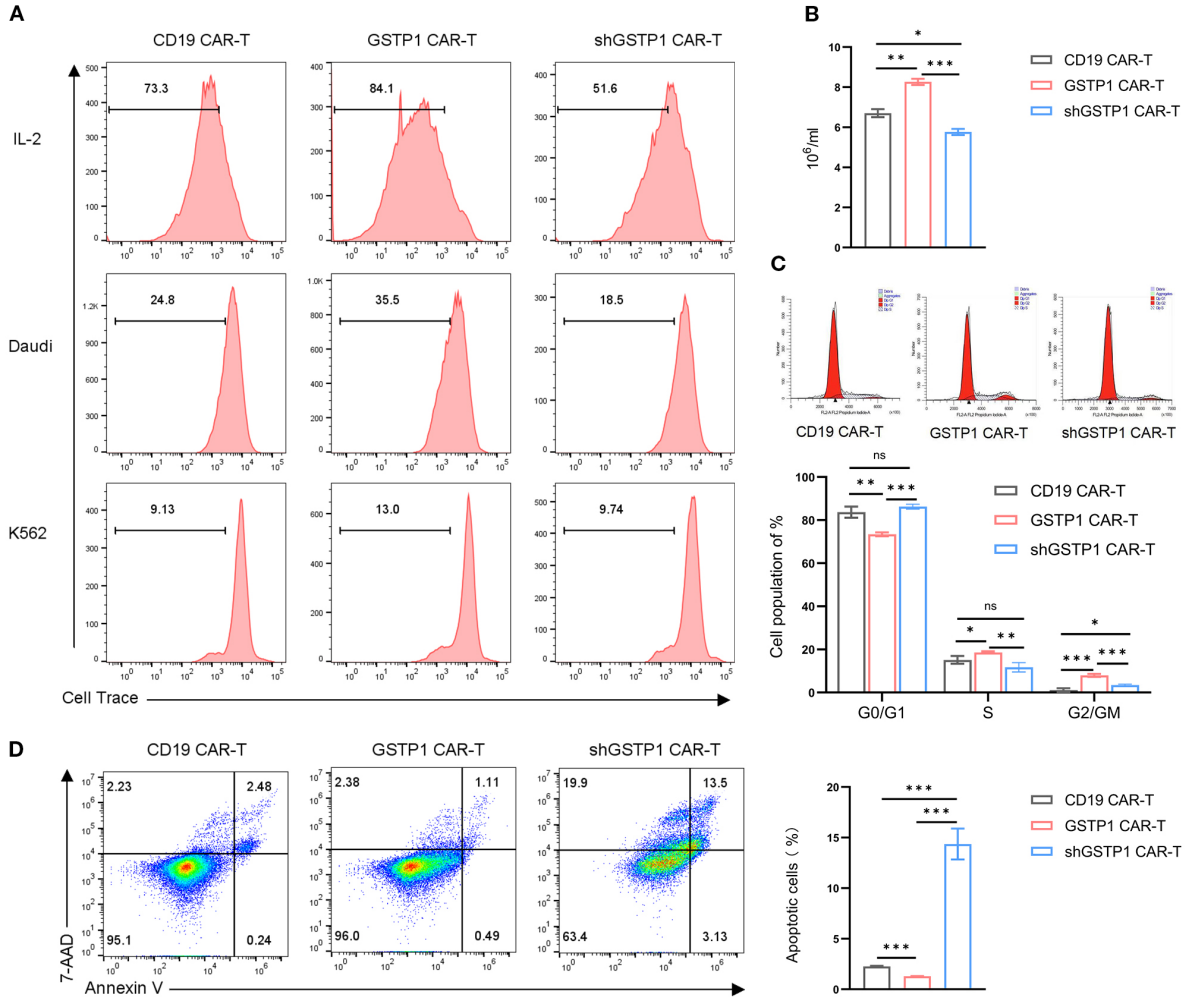
Proliferation capacity was enhanced and apoptosis improved in GSTP1 CAR-T cells. **(A)** CAR-T cells were cultured in complete medium and stained with Cell Trace Far Red proliferation dye. After 5 days, CAR-T proliferation was assessed by flow cytometry (top row, including IL-2). Daudi and K562 cells were pretreated with puromycin C for 12 h and then cocultured with CAR-T cells and Daudi/K562, respectively, at an effector-to-target (E:T) ratio of 5:1 (no IL-2 addition). Before coculture, CAR-T cells were collected and stained with Cell Trace Far Red proliferation dye. After 5 days of coculture, the proliferative capacity of CAR-T cells was assessed by flow cytometry. **(B)** CAR-T cells (4 × 10^5^ cells per group were seeded for the assay) were expanded for 7 days in the presence of IL-2, and the number of CAR-T cells was counted using a microscope in three independent experiments (*n* = 3). **(C)** After 7 days of CAR-T cell expansion, cells were stained with propidium iodide (PI) cell cycle reagent, and cell cycle changes were examined within the CAR-T cell population (*n* = 3). **(D)** After 7 days of CAR-T cell expansion, flow cytometry was performed to assess apoptosis in CAR-T cells (*n* = 3). The experiments were repeated three times independently. The unpaired two-tailed *t*-test was used for comparisons between two groups. *P < 0.05, **P < 0.01, ***P < 0.001 and not significant (ns) is *P* > 0.05.

To determine the impact of GSTP1 on the proliferative capacity of CAR-T cells, we cocultured Daudi/K562 cells and CAR-T cells to investigate the antigen-dependent proliferation ability of CAR-T cells. The proliferation of GSTP1 CAR-T cells improved significantly after stimulation by Daudi cells. As expected, when CAR-T cells were stimulated by K562 cells, the proliferative capacity of CAR-T cells disappeared due to the lack of CD19 antigen expression in K562 cells (**Figure 5A**). Moreover, we found that after 7 days of amplification, a higher proliferative capacity was observed in the GSTP1 CAR-T group than in the CD19 CAR-T group. However, this advantage was abolished once GSTP1 was silenced (**Figure 5B**). Additionally, we examined the cell cycle of different CAR-T cells; the results revealed that the proportion of cells in the G0/G1 phase was reduced, and the proportion of cells in the S and G2/M phases increased in the GSTP1 CAR-T group. This result was consistent with the phenomenon of enhanced proliferation ability in GSTP1 CAR-T. However, the proportion of cells in the S phase was reduced after GSTP1 knockdown, which explained why the proliferative capability was slower in the shGSTP1 CAR-T group (**Figure 5C**). In addition, we also found that apoptotic cells increased significantly in the shGSTP1 CAR-T group, while the number of apoptotic cells was the lowest in the GSTP1 CAR-T group (**Figure 5D**). The above results indicated that GSTP1 not only enhanced the proliferation ability but also reduced the apoptosis of CAR-T cells.

### GSTP1 strengthened the *in vitro* antilymphoma suppression of CAR-T cells

**Figure 6:**
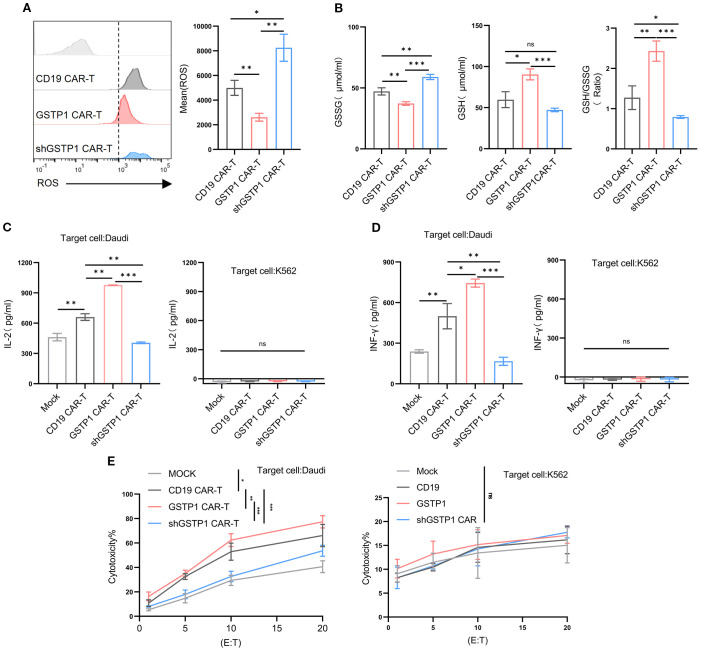
GSTP1 CAR-T improved cytotoxicity, increased secretion of IL-2 and IFN-γ, and enhanced antioxidative capacity. **(A)** After 7 days of CAR-T cell expansion, cells were stained with a DHE probe and analyzed by flow cytometry to detect ROS levels in CD19 CAR-T, GSTP1 CAR-T, and shGSTP1 CAR-T cells (*n* = 3). **(B)** After 7 days of CAR-T cell expansion, CAR-T cells (5.0 × 10^6^ cells per group) were collected and lysed using a sonicator. The lysates were centrifuged at 3,500 rpm for 10 min, and the supernatants were subjected to a colorimetric reaction. OD values were measured at 450 nm to calculate the GSSG and GSH content (*n* = 3). **(C)** CAR-T cells were cocultured with Daudi/K562 cells at an effector-to-target ratio of 10:1 for 24 h, and coculture supernatants were collected for ELISA to detect IL-2 secretion of CAR-T cells (*n* = 3). **(D)** CAR-T cells were cocultured with Daudi/K562 cells at an effector-to-target ratio of 10:1 for 24 h, and supernatants were collected for ELISA to detect IFN-γ secretion of CAR-T cells (*n* = 3). **(E)** CAR-T cells were cocultured with Daudi/K562 cells at different effector-to-target ratios (20:1, 10:1, 5:1, 1:1) for 6 h, and coculture supernatants were collected to assess the cytotoxic capacity of CAR-T cells by measuring LDH content (*n* = 3). All experiments were performed with three independent replicates. The unpaired two-tailed *t*-test was used for comparisons between two groups. *P < 0.05, **P < 0.01, ***P < 0.001 and not significant (ns) is *P* > 0.05.

Considering that GSTP1 may mediate antioxidant activity to increase the cytotoxicity of T cells, we first measured ROS levels of CAR-T cells in each group. GSTP1 neutralized ROS levels within CAR-T cells; however, ROS levels in the shGSTP1 CAR-T group rose remarkably (**Figure 6A**). CAR-T cells were collected to test GSSG and reduced GSH levels: GSSG was decreased in the GSTP1 CAR-T group compared with the CD19 CAR-T group, while GSH levels increased in the GSTP1 CAR-T group (**Figure 6B**). GSH and GSSG collectively maintain the redox balance within cells. GSH primarily functions as an antioxidant and provides cellular protection, whereas GSSG is generated under oxidative stress conditions and is subsequently reduced to regenerate GSH, sustaining the cellular redox state (13). Next, we cocultured CAR-T cells with tumor cells (Daudi or K562) at an effector-to-target ratio of 10:1. After 24 h of coculture, ELISA was used to measure the secretion of IFN-γ and IL-2 in different CAR-T cells. The levels of IFN-γ and IL-2 were amplified in GSTP1 CAR-T cells, whereas those of IFN-γ and IL-2 in shGSTP1 CAR-T cells were reduced. In the context of K562 cells, GSTP1 CAR-T cells showed a deficiency of secretion of IFN-γ and IL-2 (**Figures 6C, D**). In addition, lactate dehydrogenase (LDH)-based assays were used to assess the cytotoxicity of different CAR-T cells. The results showed that GSTP1 CAR-T cells significantly improved antitumor cytotoxicity in Duadi cells, whereas shGSTP1 CAR-T cells exhibited inferior antitumor competence. After coculture with K562 cells, no difference in cytotoxicity was observed among the three types of CAR-T cells (**Figure 6E**). These results underlined that GSTP1 indeed reinforced the antioxidant capacity of CAR-T cells and strengthened the antilymphoma effects *in vitro.*

### GSTP1 enhanced the *in vivo* antilymphoma capacity of CAR-T cells

**Figure 7:**
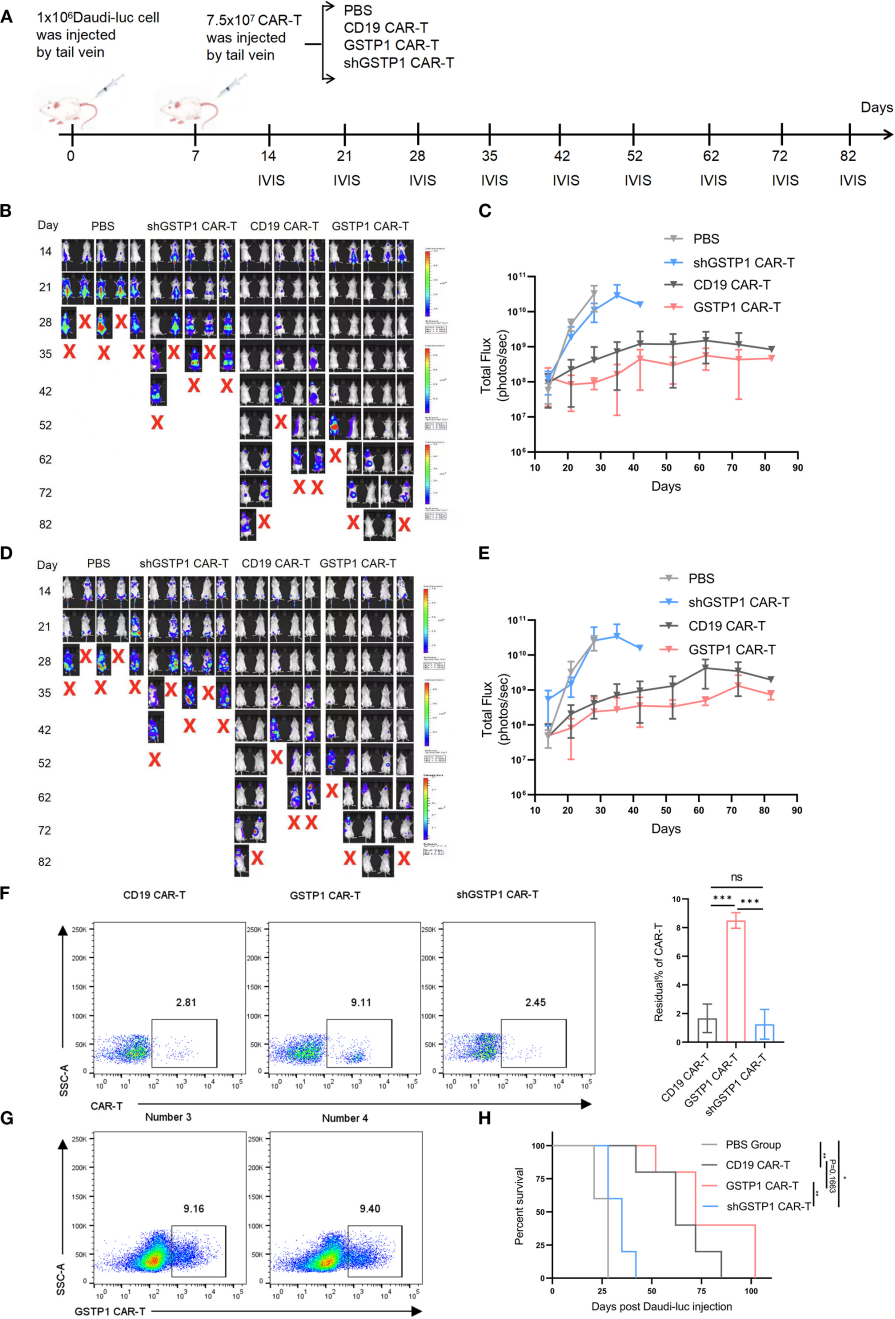
GSTP1 CAR-T cells have an advantage for antitumor capacity *in vivo*. **(A)** Flow chart of the experimental protocol of NCG mice injected with Daudi-luc cells and CAR-T cells. **(B**, **C)** Fluorescence quantification graphs of the dorsal region in four mouse groups; mice were imaged by IVIS at different time points (*n* = 5). **(D**, **E)** Fluorescence quantification graphs of the belly region in four mouse groups; mice were imaged by IVIS at different time points (*n* = 5). **(F)** On day 30 after the injection of Daudi-luc cells, orbital venous blood samples from three mice per group (CD19 CAR-T, GSTP1 CAR-T, and shGSTP1 CAR-T group) were collected. After red blood cells were lysed, flow cytometry was used to detect the residual CAR-T cells in each mouse (*n* = 3). **(G)** Two mice in the GSTP1 CAR-T group were dissected after anesthetization, and their spleens were harvested. The spleen tissue was ground with a pestle and sieved through a 0.77-µm filter. The cell suspension was subjected to Ficoll density gradient centrifugation, and the leukocyte layer was collected and analyzed by flow cytometry to assess the residual CAR-T cell population in the spleen (*n* = 2). **(H)** Mouse survival was recorded (*n* = 5). *P < 0.05, **P < 0.01, ***P < 0.001 and not significant (ns) is *P* > 0.05.

To evaluate the antitumor efficacy of GSTP1 CAR-T cells *in vivo*, a mouse lymphoma model was established by injecting 1.0 × 10^6^ Daudi-luc cells through the tail vein. After 7 days, 7.5 × 10^7^ CAR-T cells were infused in the same manner (**Figure 7A**). The tumor load was monitored by IVIS, and the results showed that mice treated with shGSTP1 CAR-T cells died in a short time because high ROS levels weakened the antitumor function of CAR-T cells (**Figures 7B–E**). Conversely, GSTP1 CAR-T showed extraordinary antitumor ability. On the 30th day after injection, orbital venous blood was collected to detect residual CAR-T cells. Abundant CAR-T cells remained in mice treated with GSTP1 CAR-T cells, whereas the number of CAR-T cells was minimal in mice treated with shGSTP1 CAR-T cells (**Figure 7F**). On day 102, spleens were harvested from two mice, and GSTP1 CAR-T cells were isolated for flow cytometry analysis. The results showed that almost 10% of GSTP1 CAR-T cells were still present (**Figure 7G**), which contributed to the prolonged survival of mice treated with GSTP1 CAR-T cells (**Figure 7H**). Altogether, these results revealed that GSTP1 enhanced the antitumor capacity of CAR-T cells *in vivo* and extended the survival rate of mice.

## Discussion

CAR-T cell immunotherapy is a revolutionary cellular treatment with significant antitumor effects. However, CAR-T cell exhaustion limits its efficacy and application (19). Current strategies to address CAR-T exhaustion mainly include optimizing the structure of CAR-T cells and using gene editing to incorporate 4-1BB and CD28 costimulatory molecules into CAR structures, to enhance the cytotoxic capacity of T cells activated by antigen (20). Most studies have focused on attenuating the exhaustion of CAR-T by gene editing to knock down or overexpress transcription factors to achieve stronger antitumor capacity (21). Combination with immune checkpoint inhibitors, such as PD-1 monoclonal antibodies, with CAR-T cells can prolong the survival of T cells and increase their antitumor ability. Similarly, engineering CAR-T cells to secrete PD-1 antibody single-chain variable fragments can enhance antitumor activity comparably to PD-1 antibody/CAR-T combinations (22). Furthermore, improving the tumor microenvironment, blocking immunosuppressive metabolic signals, targeting LDH, or inhibiting amino acid-modifying enzymes in the tumor microenvironment can enhance the efficacy of CAR-T cells (23).

Studies have confirmed that T-cell receptor (TCR) signaling increases intracellular ROS levels in activated T cells (24). ROS, known participants in the TCR signaling pathway and primarily generated in mitochondria, are crucial for the production of IL-2 (25). Moderate levels of ROS can act as signaling molecules to regulate the function of mitochondria (9). ROS can activate transcription factors such as peroxisome proliferator-activated receptor gamma coactivator-1alpha and promote the expression of genes related to mitochondrial biogenesis, increasing mitochondrial numbers to adapt to the demands of cellular energy (26). However, excessive ROS levels damage the structure of the mitochondrial molecule, triggering lipid peroxidation and disrupting the integrity and fluidity of the membrane, thereby weakening normal mitochondrial function (27, 28). Additionally, ROS oxidizes mitochondrial proteins and leads to loss of function, such as altering key proteins (NADH dehydrogenase, cytochrome *b-c1* complex) in the electron transport chain, reducing the efficiency of electron transfer, and further increasing ROS production to form a vicious cycle (29). GSTP1, a member of the glutathione *S*-transferase family, catalyzes intracellular antioxidant and detoxification reactions by conjugating the thiol group of cysteine, a key component of glutathione, with electrophilic compounds. Studies have shown that chronic antigen stimulation and hypoxia lead to significant ROS accumulation in T cells (30). Elevated ROS levels improve the nuclear translocation of phosphorylated tyrosine signaling and the nuclear factor of activated T cells, driving T cells toward an exhaustion state (31).

GSTP1 plays multiple roles within cells. DDB1- and CUL4-associated factor 1 can upregulate GSTP1 expression, inhibiting ROS accumulation and regulatory T-cell aging. This mechanism highlights the importance of GSTP1 in maintaining immune homeostasis and delaying the senescence of immune cells (31, 32). In the tumor microenvironment, which is often characterized by hypoxia or glucose deprivation, GSTP1 noncovalently binds to glucose-6-phosphate dehydrogenase, inhibiting its phosphorylation and improving pentose phosphate pathway activity and NADPH production. This process helps tumor cells resist oxidative stress (33, 34). GSTP1 protects cells from ferroptosis through GSH conjugation and selenium-independent GSH peroxidase activity, a mechanism that is independent of known ferroptosis defense systems such as GPX4, FSP1, and DHODH. SMAD-specific E3 ubiquitin protein ligase 2 promotes the sensitivity of cancer cells to ferroptosis by mediating GSTP1 ubiquitination and degradation (35). Similar enhancements in T-cell and CAR-T cell persistence and cytotoxicity have been achieved through overexpression of other antioxidant enzymes such as catalase, augmentation of glutathione metabolism, and modulation of the thioredoxin system (such as TRX1 and TRXR1) (36–38). The high expression of GSTP1 in tumor cells leads to resistance to treatment, essentially due to its ability to clear ROS through multiple pathways, alleviating oxidative stress in tumor cells and promoting their proliferation (39). This phenomenon of enhancing cellular vitality is also observed in tumor cells. Overexpression of GSTP1 in CAR-T cells to enhance their viability against tumors may represent a viable treatment strategy.

In this study, the dual-luciferase assay demonstrated that cotransfection of BLIMP1 and GSTP1 plasmids suppressed the activity of the GSTP1 promoter, whereas ChIP-qPCR confirmed that the transcription factor BLIMP1 bound the GSTP1 promoter. Since BLIMP1 is a key transcription factor within the cell (17), it inhibited the transcription of the GSTP1 promoter at the transcriptional level. After transducing T cells with shBLIMP1, the expression of the *GSTP1* gene was increased. Our study demonstrated that BLIMP1 is indeed bound to GSTP1, and the suppression of GSTP1 was due to the high expression of BLIMP1 to some extent. One main reason for the rapid proliferation of tumor cells is the increased expression of GSTP1. Rapid proliferation leads to the generation of excessive peroxides, which are immediately cleared by GSTP1 (40). Consequently, GSTP1 overexpression in CAR-T cells promoted the proliferative capacity of T cells, enabling rapid expansion of tumor-specific T-cell populations and facilitating the timely elimination of tumor cells (41). Interestingly, GSTP1 overexpression increased the proportion of TEMRA, although central memory T cells (TCM) serve as the primary phenotype for maintaining T-cell self-renewal and play a crucial role in mitigating CAR-T cell exhaustion. Our findings revealed that GSTP1 overexpression did not enhance the TCM population in CAR-T cells. Instead, it promoted expansion of TEMRA, and this population was characterized by remarkable cytotoxicity to eliminate the tumor (42). The higher proportion of TCM population in the shGSTP1 CAR-T group might be due to inhibition of cell proliferation after GSTP1 was knocked down, causing T cells to remain in a quiescent state. The S phase was reduced after GSTP1 was knocked down in CAR-T cells; this finding indicated that the proliferation of shGSTP1 CAR-T cells was arrested (11). However, this inhibition was long-term and did not lead to proliferation even upon restimulation by antigen. GSTP1 overexpression did not significantly downregulate PD-1 and CTLA-4 expression levels; however, shGSTP1 CAR-T cells exhibited remarkable upregulation of both PD-1 and CTLA-4. GSTP1 knockdown led to intracellular accumulation of diverse oxidative metabolic byproducts. Through evaluation of the GSH/GSSG ratio, we effectively monitored the oxidative stress status within CAR-T cells. Typically, an elevated intracellular GSH/GSSG ratio indicates a reduced cellular state, whereas a decreased ratio reflects increased oxidative stress. Maintaining redox homeostasis is crucial for the normal physiological functions of cells (10). Redox imbalance could disrupt ATP synthesis during reductive stress, and an increased NADH/NAD^+^ ratio shifts cellular metabolism from mitochondrial respiration toward glycolysis, resulting in insufficient energy production (30, 43).

Given that oxidative stress is just one of many factors contributing to T-cell exhaustion, GSTP1 was likely only partially responsible for mitigating exhaustion. Therefore, a comprehensive evaluation is needed to determine whether enhancing antioxidant capacity alone can alleviate exhaustion. To further optimize CAR-T cell performance, alternative strategies, such as supplementing culture medium with antioxidant compounds like *N*-acetylcysteine or administering antioxidant drugs *in vivo*, may prove more effective. In addition, due to the potential for recurrence after tumor treatment, model validation for recurrence should also be conducted. For mice with high tumor burden, reducing the dose or using a fractionated dosing approach can decrease the risk of CRS and other toxicities (44, 45). Finally, mitochondria are a primary intracellular source of ROS. Considering that GSTP1 plays a central role in redox homeostasis, further research is needed to clarify how GSTP1 affects this process in T cells. GSTP1 was highly expressed in tumor cells and promoted glycolysis. We investigated the influence of GSTP1 on metabolism. We observed that GSTP1 CAR-T achieved a slight up-regulation of key glycolytic enzymes (GLUT1 and LDHA), whereas shGSTP1 CAR-T significantly upregulated glycolysis (data not shown). We postulated that the mild increase in glycolytic enzymes in GSTP1 CAR-T cells may result from accelerated proliferation, which demands a higher energy consumption to support biosynthetic pathways (40). In contrast, the pronounced upregulation of glycolysis pathways in shGSTP1 CAR-T cells could be attributed to mitochondrial damage induced by oxidative stress caused by GSTP1 knockdown. This metabolic shift reflected a compensatory mechanism in which cells increased glucose uptake to sustain ATP production, as impairing mitochondrial function reduced the efficiency of oxidative phosphorylation (46). In future studies, we will further explore the role of GSTP1 in T cells from the perspective of protein–protein interactions. Understanding these mechanisms will provide deeper insight into the regulation of T-cell metabolism and function. This knowledge will aid in the generation of strategies to optimize CAR-T cell therapy by enhancing their resistance to oxidative stress and improving their therapeutic efficacy.

## Conclusion

Our study revealed that the transcription factor BLIMP1 negatively regulated the expression of the GSTP1 promoter, which was implicated in the exhaustion of CAR-T cells. The elimination of GSTP1 significantly promoted the exhaustion of CAR-T cells, highlighting its critical role in maintaining T-cell functionality. In contrast, GSTP1 overexpression not only mitigated oxidative stress but also enhanced the antitumor efficacy of CAR-T cells. These findings illustrate that modulating GSTP1 expression could serve as a promising strategy to optimize CAR-T cell immunotherapy.

## Data Availability

The raw data supporting the conclusions of this article will be made available by the authors, without undue reservation.

## Glossary

CAR-T: chimeric antigen receptor T cells
GSTP1: glutathione *S*-transferase-Pi 1
BLIMP1: B-lymphocyte maturation protein 1
ROS: reactive oxygen species
PBMCs: peripheral blood mononuclear cells
LDH: lactate dehydrogenase
qRT-PCR: real-time quantitative PCR
ELISA: enzyme-linked immunosorbent assay
WB: Western blotting
PD-1: programmed cell death protein 1
CTLA-4: cytotoxic T-lymphocyte-associated protein 4
scFv: single chain antibody fragment
TCM: central memory T cell
TEM: Effector memory T cell
Tn: naïve T cell
GSSG: Oxidized glutathione
GSH: reduced glutathione
IVIS: *in vivo* imaging system
FSP1: ferroptosis suppressor protein 1
TCR: T-cell receptor
NF-κB: nuclear factor kappa light chain enhancer of activated B cells
PGC-1α: peroxisome proliferator activated receptor gamma coactivator 1-alpha
GPX4: glutathione peroxidase 4
DHODH: dihydroorotate dehydrogenase
SMURF2: SMAD specific E3 ubiquitin protein ligase 2
NADH: nicotinamide adenine dinucleotide
NAD^+^: nicotinamide adenine dinucleotide.
