# Supplementary material for: GSTP1 improves CAR-T cell proliferation and cytotoxicity to combat lymphoma
Source: Front Immunol. 2025 Sep 26;16:1665407. doi: 10.3389/fimmu.2025.1665407 (PMC12511145; doi:10.3389/fimmu.2025.1665407)

First time

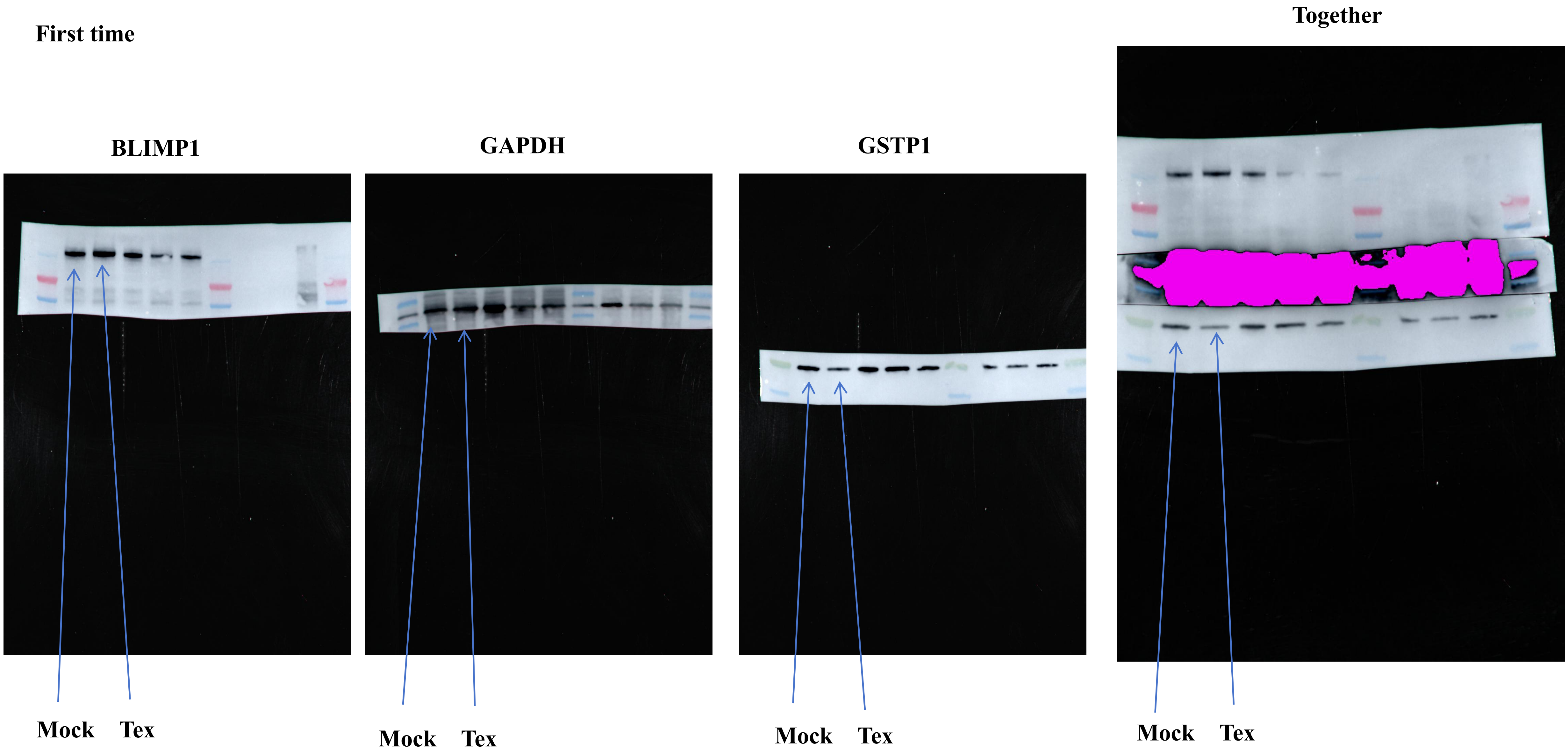
BLIMP1
Mock
Tex
GAPDH
Mock
Tex
GSTP1
Mock
Tex
Together
Mock
Tex

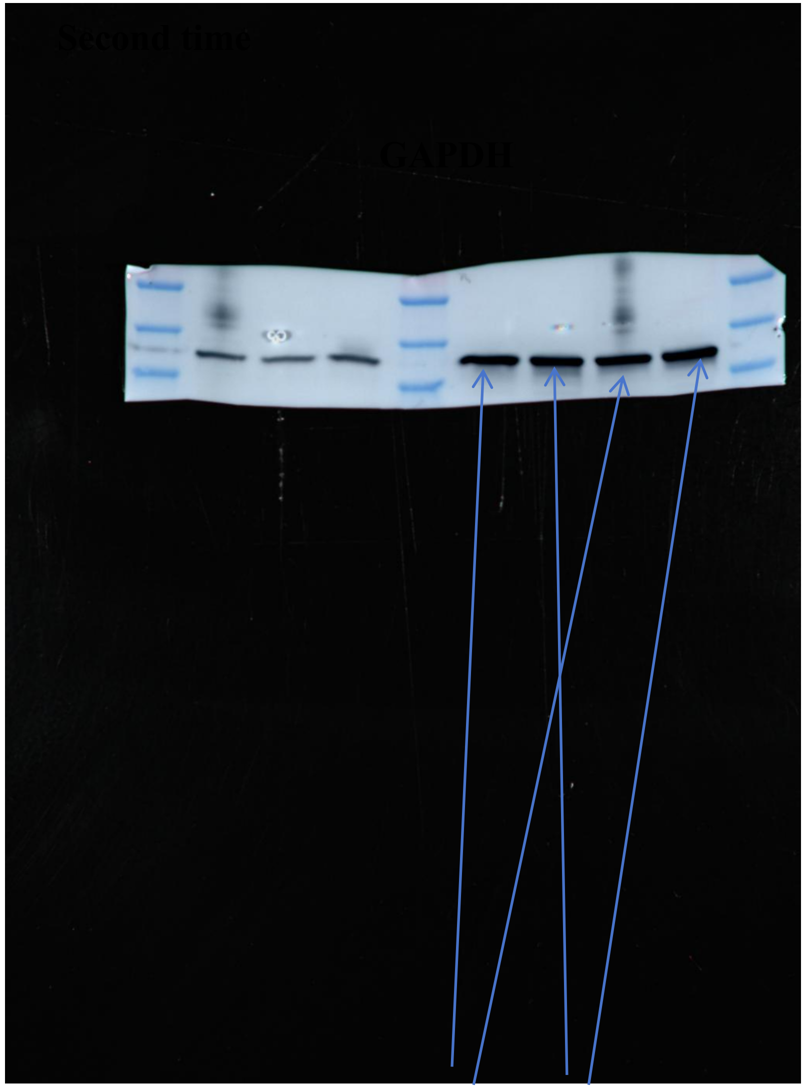

Tex Mock

GSTP1

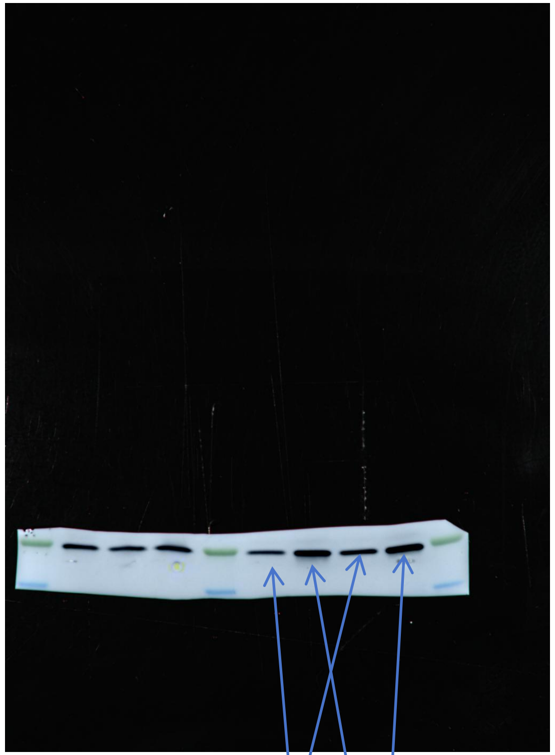

Tex Mock

Together

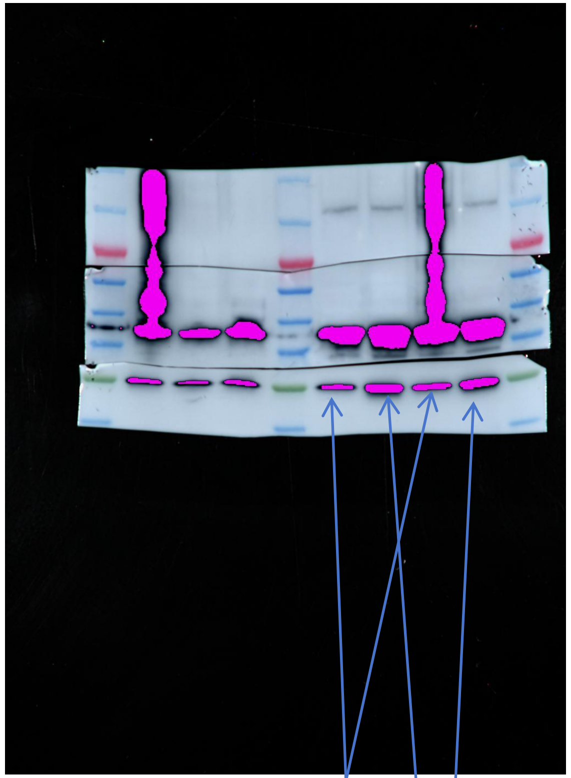

Tex Mock

Third time

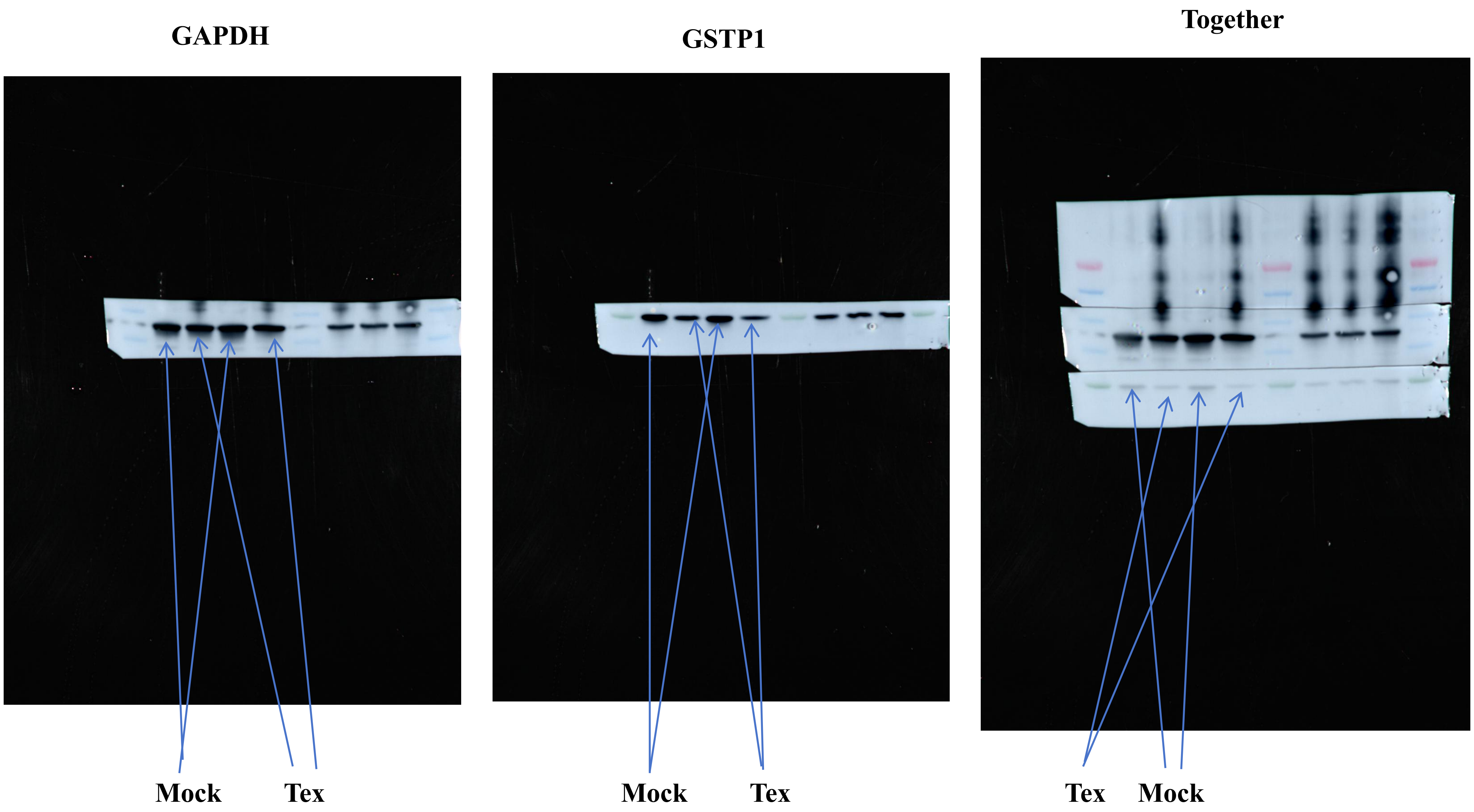

Supplement: Supplementary file 1 [file DataSheet1.zip › Raw date/Figure2/D/Validation of T cell exhaustion model (WB).pdf]
