# Supplementary material for: GSTP1 improves CAR-T cell proliferation and cytotoxicity to combat lymphoma
Source: Front Immunol. 2025 Sep 26;16:1665407. doi: 10.3389/fimmu.2025.1665407 (PMC12511145; doi:10.3389/fimmu.2025.1665407)
Supplement: Supplementary file 1 [file DataSheet1.zip › Raw date/Figure5/A/IL-2 group/GSTP1 enhances the proliferation and cytotoxicity of CAR-T cells to combat lymphoma.docx]

Number of words: 3318. Figure: 1-7

**GSTP1 enhances the proliferation and cytotoxicity of CAR-T cells to combat lymphoma**

Guangsong Xu^1†^, Jiani Wang^1†^, Yuliang Qu^1†^, Jing Ning^2^, ,Yanting Zhang^4^, Guangxian Xu^3^,Yunxia Shi^1^, Ying Li^1^, Le Guo^1*^, Xuebo Han^1*^, Hongxia Wang^1*^.

^1^School of Medical Laboratory, Ningxia Medical University, Yinchuan, Ningxia, China

^2^Hematology department, General Hospital of Ningxia Medical University, Yinchuan, Ningxia, China

^3^Medical Technology School， Guangdong Medical University, Dongguan, Guangdong, China

^4^Gastroenterology department, General Hospital of Ningxia Medical University, Yinchuan, Ningxia, China

*** Correspondence:** Le Guo, [guoletian1982@163.com;](mailto:guoletian1982@163.com;) Xuebo [Han, 372140248@qq.com;](mailto:Han,372140248@qq.com;) Hongxia Wang, [20221004@nxmu.edu.cn](mailto:20221004@nxmu.edu.cn).

Keywords: CAR-T cell exhaustion, BLIMP1, GSTP1, oxidative stress, ROS.

Abstract

****Introduction****

CAR-T cell exhaustion significantly hampers the efficacy of CAR-T cell therapy. Studies show persistent antigen stimulation in T cells making intracellular reactive oxygen species (ROS) surged. ROS, as mitochondrial metabolites, impair integrity of mitochondrial membrane, promoting T cell exhausted. Glutathione S-transferase Pi-1 (GSTP1), a member of the glutathione S-transferase (GST) family. As a main enzyme in the intracellular clearance of ROS, over-expressing GSTP1 may potentially enhance the anti-tumor capability of CAR-T cells.

****Methods****

We analyzed correlations between GSTP1 and T cell exhaustion-related genes using online tool TIMER, collected PBMCs from hematological malignancy patients (*n*=61) and healthy donors (*n*=45), to test GSTP1, BLIMP1, and PD-1 expression via qRT-PCR. Building T cell exhaustion model to test GSTP1 expression by qRT-PCR and WB. Using dual-luciferase assay and ChIP-qPCR to identify transcription factor BLIMP1 negatively regulating promoter GSTP1. CD19 CAR-T, GSTP1 CAR-T and shGSTP1CAR-T cells were generated, and to evaluate their anti-tumor capability.

**Results**

The expression of GSTP1 was down-regulated when BLIMP1 and PD-1 up-regulated in cancer patients’ PBMCs and *in vitro* T cell exhaustion model. Meanwhile, ROS of T cell exhaustion model was increased. Mechanistically, BLIMP1 transcription factor negatively regulated GSTP1 promoter activity. Based above findings, we engineered GSTP1 CAR-T cells, which exhibited enhancing functionality, including GSTP1 CAR-T increased TEMRA population, improved proliferation, cytotoxicity, elevated antioxidant capacity, increased secretion of IL-2 and IFN-γ, reduced immune checkpoint expression, and decreased apoptosis. *In vivo*, we demonstrated that the residual levels of GSTP1 CAR-T cells were higher than CD19 CAR-T cells and shGSTP1 CAR-T cells, indicating that GSTP1 CAR-T cells have good anti-tumour ability.

****Conclusion****

Collectively, we validated BLIMP1 directly suppressing GSTP1 transcription, over-expressing GSTP1 enhanced CAR-T cell anti-tumor capacity by maintaining redox homeostasis, offering a novel therapeutic strategy to improve CAR-T cell immunotherapy

# Introduction

CAR-T cell immunotherapy has revolutionized the treatment of hematologic malignancies, offering durable complete remissions and high objective response rates for multiply treated refractory patients (1, 2). However, high relapse rates still occur in patients with hematologic tumors after accepting CAR-T therapy. CAR-T cell exhaustion, a major hurdle in cancer immunotherapy, is the main reason for reducing the cure rate and hampers anti-tumor efficacy (3). Various strategies were made to improve CAR-T exhaustion from optimizing CAR-T cell structure and culture systems, combating with tumor microenvironment, and to target epigenetic and transcriptomic regulation (4).

Studies indicated that sustained antigen stimulation increases intracellular ROS in T cells, damaging ATP-coupled oxidative phosphorylation. This bioenergetic impairment limits nucleotide triphosphate synthesis, blocking proliferation and affecting T cell self-renewal (5). Continuous antigen stimulation also disrupted T cell function, marked by elevated TIM-3 and PD-1 expression (6). ROS, including superoxide anions, hydrogen peroxide, hydroxyl radicals, ozone, and singlet oxygen (7). Physiologically, ROS derive from mitochondrial respiration and NADPH oxidases act as signaling molecules in T cell activation, proliferation, and development. However, under hypoxia or stress, ROS production surges, damages transcription factors, proteins, lipids, and DNA (8). GSTP1 does not allow ROS to run rampant, instead, it maintains the redox balance of ROS, preventing oxidative stress-induced damage (9).

Studies reported that GSTP1 promoted tumor cell proliferation (10), but its role in T cells remains poorly understood, especially when T cells are stimulated by antigens. Reduced expression of GSTP1 leading to increase mitochondrial ROS levels, weaken cellular function, while over-expression of GSTP1 decrease mitochondrial ROS and restore cellular function (11). To maintain redox balance, effective antioxidant mechanisms involving GSH, NADPH, and redox-regulating proteins like with electrophilic ROS (such as lipid peroxides and 4-hydroxynonenal) (12). GSH, a key endogenous antioxidant, is the primary ROS scavenging pathway in cells. GSTP1 catalyzes the conjugation of glutathione (GSH) with electrophilic ROS to form non-toxic GSH conjugates, which are subsequently exported out of the cell, effectively mitigating ROS induced oxidative damage to cellular components (9).

Our study demonstrated that GSTP1 was depressed in cancer patients’ PBMC and T cell exhaustion model. After over-expressing GSTP1 in CAR-T cells, multiple functions were improved, including: terminal effector memory T cell population was increased, immune checkpoint expression was reduced, apoptosis was improved, proliferative capacity was enhanced. Additionally, GSTP1 over-expressed CAR-T cells augmented cytotoxicity, elevated secretion of IL-2 and IFN-γ, promoted antioxidant capacity. These findings were further validated in mouse lymphoma model, where GSTP1 CAR-T cells significantly improved survival rate of mice. These results suggested that modifying GSTP1 was a promising therapeutic strategy to counteract CAR-T cell exhaustion and improve anti-tumor efficacy.

# Materials and Methods

## Clinical samples

Tumour patient samples (*n*=61) and healthy donor samples（*n*=45) were from clinic. Peripheral blood mononuclear cells (PBMCs) were isolated by density gradient centrifugation with Ficoll separation medium. Total RNA was extracted from PBMCs using TRIzol (Invitrogen), and cDNA was synthesized via reverse transcription, qRT-PCR was performed to detect the expression levels of GSTP1, PD-1, BLIMP1, and GAPDH. Primer sequences were as follow:

GSTP1:Forward, ACTATGAGGCGGGCAAGGATG; Reverse, GCAGCAAGTCCAGCAGGTTG. PD-1:Forward,ACTGCCGCTTCCGTGTCAC;Reverse,AGGTAGGTGCCGCTGTCATTG.BLIMP1:

Forward,TCACAGCAGCCCTGGGAATAC;Reverse,GCGTTCAAGTAAGCGTAGGAGTC.GAPDH:Forward,CAGGAGGCATTGCTGATGAT;Reverse, GAAGGCTGGGGCTCATTT.

All primers were human-specific. This study was approved by the institutional ethics review of The General Hospital of Ningxia Medical University (approval number: KYLL-2025-1370). The study was performed in accordance with the guidelines of the Declaration of Helsinki and relevant regulations. Verbal informed consent was obtained from all participants.

## Isolation of primary CD3^+^T cell

Peripheral blood from healthy donors was subjected to density gradient centrifugation using lymphocyte isolation medium (Solarbio, China) to isolate peripheral blood mononuclear cells (PBMCs). After washing with PBS twice, CD3^+^ T cells were purified by MS Columns (meitenyibiotec, Germany) and CD3^+^ magnetic beads (Meitenyibiotec, Germany). CD3^+^T cells was activated with CD3 (2μg/mL) and CD28 (2μg/mL) (Biogems, American), they coated a day before. Then, the activated CD3^+^ T cells were cultured with complete T cell culture medium (LONZA) and after expanding for 48h before lentiviral transduction.

## Construction of T cell exhaustion model

Firstly, primary CD3^+^T cell was isolated and activated by CD3 (2μg/mL) and CD28(2μg/mL) twice. Then, CD3^+^T cells were stimulated every two days with CD3 (4μg/mL) and CD28 (4 μg/mL). T cells condition were monitored to prevent apoptosis from over-stimulation. On 10th day, cells were collected to detect PD-1 expression (absin, China) and ROS levels (Solarbio, China) via flow cytometry, and whole proteins of T cell were extracted to assess GSTP1 protein expression.

## Transduction of 293T cell

Firstly, lentivirus (GENE, China) carrying the target gene was used to transfect 293T cells. The 293T cells were cultured in DMEM high glucose complete medium until they reached optimal condition. Then, 293T cells were inoculated in a 24 wells culture plate. Lentiviral transduction was performed at an MOI=5 (adjusted as needed based on viral titer). RNA and protein were extracted on day 7 post transduction for subsequent experiments.

## Construction of CAR-T cell

T cells were activated for 48h, then, they were transduced with lentiviral particles encoding CAR (GENE, China). Retronectin (3.6μg/mL) were coated a day before. Each well inoculated 3×10^5^ T cells with 24 wells culture plate and was transduced with lentiviral particles of MOI=6, besides, polybrene( 0.8μg/mL) was added. After centrifugation at 1000g for 2 h, T cells were cultured for 7 days, positive CAR-T cell was measured by flow cytometry (BD Selesta).

## Knock down of shRNA

The cells were collected and extracted into RNA with TRIzol (Invitrogen), the RNA sample reversed transcribe into cDNA (Thermo), and performing q-PCR (SYBR Green) to check the knock down efficiency of shGSTP1#1, shGSTP1#2, and shGSTP1#3. GSTP1 primer sequence: Forward, ACTATGAGGCGGGCAAGGATG; Reverse, GCAGCAAGTCCAGCAGGTTG. Target Sequence：shGSTP1#1,CGCTGACTACAACCTGCTGGA;shGSTP1#2,CCTCACCCTGTACCAG

TCCAA;shGSTP1#3, ACTCAAAGCCTCCTGCCTATA.

## Western blotting assay

Firstly, Cells were collected and extracted into total protein (KeyGEN Bio TECH). Making a BCA standard curve (KeyGEN Bio TECH) to determine protein’s concentration. Then, to perform Western blot assay. Load 20μg of protein samples with 6×loading buffer (LABELAB) was **Electrophoresed** using SDS-PAGE (LabLEAD, China) at 150V for 80min, using 180kDa marker (LabLEAD, China) as a reference. Protein was transfered to PVDF membrane (Vazyme, China) at 400mA for 30 min. To block the membrane with 5% skim milk for 90min at room temperature and then to wash with 1×TBST (SEVEN) for 30 min, to incubate overnight at 4°C with human anti-rabbit GAPDH (CST), human anti-rabbit GSTP1 (CST), and human anti-rabbit BLIMP1 (ProteinTech, China), all antibodies were diluted in 5% skim milk according to ratio1:1000. Next day, wash with 1×TBST for 30 min, then incubate with anti-rabbit HRP(SEVEN) were diluted in 5% skim milk according to ratio 1:10000, for 1h at room temperature. After another 30 min to wash with 1×TBST, using chemiluminescent substrate (NCM Biotech, China) to visualize bands on the Amersham Imager 680 instrument.

## ChIP-qPCR assay

293T and T cells were expanded for the ChIP-qPCR experiment. It mainly followed the Enzymatic Chromatin IP Kit (agarose beads) protocol (CST). The main steps were as follow:1. Cell culture cross-linking and sample preparation. 2. Nuclei preparation and chromatin digestion. 3. Analysis of chromatin digestion and concentration. 4. Chromatin immunoprecipitation (ChIP-grade BLIMP1 antidoby, proteintech, China). 5. Elution of chromatin from antibody/protein G agarose beads and reversal of cross-links. 6. DNA purification using spin columns. 7. Quantification of DNA by qPCR to evaluate the ChIP enrichment efficiency. (CHIP-grade GSTP1 primer sequences: Forward, CTGCTGTCTGTTTACCTAGG, Reverse, CCTGGAGTCCCCGGAGTC) Then subject the amplified PCR products to agarose gel electrophoresis, 100bp DNA ladder maker was used as a reference. To run the gel in 1×TAE buffer at 150V for 30min, then image the gel with an imaging system.

## Cell proliferation assay

Firstly, Daudi cells pretreated with mitomycin C(10μg/mL) and K562 cells pretreated with mitomycin C(30μg/mL) (MCE) for 12h. Then, to stain CAR-T cells with 5μM Cell Trace Far Red reagent (Thermo) in 25min at 37°C from light. CAR-T cells and Daudi or K562 cells were co-cultured respctively at an effector to target ratio of 5:1 in T cell complete culture medium (LOZONA) for 5 days. Finally, CAR-T cells were collected and washed once with PBS, resuspend in 500μL PBS, and analyzed via flow cytometry.

## Flow cytometry analysis

At 7th day post expansion of CAR-T cells, CAR-T cells were collected and resuspended in PBS, making the cell density keep in 1×10⁶ per mL. For memory phenotype test, 2×10^5^ of cells per sample and antibodies were incubated. Anti-human CD3, APC (5μL, Invitrogen); Anti-human CD4, RPA-T4-APC (5μL, Thermo); Anti-human CD8, Per-CP(5μL, BioLegend); Anti-human BV421, CD197 (CCR7) (5μL, Thermo); Anti-human CD45RA, PE (5μL, BioLegend); Mouse anti-Human CD279 (PD-1) (5μL, Absin, China); and BV-421 Mouse anti-human CD197 (5μL,BD). Samples were incubated at 4°C in the dark, then add 500μL PBS to stop incubation. 1500rpmin, they were centrifuged, to discard supernatant and resuspend in 500μL PBS, and then to analyze with a BD Selesta flow cytometer. For ROS test (Solarbio,China), to count cells density at 1×10⁶ per mL, to add 1μL Dihydroethidium(DHE), andthey were incubated at 37°C in the dark for 30min, adding 500μL PBS to stop the incubation, 1500rpmin, centrifuged, supernatant was discarded and re-suspended in 500μL PBS, and to analyze with a BD Selesta flow cytometer. For apoptosis test (absin, China), to collect 3x10^5^cells, adding 500μL of solution with 10μL 7-AAD and 5μL Annexin V to incubated at room temperature in the dark for 5min, then to analyze with a BD C6 Plus flow cytometer. For cell cycle test (absin, China), 1×10⁶ CAR-T cells were fixed in 1mL of 75% ethanol at 4°C for over 2h. Adding 500μL PBS to stop fixation, it was centrifuged, to discard supernatant, wash with 500μL PBS, centrifuged again, to discard PBS, and resuspend in 500μL staining buffer with 25μL PI and 10μL RNase. It was incubated at 37°C in the dark for 30min and then to analyze with a BD C6 Plus flow cytometer.

## LDH cytotoxicity assay

Using CytoTOX 96 Non-Radioactive Cytotoxicity Kit measured cytotoxicity of CAR-T. Briefly,

to abide following steps: Co-cultured CAR-T cells with Daudi or K562 cells at E:T ratios of 1:1,5:1,

10:1, and 20:1 respectively in a 96 wells plate. After 6h, supernatants were collected and added 50μL

substrate and 50μL stop solution. 3. To measured absorbance at 450nm using a Thermo Scientific-

microplate reader. 4. Cytotoxicity percentage was calculated with the formula:

Cytotoxicity (%) = [(Experimental OD-Spontaneous OD)/(Maximum OD-Spontaneous OD)]

×100%

## ELISA

Firstly, to co-culture CAR-T cells with Daudi and K562 cells respectively at E:T ratio of 10:1 in RPMI 1640 complete medium for 24h (no adding additional cytokines). Then, to collect the supernatant and measure content of IL-2 and IFN-γ according to the instructions (absin, China)

## GSSG, GSH test

For GSSG, GSH test based on instructions from Jiancheng Institute of Biotechnology (Nanjing, China). The main steps were as follow: 1. Cell Lysed with sonicator(GD-X40 sonicator) in 100W, (5s on/5s off for 10min). 2. Centrifuged at 3500rpm for 10min at 4°C. Collecting the supernatant to measure absorbancy (A) at 450nm by a Thermo Scientific microplate reader. Results were caculated using the formulas:

GSSG(μmol/L) = [GSSG_test_(A_2_-A_1_)]/[GSSG_stadard_(A_2_-A_1_)]×C_GSSG(standard)_ × dilution fold

GSH(μmol/L) = (A_test_-A_blank_)/(A_stadard_-A_blank_) × C_stadard_ × dilution fold

## Tumour model *in vivo*

4-6 weeks of female NCG mice ( Jicui Biology, China) were fed for 7 days to adapt the environment, then， they were injected with 1×10⁶ Daudi-luc (This cell line express firefly luciferase.) cells via tail vein. 7 days later, 1.5×10⁷ CAR-T cells were injected via tail vein. At various time points, *in vivo* imaging was performed using an IVIS. Mice were anesthetized with 2% isoflurane in oxygen, and 200μL of D-luciferin potassium salt (Promega) was injected by the enterocoelia per mouse. Bioluminescent imaging was done 5 minutes later. The survival of mice were recorded during experiment. Euthanasia was performed if hair loss or rapid weight loss occurred to the mice. For CAR-T cell detection in peripheral blood of mice, to collect mouse orbital blood into heparin-coated tubes, red blood cell lysis buffer was added to the tube (Solarbio, China), and resuspend the white blood cells in PBS to detect CAR-T cells by flow cytometry. For CAR-T cell detection in spleen of mice, the mice was anesthetized, to remove the spleen, and it was ground by a pestle and grinding fluid was filtered through a 0.77µm filter. Performing density gradient centrifugation with Ficoll separation medium to collect the white membrane layer, and to detect CAR-T cells via flow cytometry.

Ethics approval and consent to participate: All the animal experimental procedures were approved by Ningxia Medical University Medical Institutional Animal Care and Use Committee (Approval number: IACUC-NYLAC-2024-245). The permitted study period on the protocol was from December-2024 to April-2025.

## Statistical Analysis

All statistical analyses were performed using SPSS 26.0 and graphs were created using GraphPad Prism version 9.0. The unpaired two tailed t-test was used for comparisons between two groups. For comparisons involving three or more groups, one-way analysis of variance (ANOVA) with multiple comparisons was applied. Statistical significance was defined as **p* < 0.05, ***p* < 0.01, ****p*< 0.001, *****P*<0.0001 and ns (not significant) is *p* > 0.05. All data are presented as the mean ± SD from three independent replicate experiments.

# Results

## GSTP1 was down-regulated in exhausted T cell.

In order to clarify the role of GSTP1 in exhausted T cells, We firstly analyzed the correlation of GSTP1 and genes related to T cell exhaustion using online tool TIMER. The results showed that GSTP1 was negatively correlated with BLIMP1, HAVCR2, IRF4, NR4A2, PD-1, NR4A3, CTLA-4, SOX4, TOX, TIGIT, ID3 and BATF（Figure 1A）. Above those candidates, we chose BLIMP1(B-lymphocyte Maturation Protein 1) for further research in consideration of its function in T cell exhaustion. We collected Peripheral blood mononuclear cells (PBMC) from patients with hematological malignancies and analyzed the expression level of GSTP1. We found that GSTP1 was down-regulated significantly in patients comparing to healthy donors, while both PD-1 and BLIMP1 that were significantly elevated in patients (Figure 1B). These findings suggested that low expression of GSTP1 was associated with potential mechanisms of CAR-T cells exhausted.

To verify this hypothesis, we established an *in vitro* exhaustion model of T cells, specifically, the activated CD3^+^T cells were stimulated with anti-human CD3/CD28 every two days for a total of 10 days (Figure 2A). On day 10, T cells were collected and the expression of PD-1 was analyzed to evaluate T cell exhaustion. The results showed that the expression of PD-1 in the stimulated group was significantly increased, confirming the successful establishment of the T cell exhaustion model (Figure 2B). Afterwards, the levels of ROS increased significantly (Figure 2C), and accordingly, GSTP1 was markedly decreased in the exhausted group (Figure 2D). Besides, we found that BLIMP1 was also highly expressed in exhausted T cells in accordance with the results of clinical samples test. BLIMP1, is a key transcriptional regulator that critically controls immune cell differentiation and function, playing multifaceted and essential roles in T cell development, activation, and exhaustion processes(13, 14). We speculated that BLIMP1 may inhibit the expression of GSTP1 at the transcriptional level. Next, shBLIMP1 was transduced to T cells, and the expression of GSTP1 gene was up-regulated (Figure 2E). Meanwhile, dual-luciferase assay was further recruited to verify the relationship between BLIMP1 and GSTP1. The results indicated that BLIMP1 depressed the activity of promoter GSTP1 (Figure 2F). Based on the analysis results from the JASPAR database, we supposed that the transcription factor BLIMP1 may bind to promoter GSTP1.To verify this conjecture, we employed ChIP-qPCR to detect the binding site of BLIMP1 with GSTP1 and found that the binding site was located at the promoter upstream region from -1788 to -1802 bp (Figure 2G , H). The results uncovered BLIMP1 was significantly bound the GSTP1 promoter and displayed GSTP1 was related to exhaustion in T cells, which was negatively mediated by BLIMP1.

## Workflow of GSTP1 CAR-T cell was generated.

To elucidate the role of GSTP1 in CAR-T cell, we generated three distinct CD19 targeted CAR-T cell variants: CD19 CAR-T cells as control group, GSTP1 over-expressing CAR-T cells (GSTP1 CAR-T), and GSTP1 knock down CAR-T cells (shGSTP1 CAR-T) (Figure 3A). For shGSTP1 CAR-T construction, we first validated shRNA enfficiency in 293T cells and T cells respectively. The results showed that the shGSTP1#2 significantly down-regulated the expression of GSTP1 with approximately 60%, which was sufficient for the following experiments (Figure 3B, C). The pictures showed high transduction rate and positive percentage of CAR-T cells were up to approximately 60% (Figure 3D). Meanwhile, GSTP1 over-expressing was also observed at mRNA and protein levels (Figure 3E). These results manifested that we successfully constructed CD19, GSTP1 and shGSTP1 CAR-T cells.

## GSTP1 CAR-T cell exhibited memory phenotype advantage.

Considering the memory population was reduced in the exhausted T cells (3), we explored the role of GSTP1 in memory phenotype shifting within CAR-T cells. The results showed that the TEMRA population increased in CD4^+^GSTP1 CAR-T cells and CD8^+^GSTP1 CAR-T cells, despite the lack of significant statistical differences (Figure 4A). TEMRA is responsible for acute immune reaction upon target cell. This phenomenon suggested that GSTP1 endowed an anti-tumor advantage to CAR-T cells. We also examined the expression of immune inhibitory checkpoint molecules, PD-1 and CTLA-4 of GSTP1 CAR-T. The results showed that the expression of PD-1 slightly decreased in GSTP1 CAR-T group, while their expression significantly increased in shGSTP1 CAR-T group (Figure 4B,C). These results preliminary illustrated that over-expressing GSTP1 may enhance the anti-tumor ability of CAR-T cells by conferring a memory phenotype advantage.

## GSTP1 augmented the proliferation capacity of CAR-T cell.

To determine the impact of GSTP1 on proliferative capacity of CAR-T cells, we co-cultured Daudi/K562 cells and CAR-T cells to investigate antigen dependent proliferation ability of CAR-T cells. It was found that the proliferative level of GSTP1 CAR-T cells was significantly improved after stimulated by Daudi cells. As expected, when CAR-T cells stimulated by K562 cells, the proliferative ability of the CAR-T cells were disappeared due to lack of CD19 antigen on K562 cells (Figure 5A). Simultaneously, we found that after 7 days of amplification, we counted the numbers of cells in triple groups and found higher proliferative capacity in GSTP1 CAR-T group rather than in CD19 CAR-T group. However, this advantage was abolished once GSTP1 silenced (Figure 5B ). Additionally, we examined the cell cycle of different CAR-T cells, the results revealed that the proportion of the G_0_/G_1_ phase was reduced and the rise of S and G_2_/M phases in GSTP1 CAR-T group, this result was consistent with the phenomenon of enhanced proliferation ability in GSTP1 CAR-T. However, the S phase was lessened after GSTP1 knocked down, this result explained why the proliferative capability was slower in shGSTP1 CAR-T group (Figure 5C). What’s more, we also found apoptotic cells significantly increased in shGSTP1 CAR-T group, the number of apoptotic cells was least in GSTP1 CAR-T group (Figure 5D). The above results interpreted that GSTP1 not only enhanced proliferation ability, but also reduced apoptosis levels of CAR-T cells.

## GSTP1 strengthened the *in vitro* anti-lymphoma suppression of CAR-T cell.

Considering GSTP1 may mediates antioxidant to heighten the cytotoxicity of T cell, we firstly measured the ROS levels of CAR-T cells in each group. The results displayed GSTP1 neutralized ROS within CAR-T cell, nevertheless, the level of ROS in shGSTP1 CAR-T group rose remarkably (Figure 6A). Meanwhile, we collected CAR-T cells to test the oxidized glutathione (GSSG) and reduced glutathione (GSH). The outcomes showed GSSG was decreased in GSTP1 CAR-T group comparing to CD19 CAR-T group, but GSH were increased (Figure 6B). GSH and GSSG collectively maintain the redox balance within cells. GSH primarily functions as an antioxidant and provides cellular protection, whereas GSSG is generated under oxidative stress conditions and is subsequently reduced to regenerate GSH, sustaining the cellular redox state(15). Next, we co-cultured CAR-T cells with tumor cells (Daudi or K562) at an effector target ratio of 10:1. After 24 hours of co-culture, ELISA was used to measure the secretion of IFN-γ and IL-2 in different CAR-T cells. The quantity of IFN-γ and IL-2 were amplified in GSTP1 CAR-T cells while the content of IFN-γ and IL-2 in shGSTP1 CAR-T cells was reduced. With exist of K562 cells, GSTP1 CAR-T cell showed deficiency of secretion of IFN-γ and IL-2 (Figure 6C,D). Moreover, lactate dehydrogenase (LDH)-based assays was used to assess the cytotoxicity of diverse CAR-T cells. Results showed GSTP1 CAR-T cells significantly enhanced anti-tumor cytotoxicity to Duadi cells, whereas shGSTP1 CAR-T cells exhibited inferior anti-tumor competence. After co-cultured with K562 cells, there were no difference on cytotoxicity among three types of CAR-T cells (Figure 6E). These results underlined that GSTP1 indeed reinforced the antioxidant capacity of CAR-T cells and strengthening *in vitro* anti-lymphoma effects.

## GSTP1 enhanced the *in vivo* anti-lymphoma ability of CAR-T cell.

To evaluate the anti-tumor efficacy of GSTP1 CAR-T cells *in vivo*, we established a mouse lymphoma model by injecting 1.0×10⁶ Daudi-luc cells via tail vein, 7 days later, 7.5×10⁷ CAR-T cells were infused in the same way (Figure 7A). Tumor load was monitored by IVIS and results showed that the mice treated with shGSTP1 CAR-T cells died in short time, due to high ROS level weaken anti-tumor function of CAR-T cells (Figure 7B, C, D ,E). Dramatically, GSTP1 CAR-T showed extraordinary anti-tumor ability. On 30th day post injection, orbital venous blood was collected to detect residual of CAR-T cells. The results showed abundant CAR-T cells remaining in mice treated with GSTP1 CAR-T cells, whereas the number of CAR-T cells was minimal in mice treated with shGSTP1 CAR-T cells (Figure 7F). On 102th days, the spleens were harvested from two mice and GSTP1 CAR-T were isolated for flow cytometry analysis. The results showed almost 10% of GSTP1 CAR-T cells were still presented (Figure 7G), which contributed to the survival of mice treated by GSTP1 CAR-T cells. The survival rate showed that mice treated with GSTP1 CAR-T cells prolonged survival (Figure 7H). Together, these results expounded that GSTP1 boosted the anti-tumour capability of CAR-T cells *in vivo*, and extended the survival rate of mice.

# Discussion

CAR-T cell immunotherapy is a revolutionary cellular treatment with significant anti-tumor effects. However, CAR-T cell exhaustion limits its efficacy and application(16). Current strategies to address CAR-T exhaustion mainly include optimizing the structure of CAR-T cell, using gene editing to incorporate 4-1BB and CD28 co-stimulatory molecules into the constructs of CAR, to enhance the ability of T cell activated by antigen (17). Epigenetic interventions, most studies focus on attenuating the exhaustion of CAR-T by gene editing to knock down or over-express some transcription factors to get stronger anti-tumour competence (18). Combining with immune checkpoint inhibitors, PD-1 monoclonal antibodies combined with CAR-T cells can prolong T cell’s survival and boost their anti-tumor ability. Similarly, engineering CAR-T cells to secrete PD-1 antibody single chain variable fragments can enhance anti-tumor activity comparably to PD-1 antibody/CAR-T combinations (19). Improving the tumor microenvironment, blocking immunosuppressive metabolic signals, targeting lactate dehydrogenase, or inhibiting amino acid enzymes in the tumor microenvironment to enhance the efficacy of CAR-T cells (20).

Studies have confirmed that T cell receptor (TCR) signaling increase intracellular ROS in activated T cells (21). ROS, know as participator in the TCR signaling pathway, primarily generated in mitochondria, believed to participate in the TCR signaling pathway and were crucial for the production of IL-2 (22). Moderate levels of ROS can act as signaling molecules to regulate the function of mitochondrial (8). For example, ROS can activate transcription factors such as peroxisome proliferator activated receptor gamma coactivator-1alpha, promoting the expression of genes related to mitochondrial biogenesis and increasing mitochondrial numbers to adapt to the demands of cellular energy (23). However, excessive ROS damage mitochondrial’s structure and triggering lipid peroxidation and disrupting integrity and fluidity of membrane, to weak normal mitochondrial’s function (24, 25). Additionally, ROS oxidize mitochondrial proteins, lead the loss of function, such as impairing key proteins(NADH dehydrogenase, cytochrome b-c1 complex) in the electron transport chain, reducing the efficiency of electron transfer, and further increasing ROS production, to form a vicious cycle (26). GSTP1, a member of the glutathione S-transferase family, catalyzes intracellular antioxidant and detoxification reactions by conjugating the thiol group of cysteine (a key component of glutathione) with electrophilic compounds. Studies have shown that chronic antigen stimulation and hypoxia lead to significant ROS accumulation in T cells (27). Elevated ROS levels enhanced phosphorylated tyrosine signaling and Nuclear Factor of Activated T-cells nuclear translocation, driving T cells toward an exhaustion state (28).

GSTP1 plays multiple roles within cells. Research has demonstrated that DDB1 and CUL4 Associated Factor 1 up-regulated the expression of GSTP1, inhibiting the accumulation of ROS and the aging of regulatory T cells. This mechanism highlighted the significance of GSTP1 in maintaining immune homeostasis and delayed the senescence of immune cells (29, 30). In the tumor microenvironment, which was often characterized by hypoxia or glucose deprivation, GSTP1 non-covalently bound to glucose-6-phosphate dehydrogenase, inhibiting its phosphorylation and enhancing pentose phosphate pathway activity and Nicotinamide Adenine Dinucleotide Phosphate (NADPH) production. This process helped tumor cells resist oxidative stress (31, 32). GSTP1 protected cells from ferroptosis through GSH conjugation and selenium-independent GSH peroxidase activity, a mechanism that was independent of the known ferroptosis defense systems such as GPX4, FSP1, and DHODH. SMAD Specific E3 Ubiquitin Protein Ligase 2 promoted the sensitivity of cancer cells to ferroptosis by mediating the ubiquitination and degradation of GSTP1 (33). The high expression of GSTP1 in tumor cells, which leads to treatment resistance, was essentially due to its ability to clear ROS through multiple pathways, alleviating oxidative stress in tumor cells and promoting their proliferation (34). This phenomenon of enhancing cellular vitality, observed in tumor cells. Over-expressing GSTP1 in CAR-T cells to enhance their viability to against tumors may represent a viable strategy.

Recently, our dual-luciferase assay demonstrated that co-transfection of BLIMP1 and GSTP1 plasmids suppressed the activity of promoter GSTP1, while ChIP-qPCR confirmed that the transcription factor BLIMP1 bound promoter GSTP1. Since BLIMP1 is a key transcription factor within the cell (13), it inhibited the transcription of the promoter GSTP1 at the transcriptional level. After transducing T cells with shBLIMP1, the expression of the GSTP1 gene was increased. Our work demonstrated that BLIMP1 indeed bound to GSTP1, and the suppression of GSTP1 was due to the high expression of BLIMP1 to some extent. One main reason for the accelerated proliferation of tumor cells is the increased expression of GSTP1, because the accelerated proliferation leads to the generation of excessive peroxides, which need to be promptly cleared by GSTP1 (35). Accordingly, GSTP1 over-expression in CAR-T cells observed enhancing proliferative capacity, this promoted T cell proliferative capacity enabling rapidly expansion of tumor-specific T cell populations, facilitating tumor cells were eliminated timely (36). Interestingly, GSTP1 over-expression increased the proportion of terminally differentiated effector memory T cells (TEMRA), although central memory T cells (TCM) serve as the primary phenotype for maintaining T cell self-renewal and play a crucial role in mitigating CAR-T cell exhaustion, our findings uncovered that over-expression of GSTP1 did not enhance the TCM population in CAR-T cells. Instead, it promoted the expansion of TEMRA, this population was characterized by having outstanding cytotoxicity to eliminate the tumour (37). The proportion of TCM population was increased in shGSTP1 CAR-T might be due to the inhibition of cell proliferation after GSTP1 was knocked down, causing the T cells to keep in a quiescent state. The S phase was reduced after GSTP1 was knock down in CAR-T cells, this appearance elaborated why the proliferation of shGSTP1 CAR-T cells was arrested (10). However, this inhibition was long-term and does not lead to proliferate even upon re-stimulated by antigen. We tested that GSTP1 over-expression did not significantly down-regulated expression levels of PD-1 and CTLA-4, however, shGSTP1 CAR-T cells exhibited remarkable up-regulation of both PD-1 and CTLA-4. GSTP1 knock down led to intracellular accumulation of diverse oxidative metabolic byproducts. Through assessment of the GSH/GSSG ratio, we effectively monitored the oxidative stress status within CAR-T cells. Typically, an elevated intracellular GSH/GSSG ratio indicated a reduced cellular state, while decreased ratio reflects heightened oxidative stress, maintenance of redox homeostasis is crucial for normal cellular physiological functions (9). Redox imbalance could disrupt ATP synthesis during reductive stress, increased NADH/NAD^+^ ratio shifts cellular metabolism from mitochondrial respiration toward glycolysis, resulting in insufficient energy production (28, 38).

In view of oxidative stress is just one of many factors contributing to T cell exhaustion (5), GSTP1 was likely only partially responsible for mitigating exhaustion. Therefore, a comprehensive evaluation need to determine whether enhancing antioxidant capacity alone can alleviate exhaustion. To further optimize CAR-T cell performance, alternative strategies, such as supplementing culture media with antioxidant compounds, such as N-Acetylcysteine or administering antioxidant drugs *in vivo* may prove more effective. Besides, because of the potential for recurrence after tumor treatment, model validation for recurrence should also be conducted. For mice with high tumor burdens, reducing the dose or using a fractionated dosing approach can diminish the risk of cytokine release syndrome and other toxicities (39, 40). Finally, mitochondria are a primary intracellular source of ROS, given that GSTP1 central role in redox homeostasis, further research is needed to clarify how GSTP1 affects this process in T cell. GSTP1 was highly expressed in tumor cells and promoted glycolysis (12). To investigate the influence of GSTP1 on metabolism, we observed that GSTP1 CAR-T emerged a slight up-regulation of key glycolytic enzymes (GLUT1 and LDHA) , whereas shGSTP1 CAR-T significantly enhanced glycolysis (date not shown). We postulated that the mild increase in glycolytic enzymes in GSTP1 CAR-T cells may result from accelerated proliferation, which demanded higher energy consumption to support biosynthetic (35). In contrast, the pronounced glycolytic up-regulation in shGSTP1 CAR-T cells could be attributed to oxidative stress induced mitochondrial damage ascribe to GSTP1 knocked down. This metabolic shift reflected a compensatory mechanism in which cells increased glucose uptake to sustain ATP production, as impairing mitochondrial function reduced oxidative phosphorylation efficiency (41). In subsequent experiments, we will further explore the role of GSTP1 in T cells from the perspective of protein-protein interactions. Understanding these mechanisms will provide deeper insights into the regulation of T cell metabolism and function. This knowledge aid in generating strategies to optimize CAR-T cell therapy by enhancing their resistance to oxidative stress and improving their therapeutic efficacy.

# Conclusion

In summary, our study revealed that the transcription factor BLIMP1 negatively regulated the expression of promoter GSTP1, which was implicated in the exhaustion of CAR-T cells. Silencing GSTP1 significantly promoted the exhaustion of CAR-T cells, highlighting its critical role in maintained T cell functionality. Conversely, over-expression of GSTP1 not only mitigated oxidative stress but also intensified the anti-tumor efficacy of CAR-T cell. These findings illustrated that modulating GSTP1 expression could serve as a promising strategy to optimize CAR-T cell immunotherapy.

**Abbreviations**

CAR-T: chimeric antigen receptor T cells, GSTP1: Glutathione S-transferase-pi 1, BLIMP1: B-lymphocyte Maturation Protein 1, ROS: reactive oxygen species, PBMCs: Peripheral blood mononuclear cells, LDH: lactate dehydrogenase, qRT-PCR: real-time quantitative PCR, ELISA: enzyme linked immunosorbent assay, WB: Western blotting, PD-1: programmed cell death protein 1, CTLA-4: cytotoxic T-lymphocyte-associated protein 4, scFv: single chain antibody fragment, TCM: Central memory T cell, TEM: Effector memory T cell, Tn: Naïve T cell, TEMRA: terminally differentiated effector memory T cells, GSSG: Glutathione Disulfide, GSH: Reduced Glutathione, IVIS: In Vivo Imaging System, FSP1: Ferroptosis Suppressor Protein 1, TCR: T Cell Receptor, PGC-1α: Peroxisome Proliferator Activated Receptor Gamma Coactivator 1-alpha, GPX4：Glutathione Peroxidase 4, DHODH：Dihydroorotate Dehydrogenase, SMURF2：SMAD Specific E3 Ubiquitin Protein Ligase 2, NADH： Nicotinamide Adenine Dinucleotide, NAD^+^：Nicotinamide Adenine Dinucleotide.

# Data Availability Statement

Further inquiries can be directed to the corresponding author.

# Ethics statement

The animal study was approved by Institutional Animal Care and Use Committee of Ningxia Medical University. The collection of clinical samples was approved by the institutional ethics review of The General Hospital of Ningxia Medical University. These studies was conducted in accordance with the local legislation and institutional requirements.

# Author Contributions

HW: Conceptualization, Funding acquisition, Project administration, Supervision. GX: Data curation, Formal analysis, Investigation, Methodology, Software, Validation, Writing–review & editing. JW: Data curation, Formal analysis, Investigation. YQ:Methodology, Software, Validation, Writing – review & editing. JN: Data curation, Formal analysis, Investigation. GX: Data curation, Methodology, Writing–review & editing. YZ:Data curation, Formal analysis, Investigation. YS:Data curation, Formal analysis, Investigation. YL: Data curation, Formal analysis, Investigation. LG:Conceptualization, Investigation, Writing–review & editing. XH: Conceptualization, Investigation, Writing – review & editing.

# Funding

The author declare this work was supported by the Ningxia Natural Science Foundation (2024AAC03235), Special Talent Startup Project of Ningxia Medical University (XT2023026) and Key Projects of Ningxia Medical University (Open Projects) (XZ2024003).

# Acknowledgments

We thank to all team members for their participation and contributions.


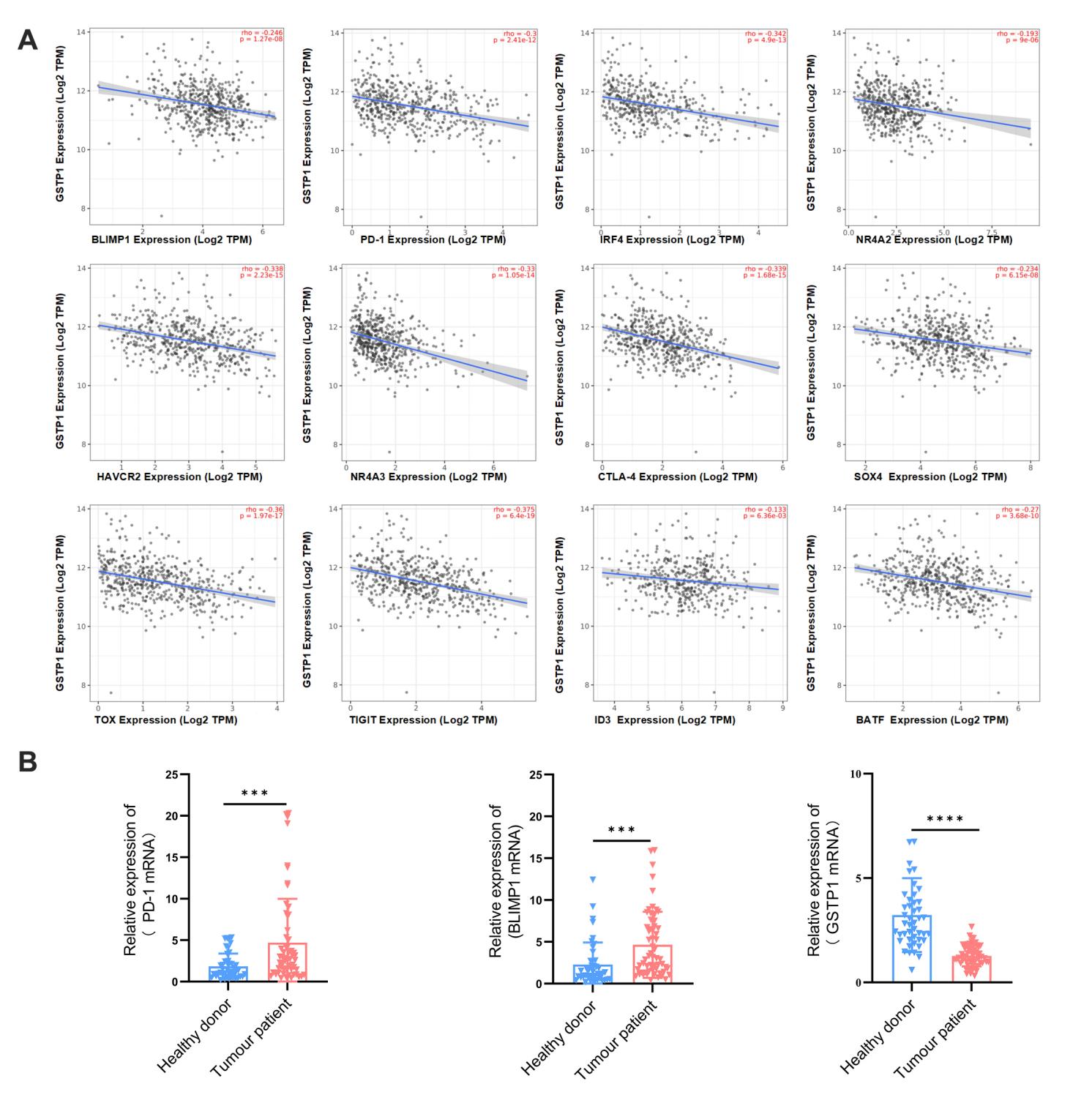


**Figure1:** GSTP1 was down-regulated in the PBMC of cancer patients. A. Using the online tool TIMER, the relationship between GSTP1 and genes associated with T cell exhaustion was analyzed. It was found that GSTP1 was negatively correlated with BLIMP1, HAVCR2, IRF4, NR4A2, PD-1, NR4A3, CTLA-4, SOX4, TOX, TIGIT, ID3 and BATF. B. the RNA of peripheral blood mononuclear cell was extracted from cancer patients(*n*=61) and healthy people (*n*=45), and the mRNA expression levels of GSTP1, PD-1, and BLIMP1 were measured by qRT-PCR with three replicates for each sample. The unpaired two tailed t-test was used for comparisons between two groups. **P* < 0.05, ***P* < 0.01, ****P*< 0.001, *****P*<0.0001 and ns (not significant) is *P* > 0.05.


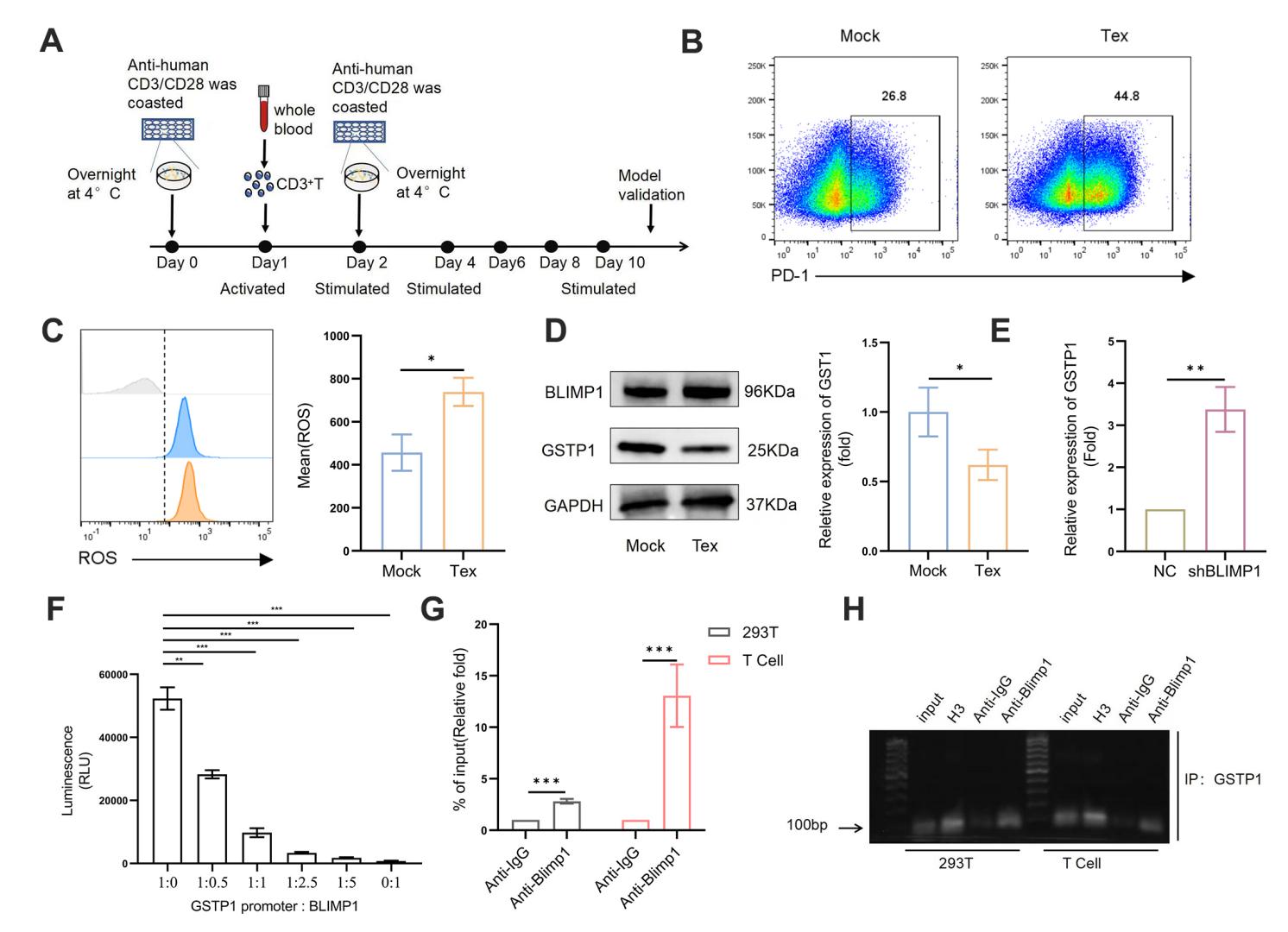


**Figure2:** GSTP1 was down-regulated in T cell exhaustion model. A. CD3^+^T cells isolated from peripheral blood of healthy donors, it were activated using CD3 and CD28. After 24 hours, they were re-activated, then, the Tex group was subsequently stimulated every 2 days using CD3 and CD28. B. On 10th day, T cells were collected to validate exhaustion model, flow cytometry was used to test the expression of PD-1. C. ROS (DHE probe ) was tested by flow cytometry. D. Using Western blotting to test the expression of GSTP1 in T cell exhaustion model. E. shBLIMP1 was used transducted T cell, then the expression of GSTP1was measured by qRT-PCR. F. BLIMP1 and GSTP1 plasmids were co-transfected into 293T cells at different ratios, dual luciferase assay was used to test the activity of promoter GSTP1. G. For ChIP-qPCR assay, after culturing sufficient quantities of 293T and T cells, chromatin immunoprecipitation was performed to verify the transcription factor BLIMP1 binding promoter GSTP1, H3 was positive control, anti-IgG was negative control, anti-BLIMP1 was used as the experimental group. H. Products of ChIP-PCR though nucleic acid electrophoresis was used to further confirm that the transcription factor BLIMP1 bound promoter GSTP1. The unpaired two tailed t-test was used for comparisons between two groups. **P* < 0.05, ***P* < 0.01, ****P*< 0.001,*****P*<0.0001 and ns (not significant) is *P* > 0.05.


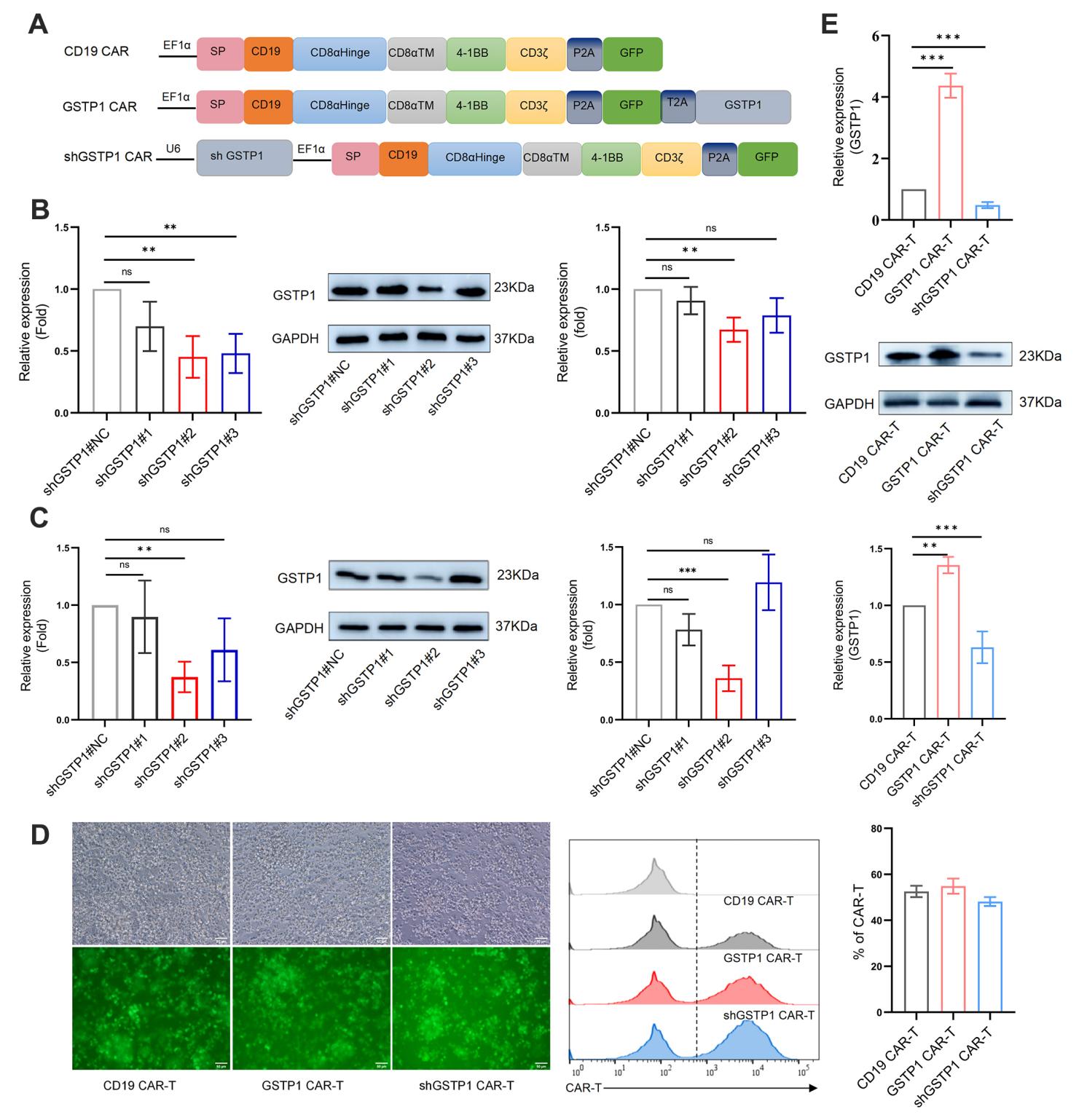


**Figure3:** The CAR-T cells were constructed. A.The structural framework of CD19 CAR, GSTP1 CAR, shGSTP1 CAR. B. Lentivirus-packaged shGSTP1#1, shGSTP1#2, and shGSTP1#3 were transfected into 293T cells to assess knocked down efficiency of GSTP1, from left to right: qRT-PCR validation, WB verification, grayscale value analysis of WB. C. Lentivirus-packaged shGSTP1#1, shGSTP1#2, and shGSTP1#3 were transfected into primary T cells to evaluate knocked down efficiency of GSTP1, from left to right: qRT-PCR validation, WB verification, grayscale value analysis of WB. D. Three kinds of CAR-T cells were constructed successfully to see the expression of GFP using fluorescence imaging, flow cytometry was applied to test the percentage of three kinds of CAR-T. E. The over-expression efficiency of GSTP1 was verified in triple CAR-T cells. From top to bottom: qRT-PCR validation, WB verification, grayscale value analysis of WB ,The experiment was repeated three times independently. The unpaired two tailed t-test was used for comparisons between two groups. **P* < 0.05, ***P* < 0.01, ****P*< 0.001, *****P*<0.0001 and ns (not significant) is *P* > 0.05.


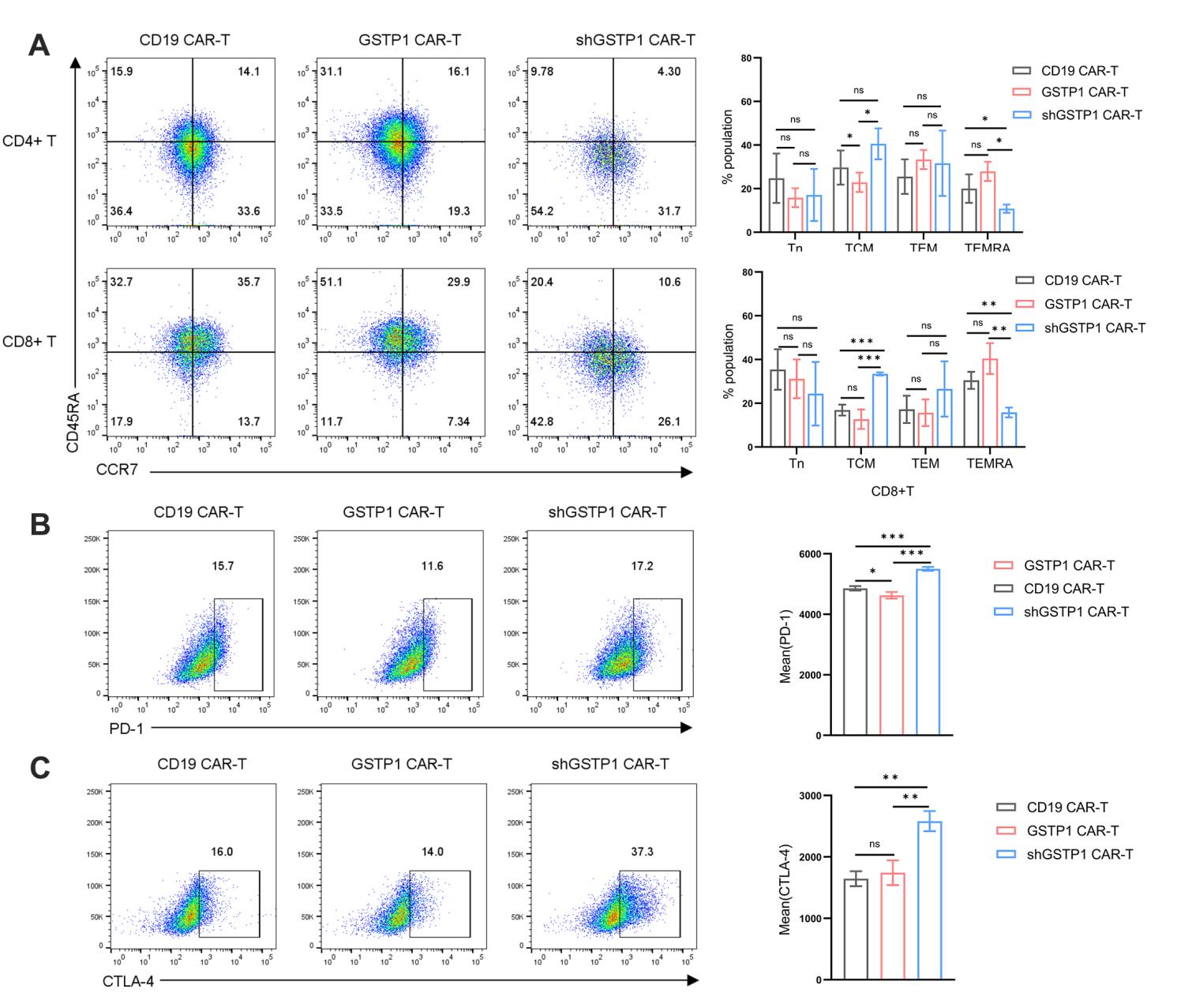


**Figure4:** The proportion of terminal effector memory subset increased in GSTP1 CAR-T cell and immune inhibitory marker increased in shGSTP1 CAR-T cell. A. After 7 days of CAR-T cells were cultured, cells were collected to test markers CCR7 and CD45RA with flow cytometry. The expression of CD45RA and CCR7 was analyzed within the CD4^+^CAR-T and CD8^+^CAR-T cell populations respectively (Tn: CD45RA^+^CCR7^+^; TCM: CD45RA^-^CCR7^+^; TEM: CD45RA^-^CCR7^-^; TEMRA: CD45RA^+^CCR7^-^). B. After 7 days of CAR-T cell expansion, cells were collected and analyzed by flow cytometry to detect expression levels of PD-1. C. After 7 days of CAR-T cell expansion, cells were collected and analyzed by flow cytometry to detect expression levels CTLA-4. The unpaired two tailed t-test was used for comparisons between two groups. **P* < 0.05, ***P* < 0.01, ****P*< 0.001, *****P*<0.0001 and ns (not significant) is *P* > 0.05.


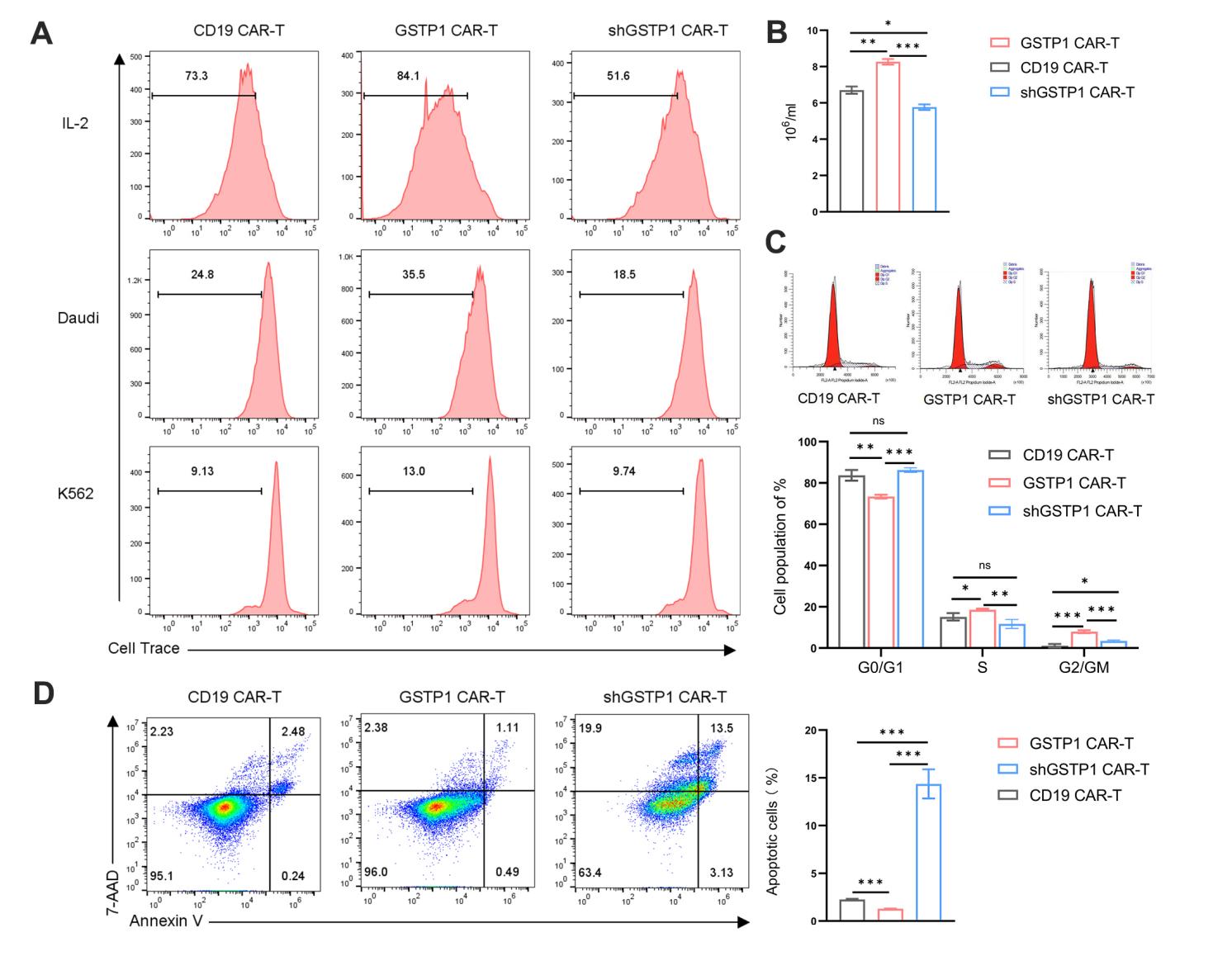


**Figure5:** Proliferation capacity was enhanced and apoptosis improved in GSTP1 CAR-T cell**.** A. CAR-T cells were cultured in complete medium and stained with Cell Trace Far Red proliferation dye, 5 days later, proliferation of CAR-T was assessed by flow cytometry (Top row including IL-2). And Daudi and K562 cells were pretreated with puromycin C for 12 hours and then co-cultured with CAR-T cells and Daudi/K562 respectively at an effector to target (E:T) ratio of 5:1(no adding IL-2), before co-cultured, CAR-T cells were collected and stained with Cell Trace Far Red proliferation dye, after 5 days of co-culture, the proliferative capacity of CAR-T was assessed by flow cytometry. B. CAR-T cells (the number of cells was 4×10^5^ per group at the beginning) were expanded for 7 days in the presence of IL-2, and the number of CAR-T cell were counted using microscope in three independent experiments. C. After 7 days of CAR-T cell expansion, cells were stained with PI (propidium iodide) cell cycle reagent, and changes of cell cycle were examined within the CAR-T cell population. D. After 7 days of CAR-T cell expansion, flow cytometry was performed to assess apoptosis in CAR-T cells. The experiments was repeated three times independently. The unpaired two tailed t-test was used for comparisons between two groups. **P* < 0.05, ***P*< 0.01, ****P*< 0.001, *****P*<0.0001 and ns (not significant) is *P* > 0.05.


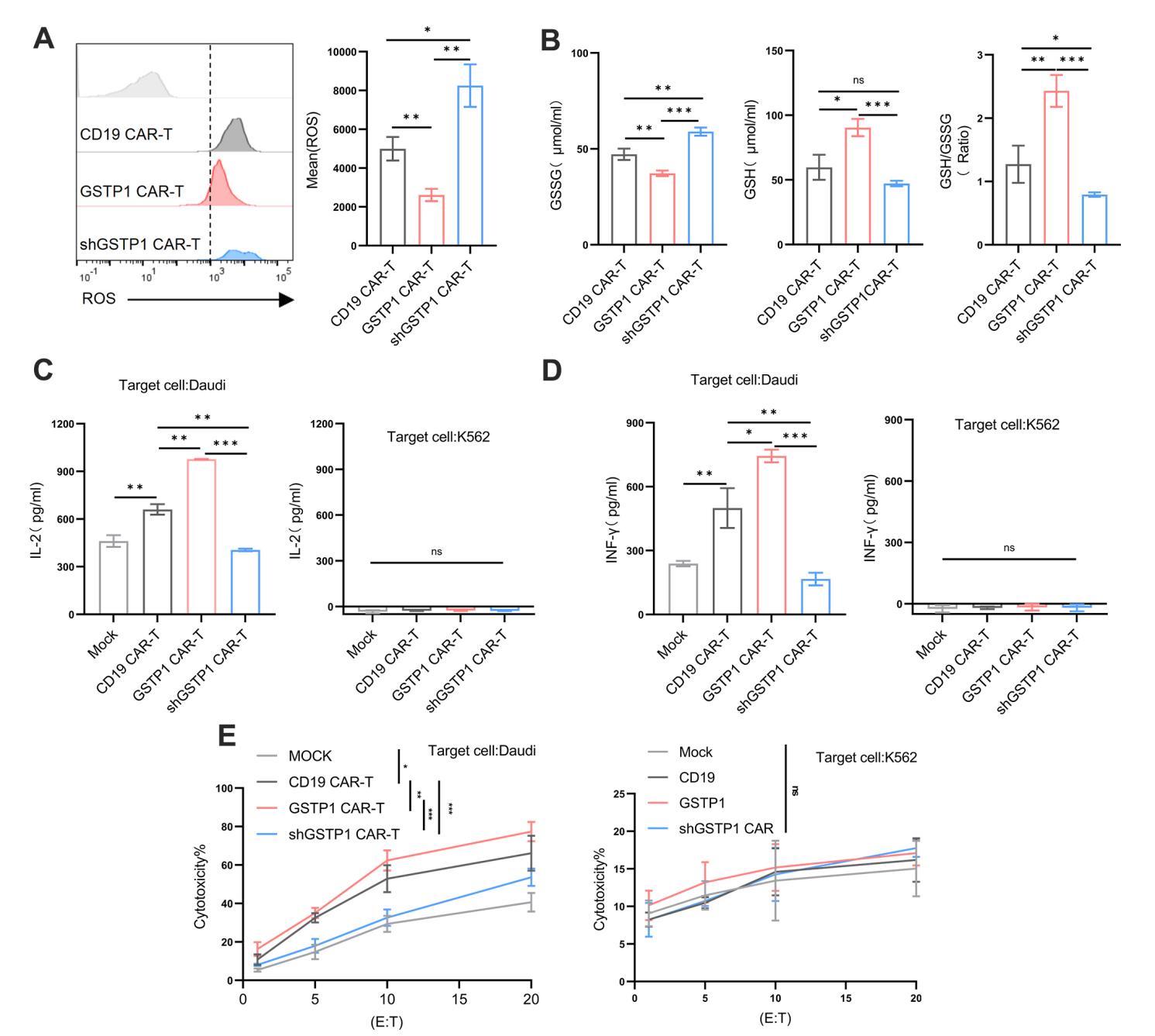


**Figure6:** GSTP1 CAR-T improved cytotoxity, increased secretion of IL-2 and IFN-γ, enhanced anti-oxidative capacity. A. After 7 days of CAR-T cell was expanded, cells were stained with DHE probe and then analyzed by flow cytometry to detect ROS levels in CD19 CAR-T, GSTP1 CAR-T, and shGSTP1 CAR-T cells. B. After 7 days of CAR-T cells expanded, CAR-T cells (5.0×10^6^ cells per group) were collected and lysed using an sonicator. The lysates were centrifuged at 3,500 rpm for 10 minutes, and the supernatants were subjected to colorimetric reaction. OD values were measured at 450nm to calculate GSSG and GSH content. C. CAR-T cells were co-cultured with Daudi/K562 cells at an effector-to-target ratio of 10:1 for 24 hours, then, co-culture supernatants were collected by ELISA to detect IL-2 secretion of CAR-T cells. D. CAR-T cells were co-cultured with Daudi/K562 cells at an effector-to-target ratio of 10:1 for 24 hours, then, supernatants of co-culture were collected using ELISA to detect IFN-γ secretion of CAR-T cells. E. CAR-T cells were co-cultured with Daudi /K562 cells at different effector-to-target ratios (20:1, 10:1, 5:1, 1:1) for 6 hours, then the supernatants of co-culture were collected to assess the cytotoxic capacity of various CAR-T cells by testing the content of LDH. All experiments were performed with three independent replicates.The unpaired two tailed t-test was used for comparisons between two groups, **P*< 0.05, ***P* < 0.01, ****P*< 0.001, *****P*<0.0001 and ns (not significant) is *P* > 0.05.


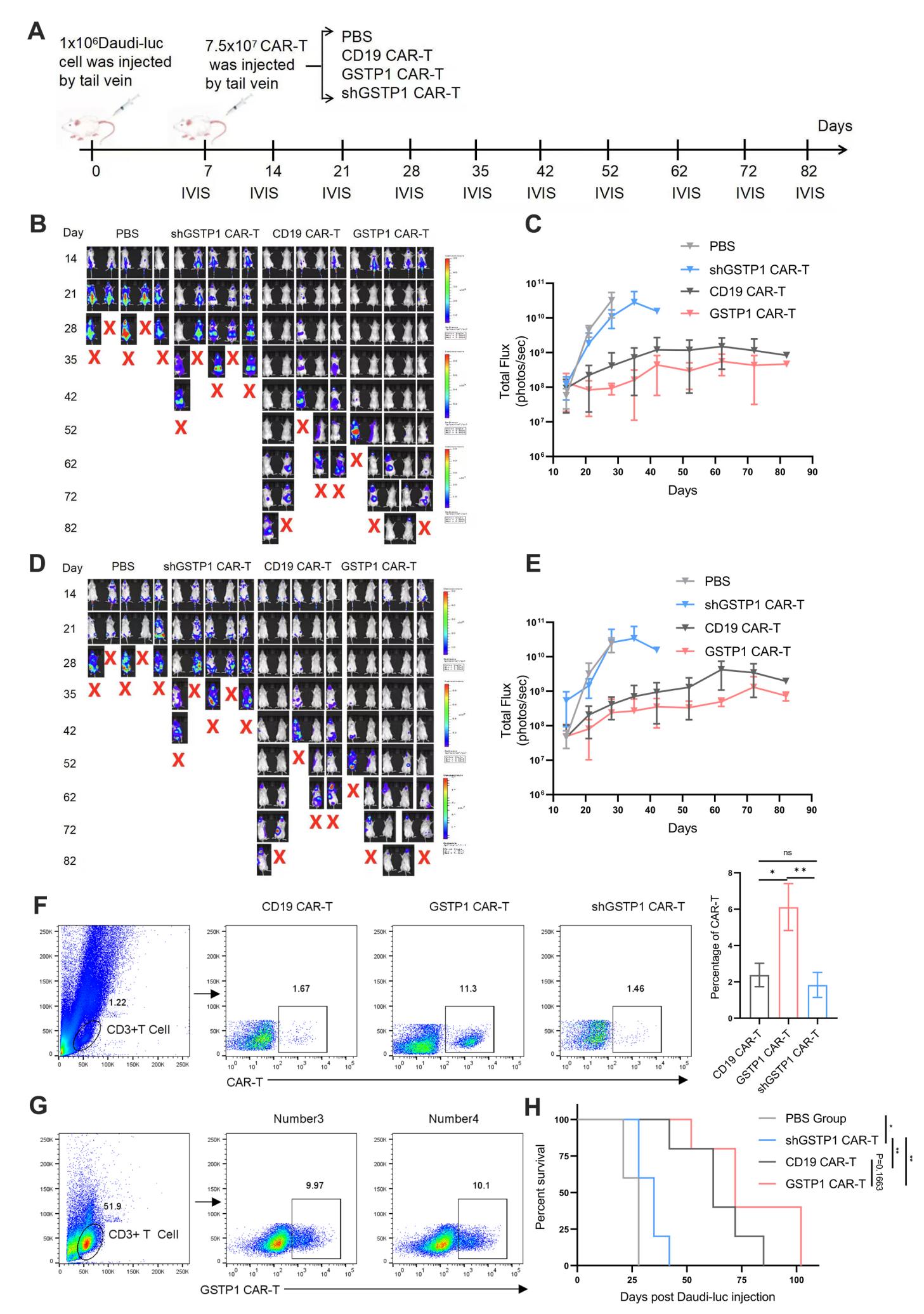


**Figure7:** GSTP1 CAR-T has advantage on anti-tumour capacity *in vivo.* A. Flowchart of NCG mice injected with Daudi-luc cells and CAR-T cells. **B-**C. Fluorescence quantification graphs of the dorsal region in four mice groups, mice were imaged by IVIS at different time point. D-E. Fluorescence quantification graphs of the belly region in four mice groups, mice were imaged by IVIS at different time point. F. On Day 30 after Daudi-luc cell was injected, orbital venous blood of three mice was collected from per group (CD19 CAR-T, GSTP1 CAR-T, shGSTP1 CAR-T group). After red blood cell was lysed, flow cytometry was used to detect the residual of CAR-T cells in each mouse. G. Two mice from the GSTP1 CAR-T group were dissected after anesthetization, then their spleens were harvested, the spleen tissue was ground by a pestle and through a 0.77µm filter to filter, and the cell suspension was subjected to Ficoll density gradient centrifugation, the leukocyte layer was collected, and flow cytometry was performed to assess the residual of CAR-T cell in the spleen. H. Every mouse’s survival was recorded.**P* < 0.05, ***P* < 0.01, ****P*< 0.001, *****P*<0.0001 and ns (not significant) is *P* > 0.05.

# Reference

1. Lu J.Jiang G. The journey of CAR-T therapy in hematological malignancies. *Mol Cancer*. (2022) 21(1). doi: 10.1186/s12943-022-01663-0

2. Gu T, Zhu M, Huang H.Hu Y. Relapse after CAR-T cell therapy in B-cell malignancies: challenges and future approaches. *J Zhejiang Univ Sci B*. (2022) 23(10):793-811. doi: 10.1631/jzus.B2200256

3. Zhu X, Li Q.Zhu X. Mechanisms of CAR T cell exhaustion and current counteraction strategies. *Front Cell Dev Biol*. (2022) 10. doi: 10.3389/fcell.2022.1034257

4. Gumber D.Wang LD. Improving CAR-T immunotherapy: Overcoming the challenges of T cell exhaustion. *eBioMedicine*. (2022) 77. doi: 10.1016/j.ebiom.2022.103941

5. Vardhana SA, Hwee MA, Berisa M, Wells DK, Yost KE, King B, et al. Impaired mitochondrial oxidative phosphorylation limits the self-renewal of T cells exposed to persistent antigen. *Nat Immunol*. (2020) 21(9):1022-33. doi: 10.1038/s41590-020-0725-2

6. Lee KA, Shin KS, Kim GY, Song YC, Bae EA, Kim IK, et al. Characterization of age‐associated exhausted CD8+ T cells defined by increased expression of Tim‐3 and PD‐1. *Aging Cell*. (2016) 15(2):291-300. doi: 10.1111/acel.12435

7. He L, He T, Farrar S, Ji L, Liu T.Ma X. Antioxidants Maintain Cellular Redox Homeostasis by Elimination of Reactive Oxygen Species. *Cell Physiol Biochem*. (2017) 44(2):532-53. doi: 10.1159/000485089

8. Lennicke C.Cochemé HM. Redox metabolism: ROS as specific molecular regulators of cell signaling and function. *Mol Cell*. (2021) 81(18):3691-707. doi: 10.1016/j.molcel.2021.08.018

9. Georgiou-Siafis SK.Tsiftsoglou AS. The Key Role of GSH in Keeping the Redox Balance in Mammalian Cells: Mechanisms and Significance of GSH in Detoxification via Formation of Conjugates. *Antioxidants*. (2023) 12(11). doi: 10.3390/antiox12111953

10. Singh RR, Mohammad J, Orr M.Reindl KM. Glutathione S-Transferase pi-1 Knockdown Reduces Pancreatic Ductal Adenocarcinoma Growth by Activating Oxidative Stress Response Pathways. *Cancers*. (2020) 12(6). doi: 10.3390/cancers12061501

11. Nicole E. Scharping DBR, Ashley V. Menk ,Paolo D. A. Vignali1, B. Rhodes Ford2, Natalie L. Rittenhouse, Ronal Peralta, Yiyang Wang, . Mitochondrial stress induced by continuous stimulation under hypoxia rapidly drives T cell exhaustion. *Nat Immunol*. (2021) 22:205-15. doi: 10.1038/s41590-020-00834-9

12. Ramon I KG, David O'Sullivan, Erika L.Pearce. Caught in the cROSsfire: GSH Controls T Cell Metabolic Reprogramming *Immunity*. (2017) 18(46):525-27. doi: 10.1016/j.immuni.2017.03.022

13. Wang AYL, Aviña AE, Liu Y-Y, Chang Y-C.Kao H-K. Transcription Factor Blimp-1: A Central Regulator of Oxidative Stress and Metabolic Reprogramming in Chronic Inflammatory Diseases. *Antioxidants*. (2025) 14(2). doi: 10.3390/antiox14020183

14. Wu J, Yang K, Cai S, Zhang X, Hu L, Lin F, et al. A p38α-BLIMP1 signalling pathway is essential for plasma cell differentiation. *Nat Commun*. (2022) 13(1). doi: 10.1038/s41467-022-34969-0

15. Klein Geltink RI, O’Sullivan D.Pearce EL. Caught in the cROSsfire: GSH Controls T Cell Metabolic Reprogramming. *Immunity*. (2017) 46(4):525-27. doi: 10.1016/j.immuni.2017.03.022

16. Kasakovski D, Xu L.Li Y. T cell senescence and CAR-T cell exhaustion in hematological malignancies. *J Hematol Oncol*. (2018) 11(1). doi: 10.1186/s13045-018-0629-x

17. Esther Drent RP, Ruud Ruiter, Niels W.C.J. van de Donk, Sonja Zweegman, Huipin Yuan, Joost de Bruijn, Michel Sadelain, Henk M. Lokhorst, Richard W.J. Groen, Tuna Mutis, Maria Themeli,. Combined CD28 and 4–1BB costimulation potentiates affinitytuned Chimeric Antigen Receptor-engineered T cells. *Clin Cancer Res*. (2019) 25(13):4014-25. doi: 10.1158/1078-0432.CCR-18-2559

18. Li X, Zhu T, Wang R, Chen J, Tang L, Huo W, et al. Genetically Programmable Vesicles for Enhancing CAR‐T Therapy against Solid Tumors. *Adv Mater*. (2023) 35(19). doi: 10.1002/adma.202211138

19. Zhou Y, Mu W, Wang C, Zhuo Z, Xin Y, Li H.Wang C. Ray of dawn: Anti-PD-1 immunotherapy enhances the chimeric antigen receptor T-cell therapy in Lymphoma patients. *BMC Cancer*. (2023) 23(1). doi: 10.1186/s12885-023-11536-4

20. Li N, Tang N, Cheng C, Hu T, Wei X, Han W.Wang H. Improving the anti-solid tumor efficacy of CAR-T cells by inhibiting adenosine signaling pathway. *OncoImmunology*. (2020) 9(1). doi: 10.1080/2162402x.2020.1824643

21. Laura A. Sena SL, Amit Jairaman, Murali Prakriya, Teresa Ezponda, David A. Hildeman, Chyung-Ru Wang, Paul T. Schumacker, Jonathan D. Licht, Harris Perlman,. Mitochondria are required for antigen-specific T cell activation through reactive oxygen species signaling. *Immunity*. (2013) 38(2):225-36. doi: 10.1016/j.immuni.2012.10.020

22. Murphy Michael P.Siegel Richard M. Mitochondrial ROS Fire Up T Cell Activation. *Immunity*. (2013) 38(2):201-02. doi: 10.1016/j.immuni.2013.02.005

23. Kirova DG, Judasova K, Vorhauser J, Zerjatke T, Leung JK, Glauche I.Mansfeld J. A ROS-dependent mechanism promotes CDK2 phosphorylation to drive progression through S phase. *Dev Cell*. (2022) 57(14):1712-27.e9. doi: 10.1016/j.devcel.2022.06.008

24. Peng H-Y, Lucavs J, Ballard D, Das JK, Kumar A, Wang L, et al. Metabolic Reprogramming and Reactive Oxygen Species in T Cell Immunity. *Front Immunol*. (2021) 12. doi: 10.3389/fimmu.2021.652687

25. Peoples JN, Saraf A, Ghazal N, Pham TT.Kwong JQ. Mitochondrial dysfunction and oxidative stress in heart disease. *Exp Mol Med*. (2019) 51(12):1-13. doi: 10.1038/s12276-019-0355-7

26. Si X, Shao M, Teng X, Huang Y, Meng Y, Wu L, et al. Mitochondrial isocitrate dehydrogenase impedes CAR T cell function by restraining antioxidant metabolism and histone acetylation. *Cell Metab*. (2024) 36(1):176-92.e10. doi: 10.1016/j.cmet.2023.12.010

27. Scharping NE, Rivadeneira DB, Menk AV, Vignali PDA, Ford BR, Rittenhouse NL, et al. Mitochondrial stress induced by continuous stimulation under hypoxia rapidly drives T cell exhaustion. *Nat Immunol*. (2021) 22(2):205-15. doi: 10.1038/s41590-020-00834-9

28. Wu H, Zhao X, Hochrein SM, Eckstein M, Gubert GF, Knöpper K, et al. Mitochondrial dysfunction promotes the transition of precursor to terminally exhausted T cells through HIF-1α-mediated glycolytic reprogramming. *Nat Commun*. (2023) 14(1). doi: 10.1038/s41467-023-42634-3

29. Zengli Guo GW, Bing Wu, Junnian Zheng, Jenny P.-Y. Wei-Chun Chou, Liang Cheng,Yisong Y,. DCAF1 regulates Treg senescence via the ROS axis during immunological aging. *J Clin Invest*. (2020) 130:5893-908. doi: 10.1172/JCI136466

30. Zheng Y, Xu R, Chen X, Lu Y, Zheng J, Lin Y, et al. Metabolic gatekeepers: harnessing tumor-derived metabolites to optimize T cell-based immunotherapy efficacy in the tumor microenvironment. *Cell Death Dis*. (2024) 15(10). doi: 10.1038/s41419-024-07122-6

31. Wang SQ, Chen JJ, Jiang Y, Lei ZN, Ruan YC, Pan Y, et al. Targeting GSTP1 as Therapeutic Strategy against Lung Adenocarcinoma Stemness and Resistance to Tyrosine Kinase Inhibitors. *Adv Sci*. (2023) 10(7). doi: 10.1002/advs.202205262

32. Xiao W.Loscalzo J. Metabolic Responses to Reductive Stress. *Antioxid Redox Signal*. (2020) 32(18):1330-47. doi: 10.1089/ars.2019.7803

33. Zhang W, Dai J, Hou G, Liu H, Zheng S, Wang X, et al. SMURF2 predisposes cancer cell toward ferroptosis in GPX4-independent manners by promoting GSTP1 degradation. *Mol Cell*. (2023) 83(23):4352-69.e8. doi: 10.1016/j.molcel.2023.10.042

34. Laborde E. Glutathione transferases as mediators of signaling pathways involved in cell proliferation and cell death. *Cell Death Differ*. (2010) 17(9):1373-80. doi: 10.1038/cdd.2010.80

35. Dimeloe S, Burgener AV, Grählert J.Hess C. T‐cell metabolism governing activation, proliferation and differentiation; a modular view. *Immunology*. (2016) 150(1):35-44. doi: 10.1111/imm.12655

36. Wang H, Jiang D, Liu L, Zhang Y, Qin M, Qu Y, et al. Spermidine Promotes Nb CAR-T Mediated Cytotoxicity to Lymphoma Cells Through Elevating Proliferation and Memory. *OncoTargets Ther*. (2022) Volume 15:1229-43. doi: 10.2147/ott.S382540

37. Thongchot S, Aksonnam K, Prasopsiri J, Warnnissorn M, Sa-nguanraksa D, O-Charoenrat P, et al. Mesothelin- and nucleolin-specific T cells from combined short peptides effectively kill triple-negative breast cancer cells. *BMC Medicine*. (2024) 22(1). doi: 10.1186/s12916-024-03625-3

38. van Bruggen JAC, Martens AWJ, Fraietta JA, Hofland T, Tonino SH, Eldering E, et al. Chronic lymphocytic leukemia cells impair mitochondrial fitness in CD8+ T cells and impede CAR T-cell efficacy. *Blood*. (2019) 134(1):44-58. doi: 10.1182/blood.2018885863

39. Rotte A, Frigault MJ, Ansari A, Gliner B, Heery C.Shah B. Dose–response correlation for CAR-T cells: a systematic review of clinical studies. *J Immunother Cancer*. (2022) 10(12). doi: 10.1136/jitc-2022-005678

40. Tang L, Pan S, Wei X, Xu X.Wei Q. Arming CAR-T cells with cytokines and more: Innovations in the fourth-generation CAR-T development. *Mol Ther*. (2023) 31(11):3146-62. doi: 10.1016/j.ymthe.2023.09.021

41. Seo W, Jerin C.Nishikawa H. Transcriptional regulatory network for the establishment of CD8+ T cell exhaustion. *Exp Mol Med*. (2021) 53(2):202-09. doi: 10.1038/s12276-021-00568-0
