# Supplementary figures and images for: GSTP1 improves CAR-T cell proliferation and cytotoxicity to combat lymphoma
Source: Front Immunol. 2025 Sep 26;16:1665407. doi: 10.3389/fimmu.2025.1665407 (PMC12511145; doi:10.3389/fimmu.2025.1665407)

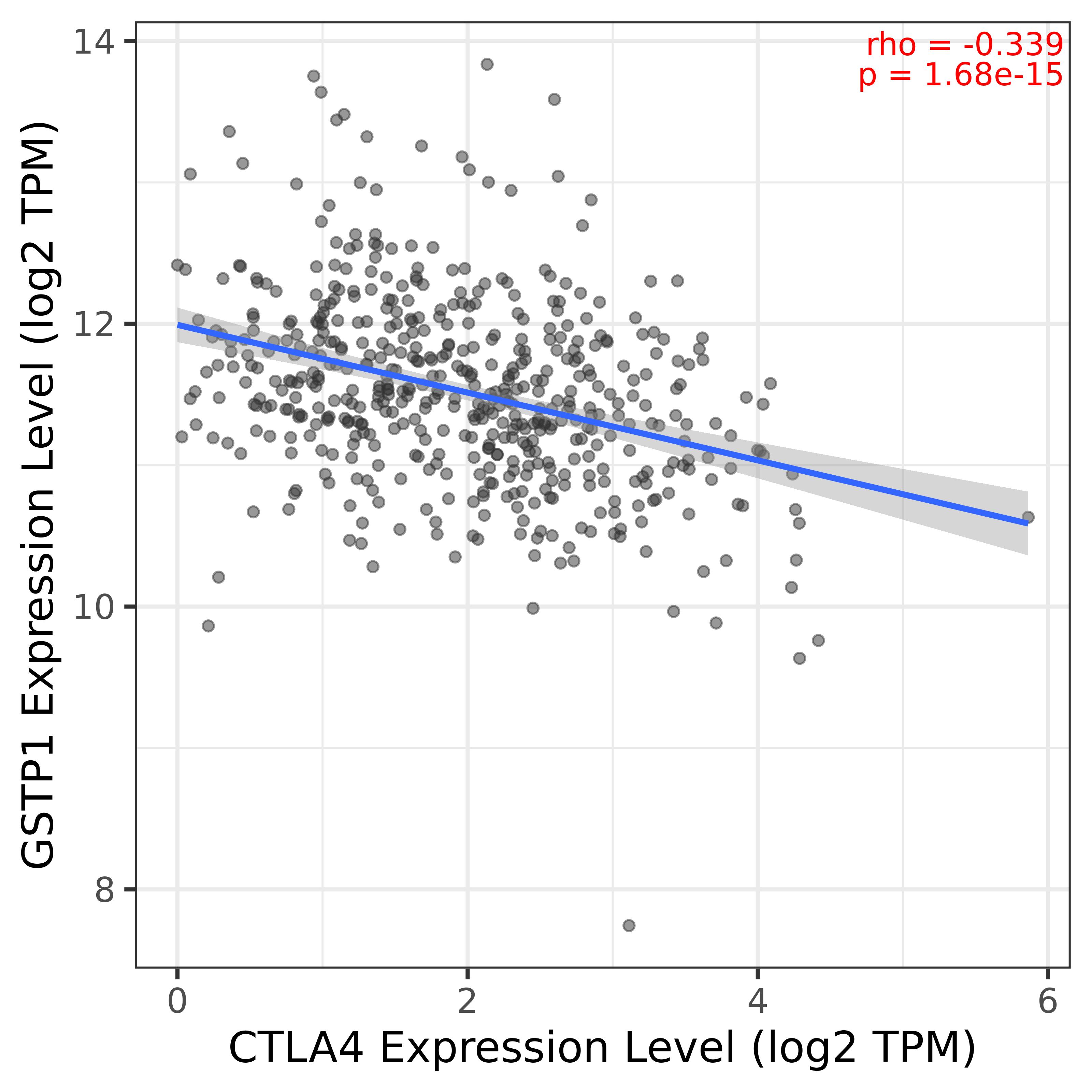

Supplement: Supplementary file 1 [file DataSheet1.zip › Raw date/Figure1/A/genecorr_plot (1).jpg]

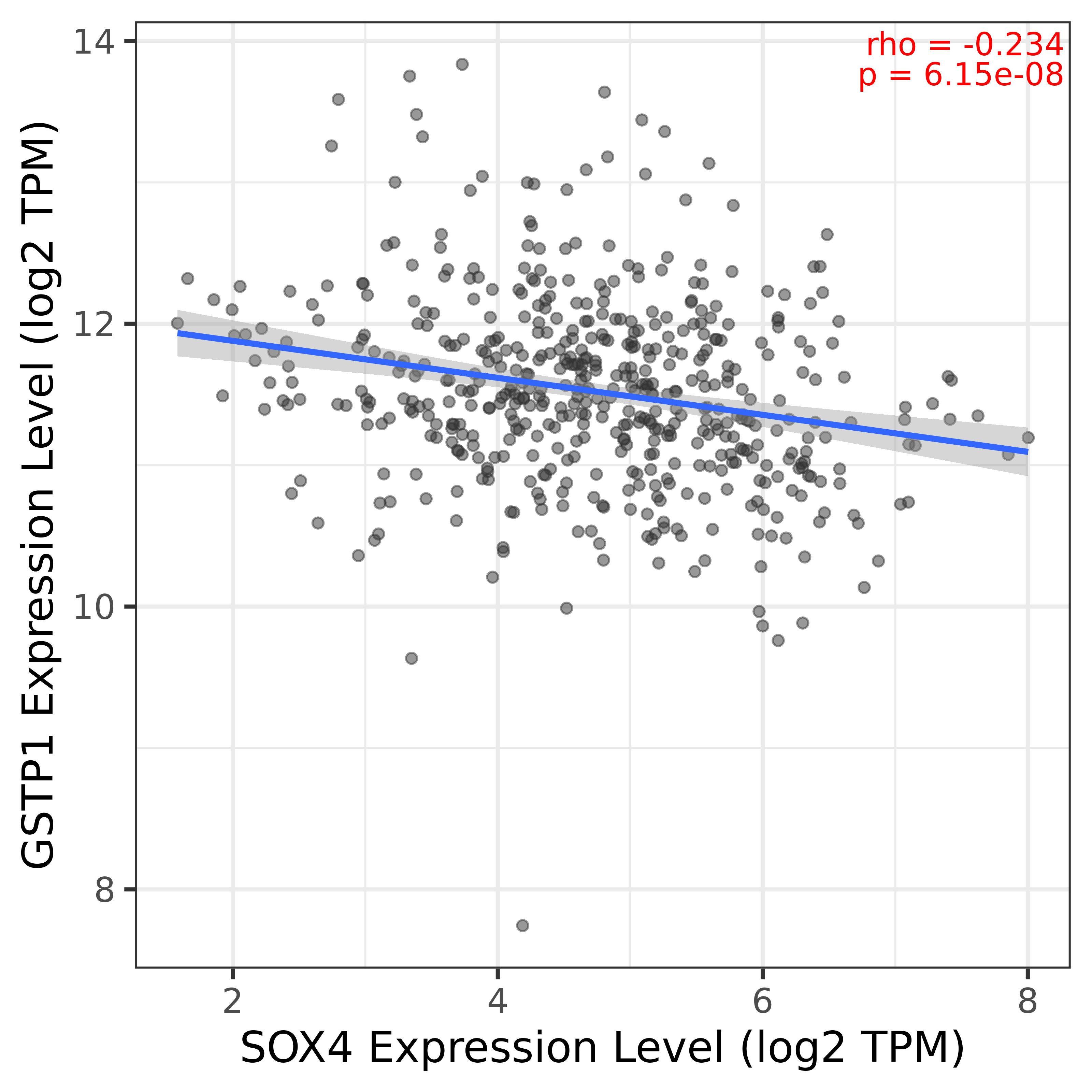

Supplement: Supplementary file 1 [file DataSheet1.zip › Raw date/Figure1/A/genecorr_plot (10).jpg]

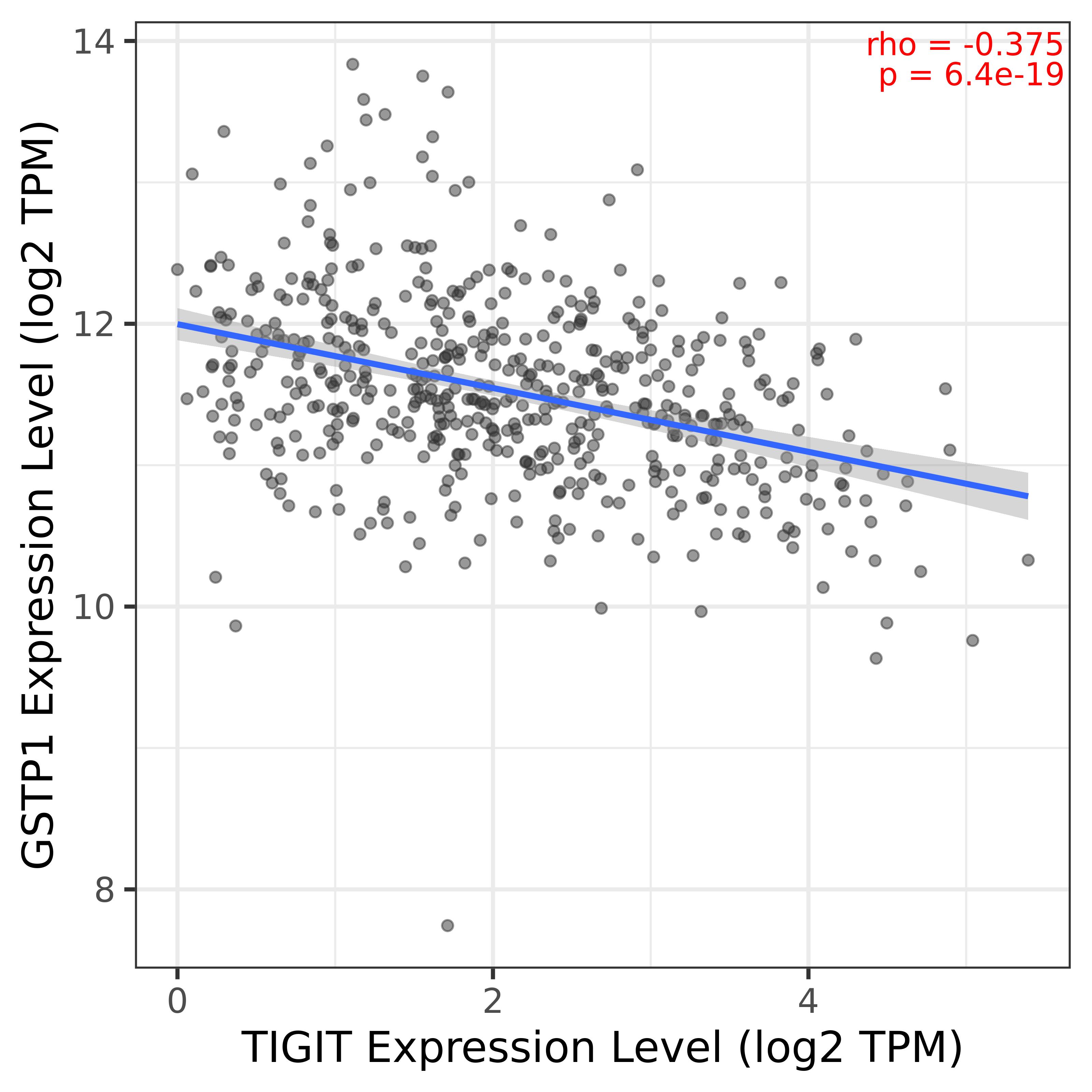

Supplement: Supplementary file 1 [file DataSheet1.zip › Raw date/Figure1/A/genecorr_plot (11).jpg]

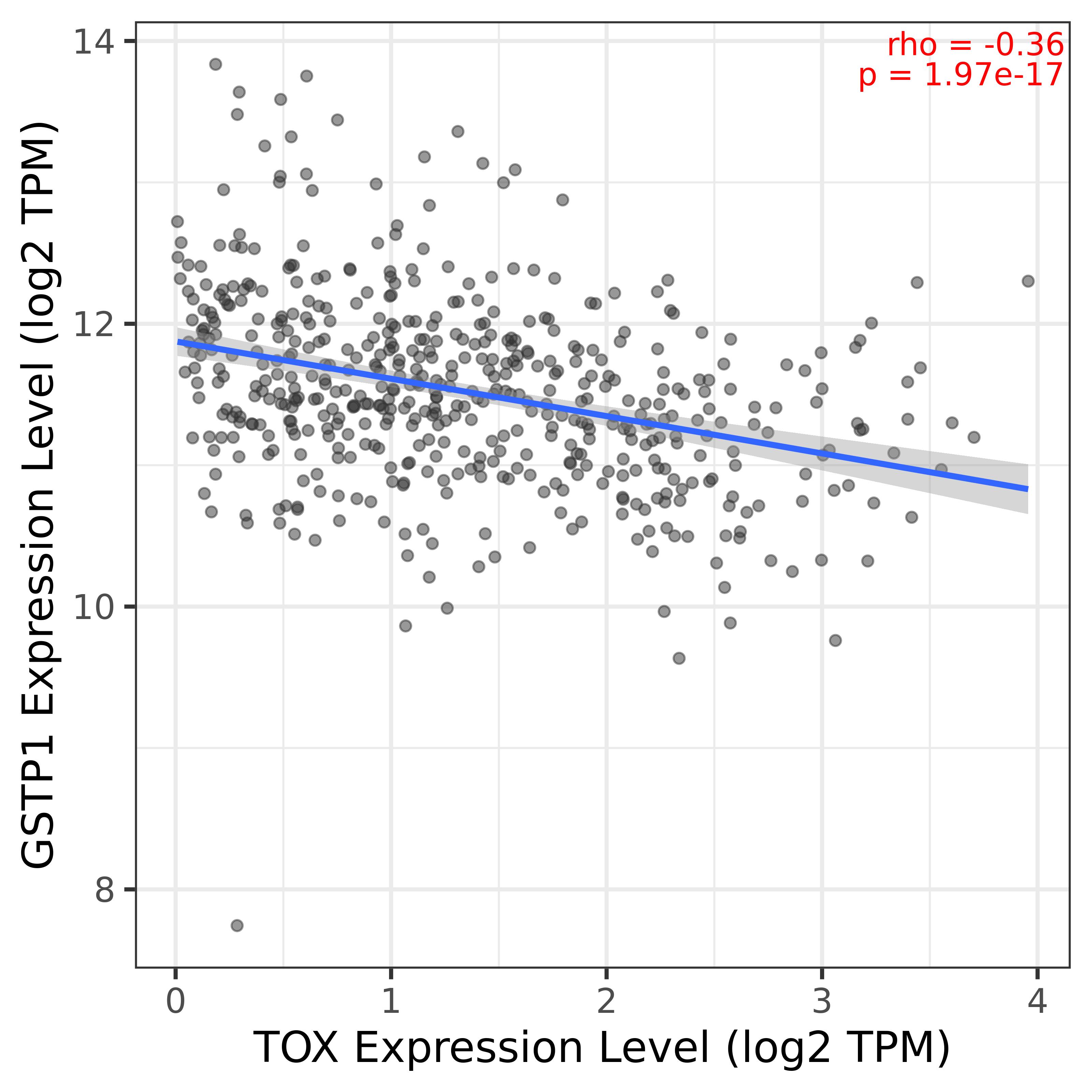

Supplement: Supplementary file 1 [file DataSheet1.zip › Raw date/Figure1/A/genecorr_plot (12).jpg]

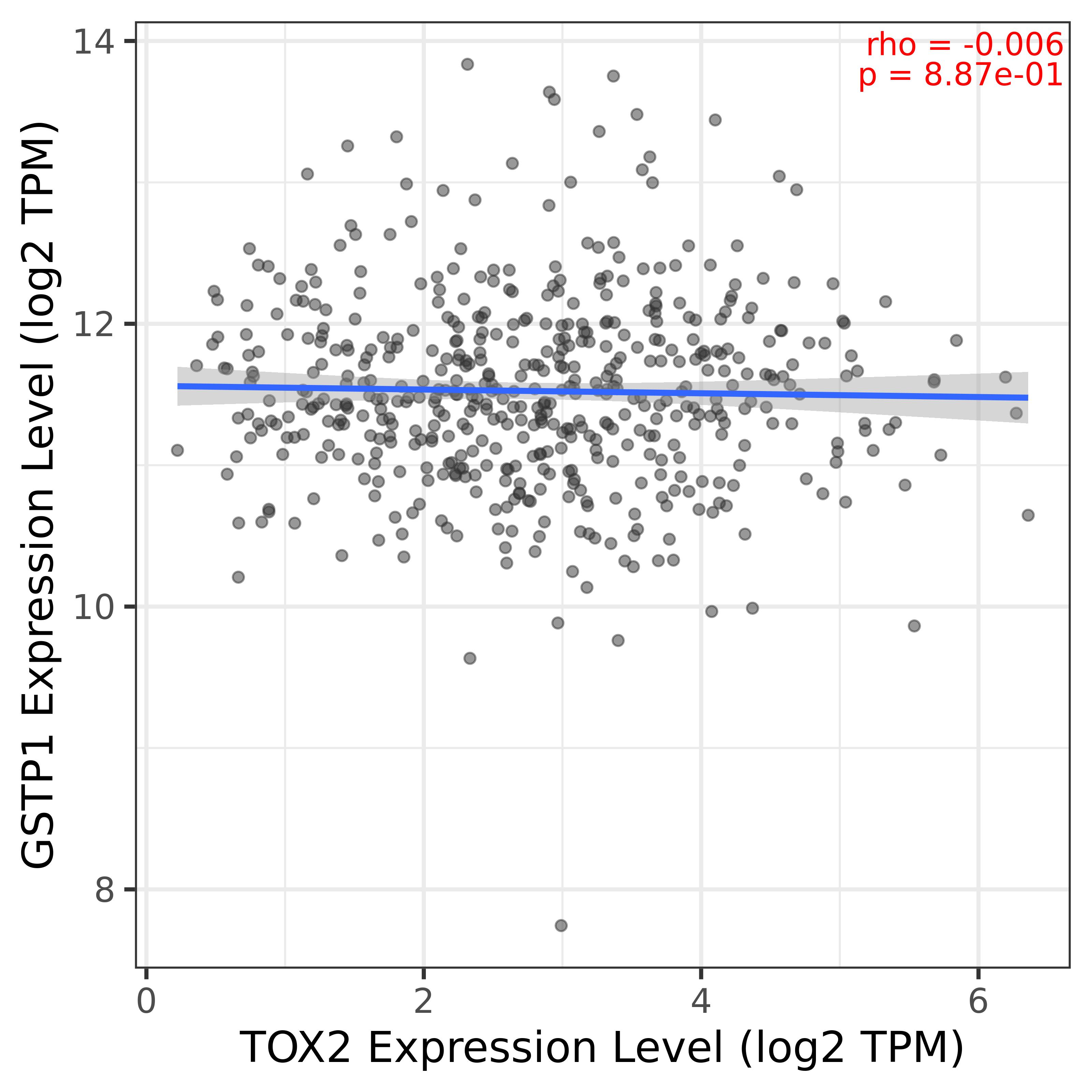

Supplement: Supplementary file 1 [file DataSheet1.zip › Raw date/Figure1/A/genecorr_plot (13).jpg]

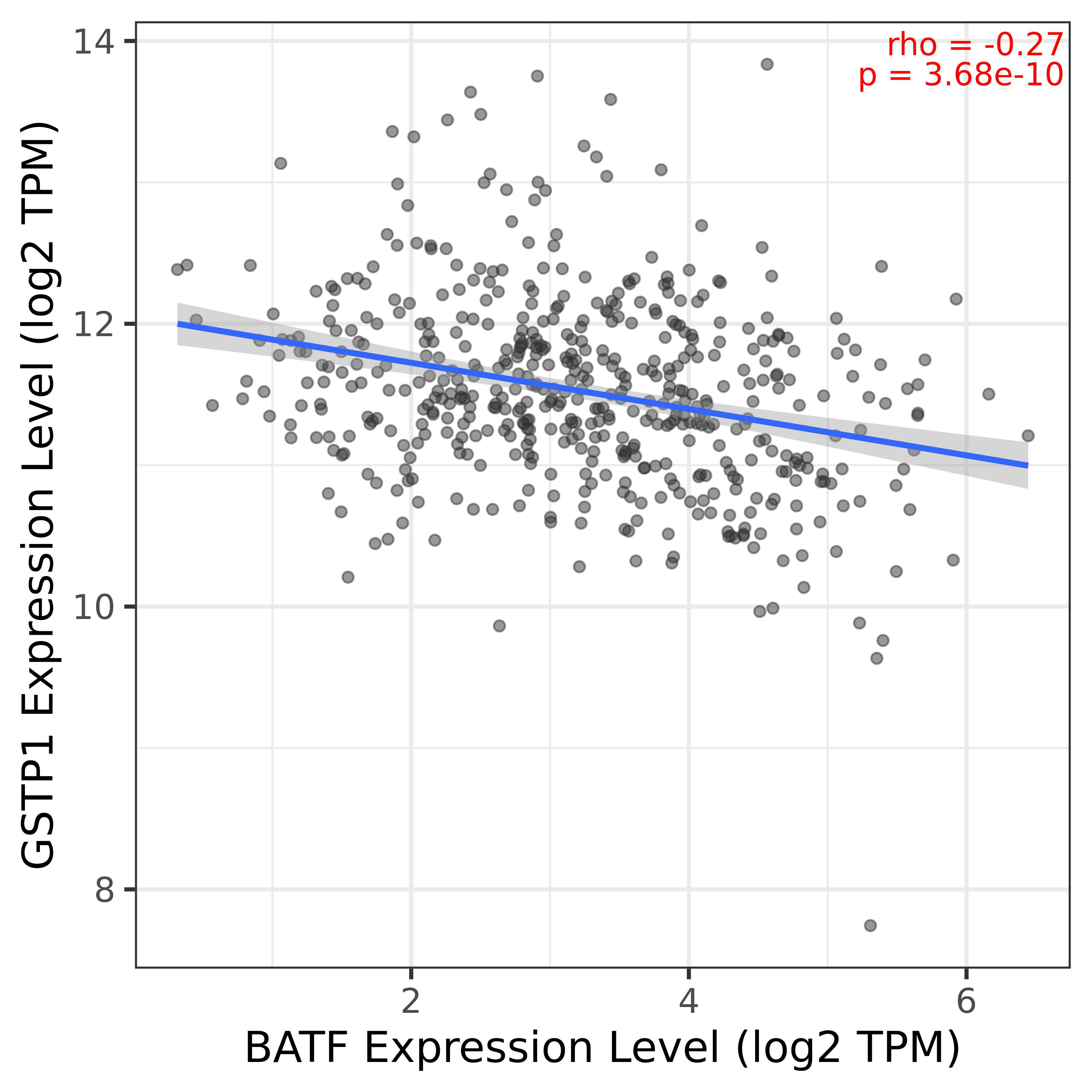

Supplement: Supplementary file 1 [file DataSheet1.zip › Raw date/Figure1/A/genecorr_plot (14).jpg]

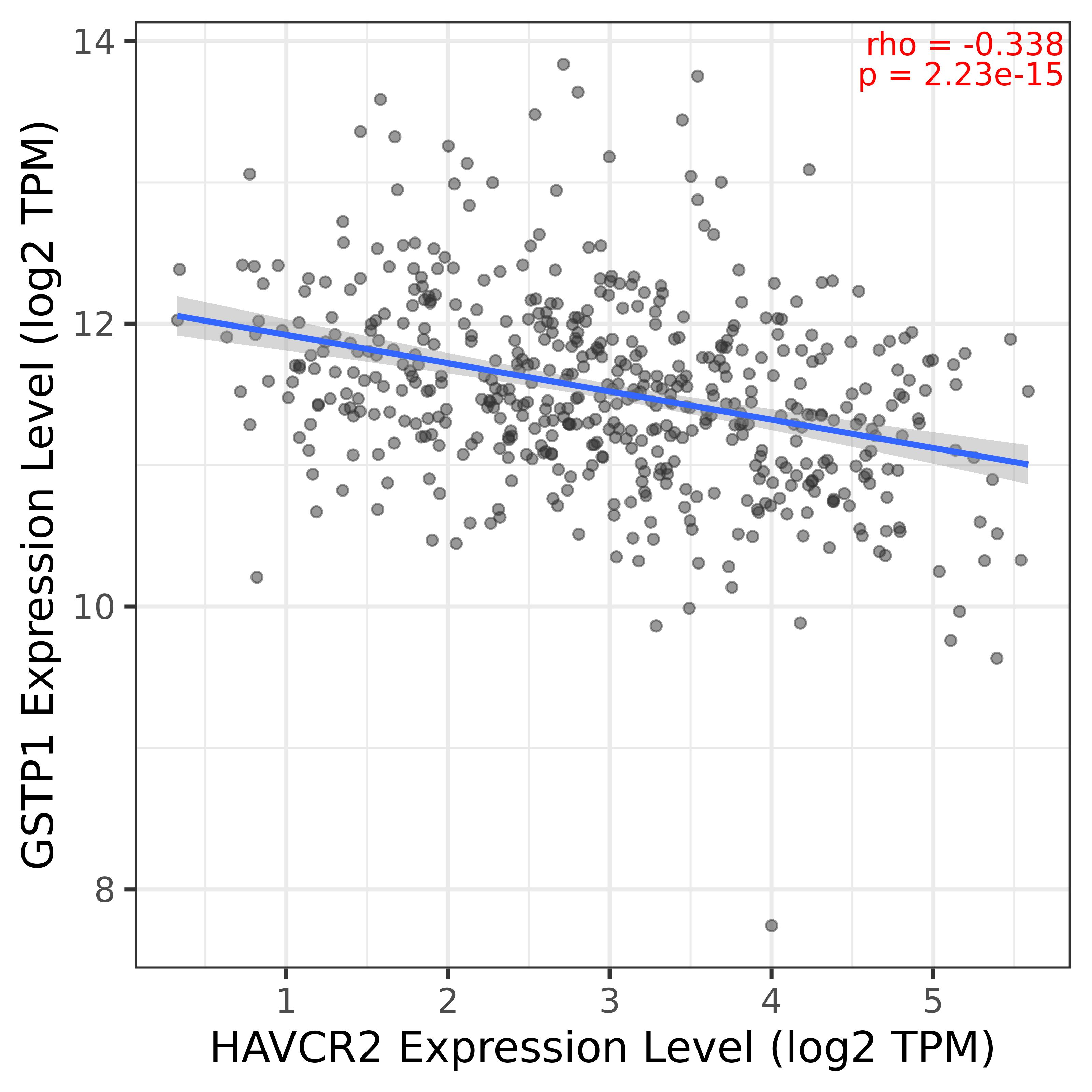

Supplement: Supplementary file 1 [file DataSheet1.zip › Raw date/Figure1/A/genecorr_plot (2).jpg]

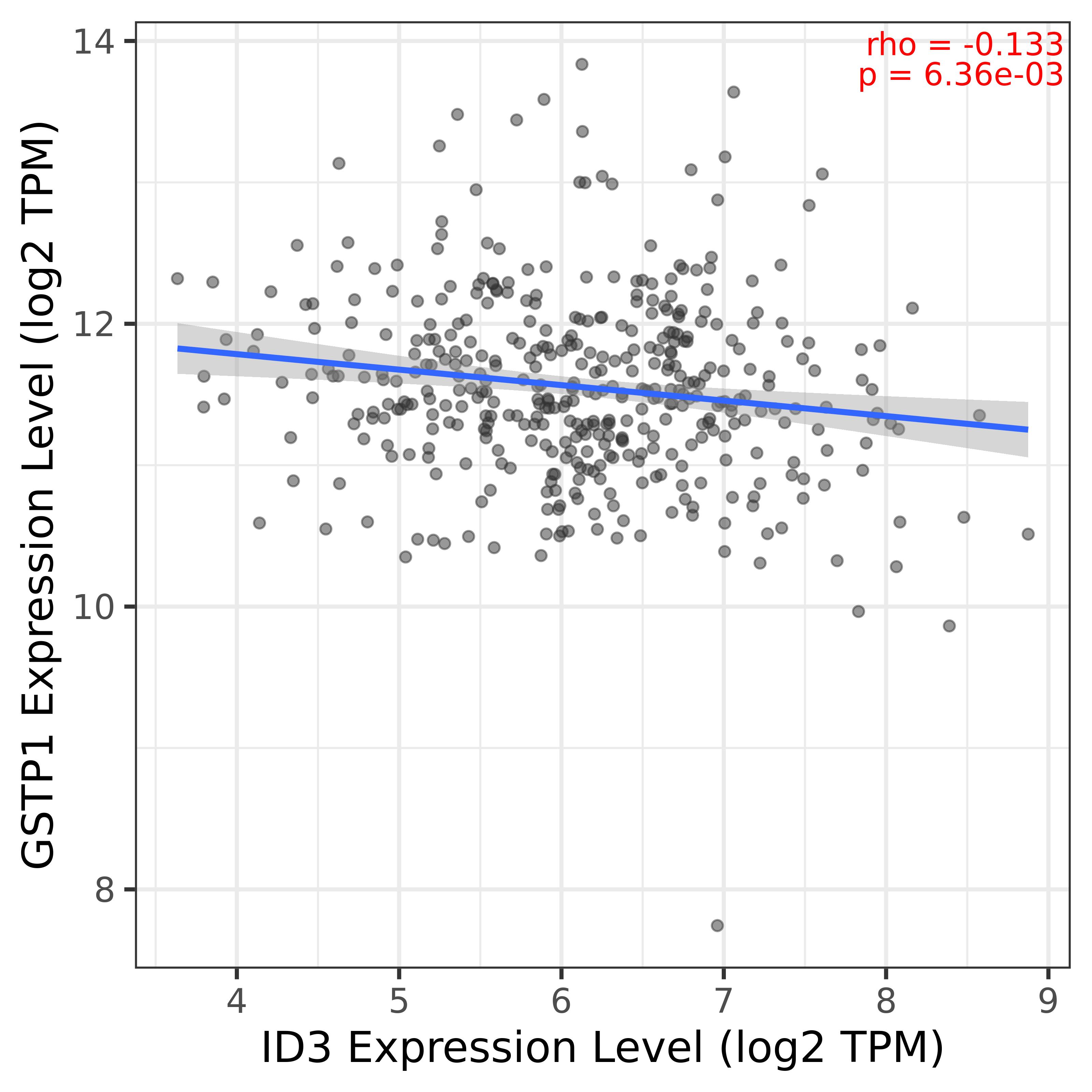

Supplement: Supplementary file 1 [file DataSheet1.zip › Raw date/Figure1/A/genecorr_plot (3).jpg]

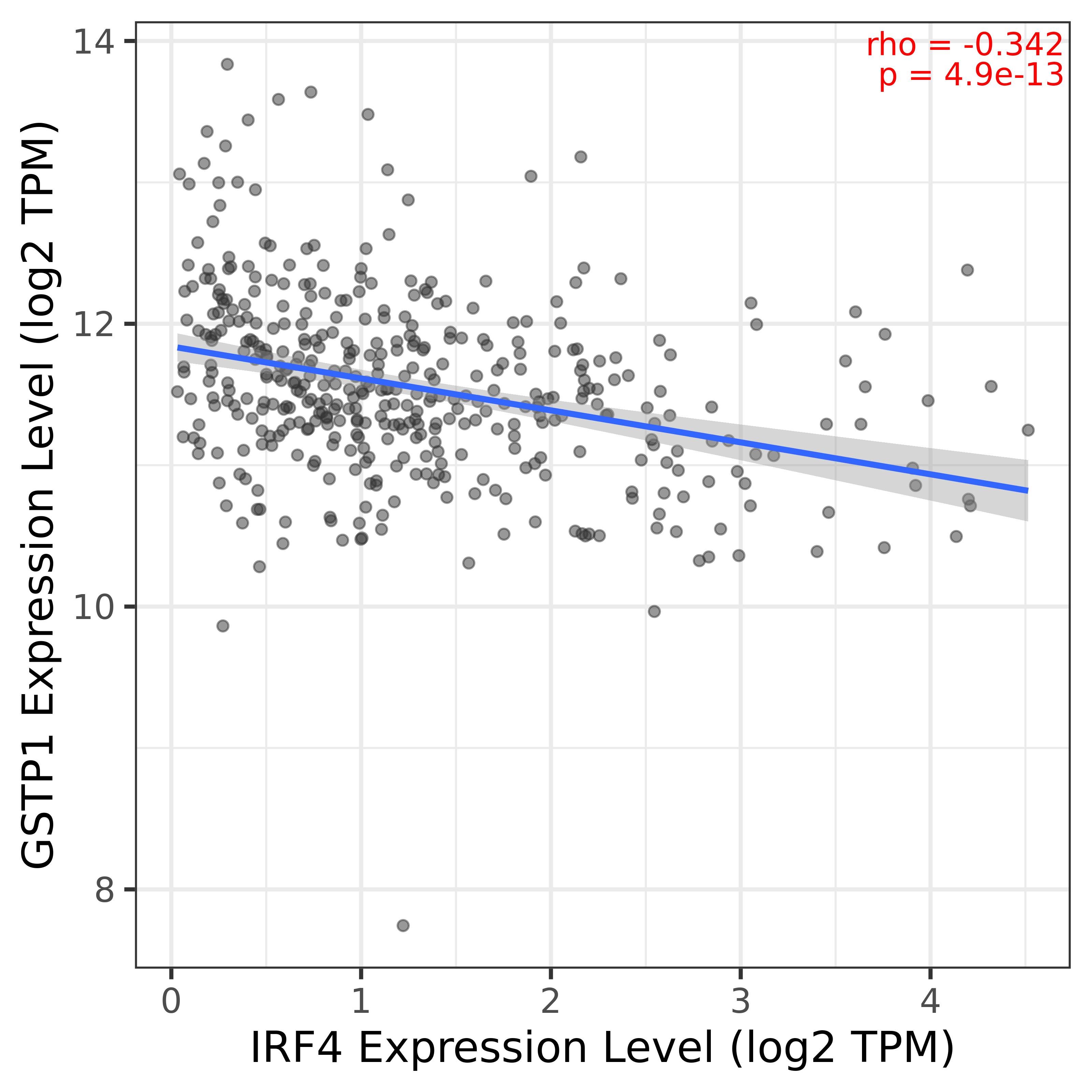

Supplement: Supplementary file 1 [file DataSheet1.zip › Raw date/Figure1/A/genecorr_plot (4).jpg]

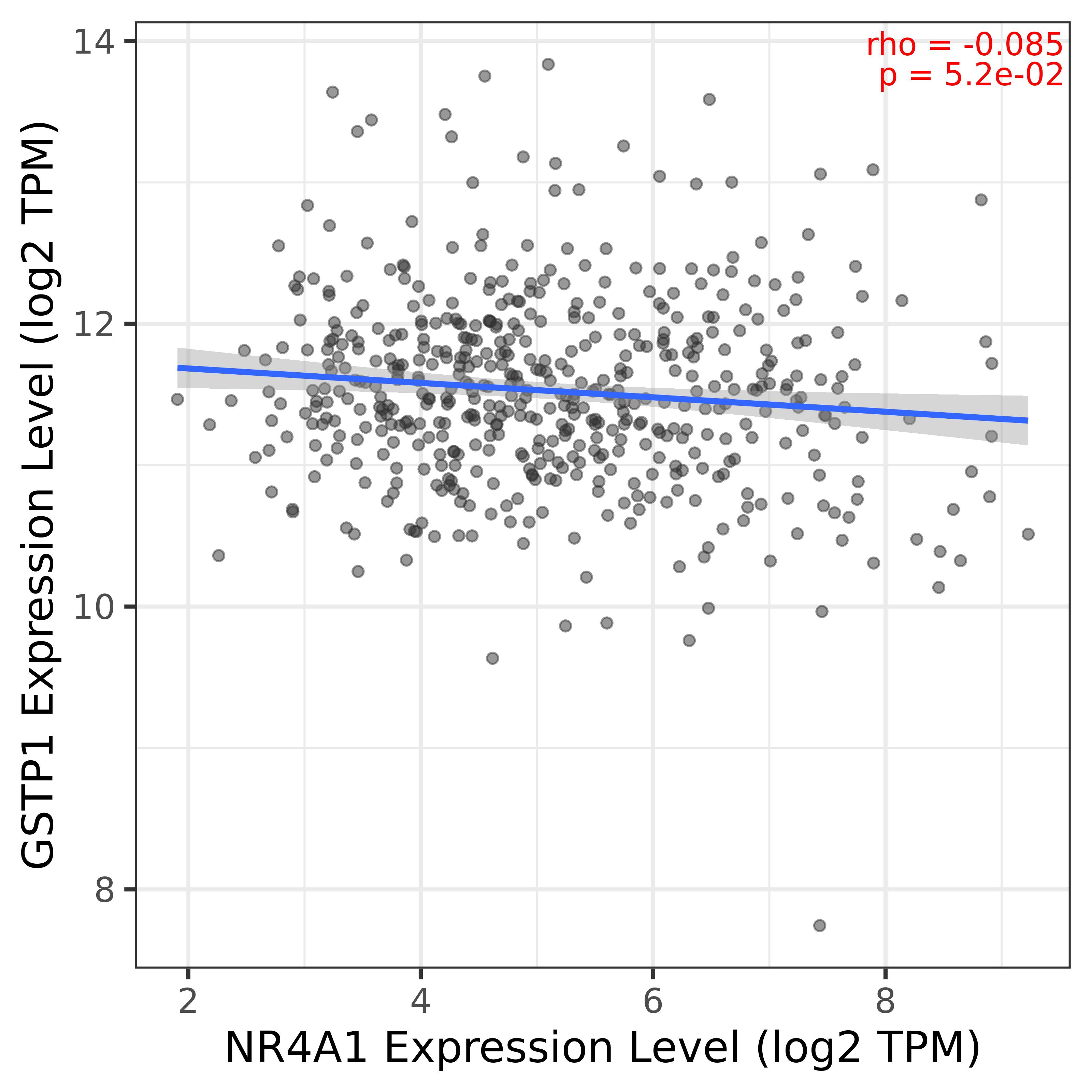

Supplement: Supplementary file 1 [file DataSheet1.zip › Raw date/Figure1/A/genecorr_plot (5).jpg]

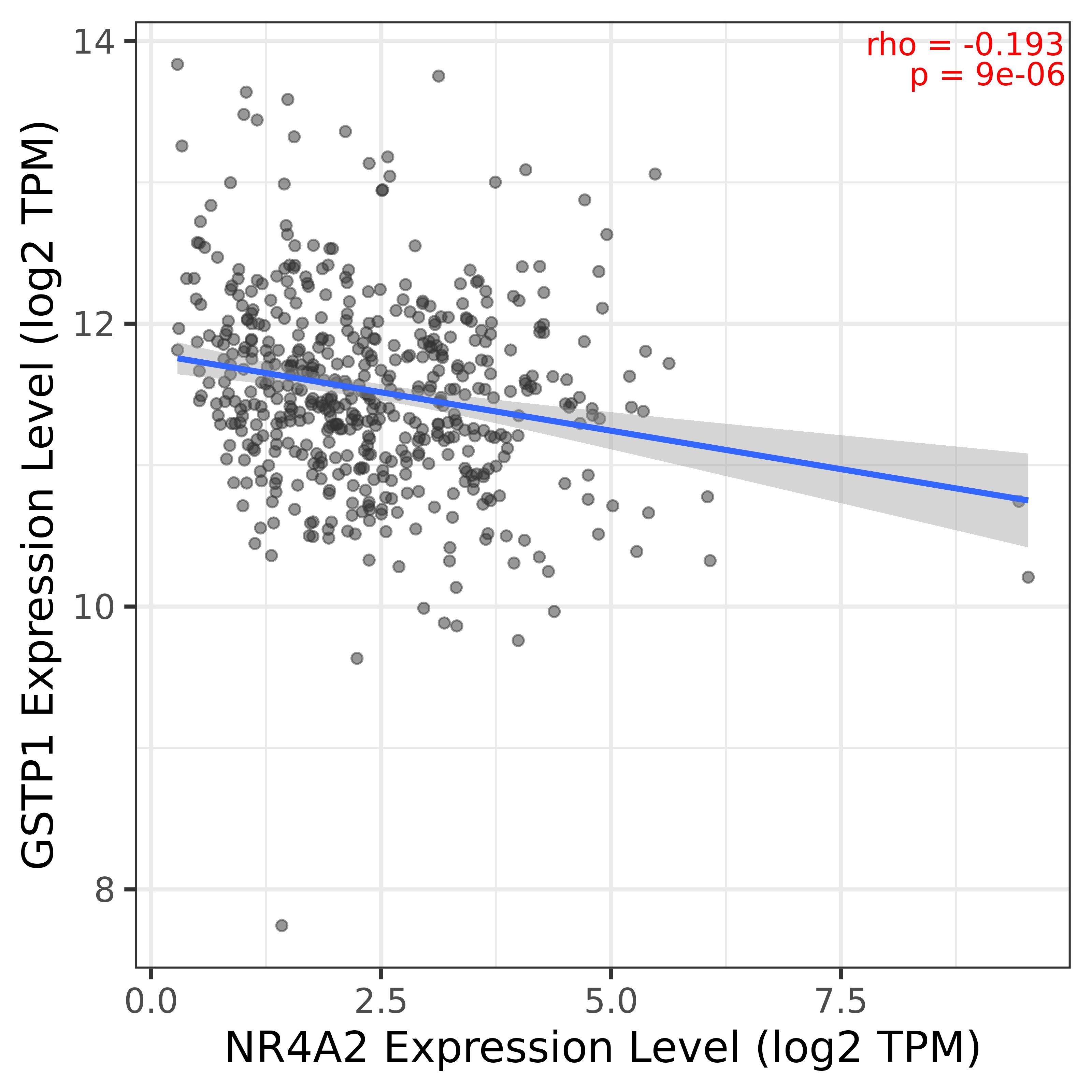

Supplement: Supplementary file 1 [file DataSheet1.zip › Raw date/Figure1/A/genecorr_plot (6).jpg]

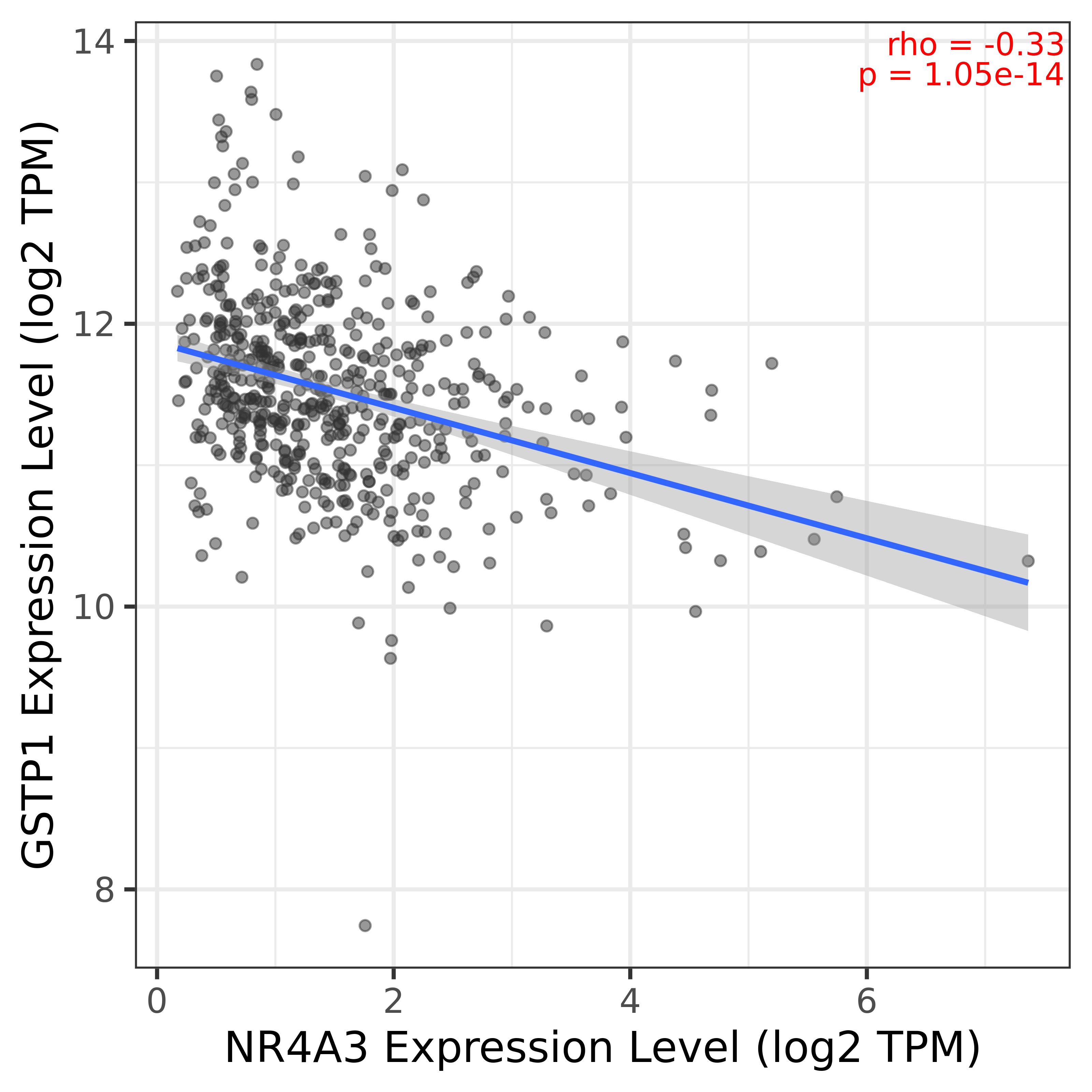

Supplement: Supplementary file 1 [file DataSheet1.zip › Raw date/Figure1/A/genecorr_plot (7).jpg]

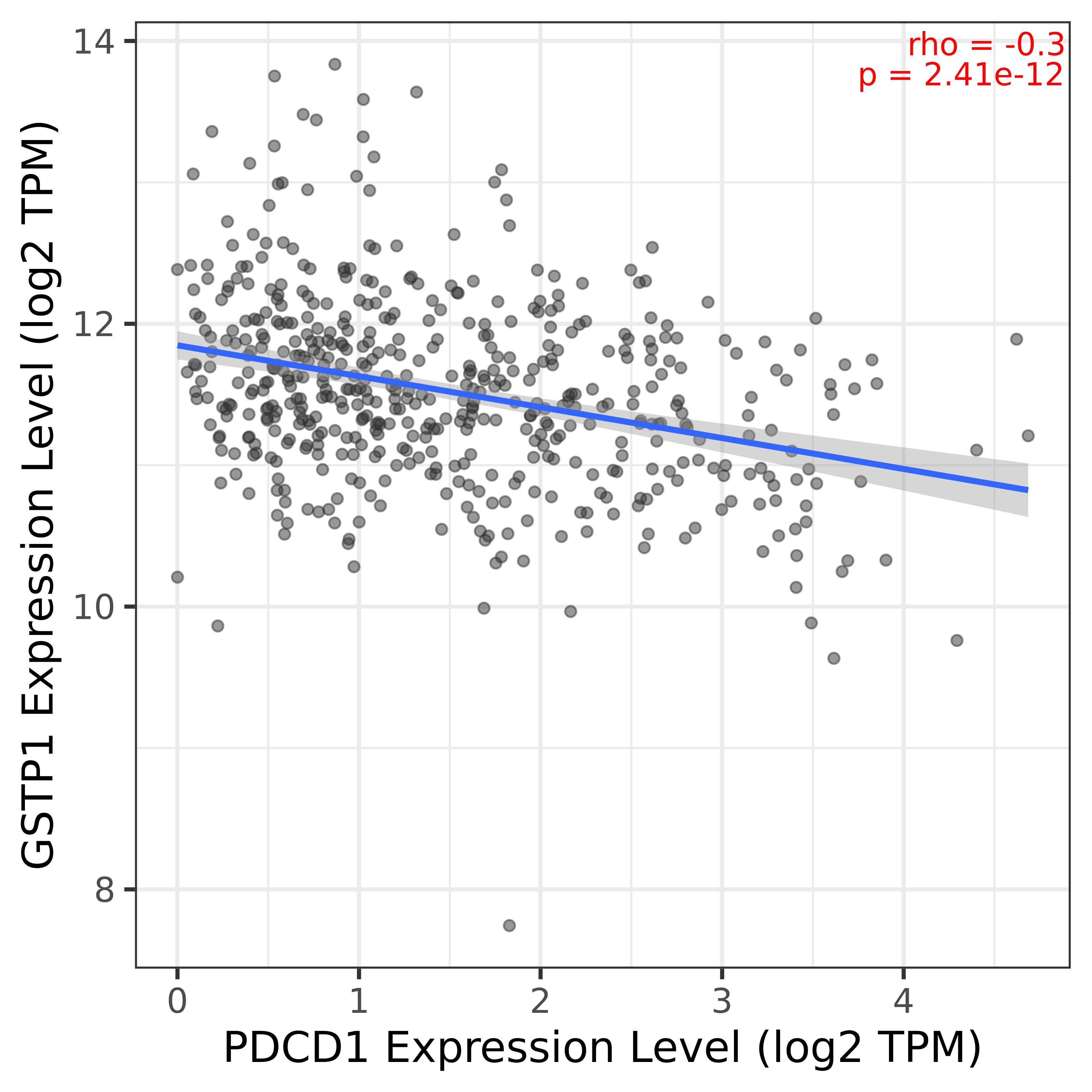

Supplement: Supplementary file 1 [file DataSheet1.zip › Raw date/Figure1/A/genecorr_plot (8).jpg]

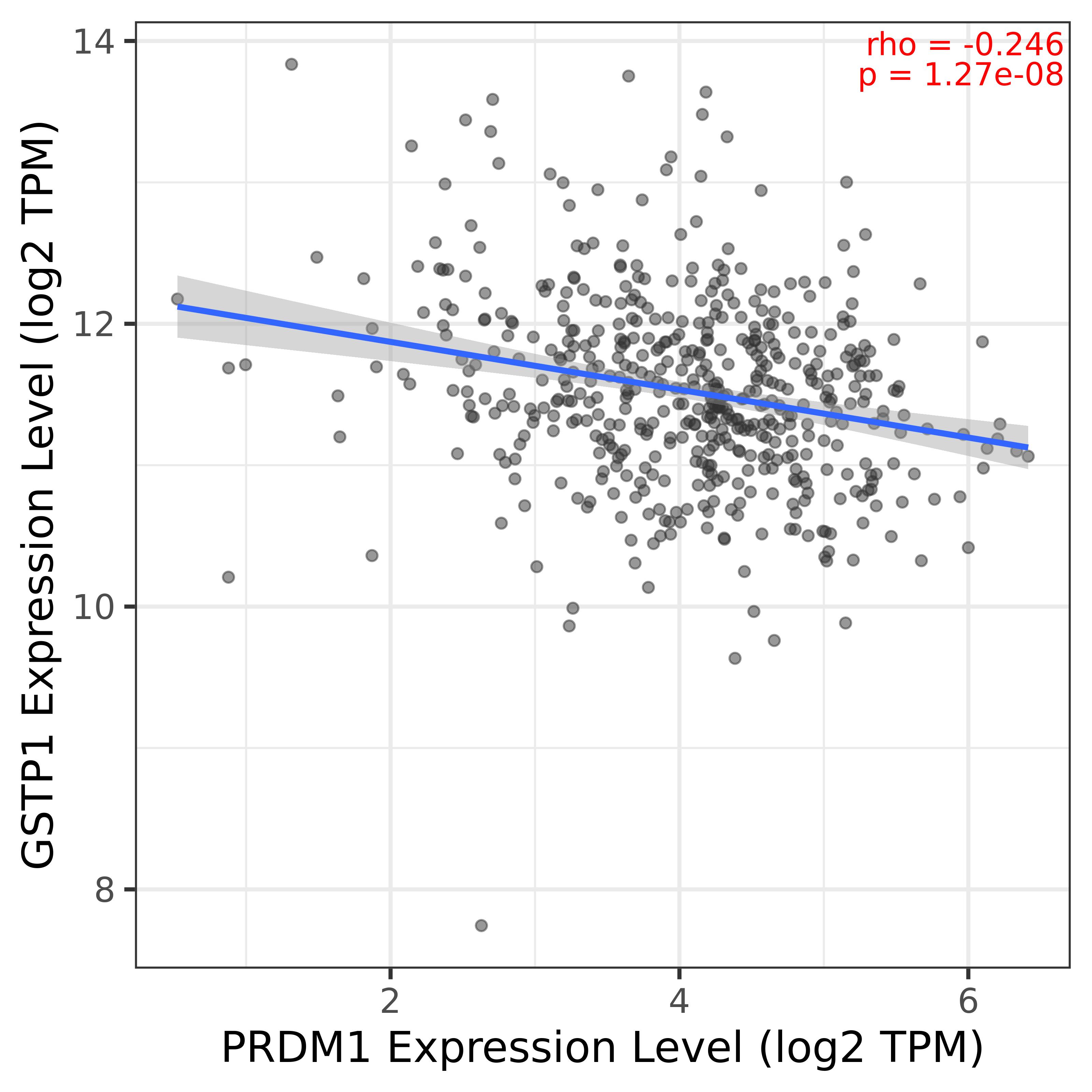

Supplement: Supplementary file 1 [file DataSheet1.zip › Raw date/Figure1/A/genecorr_plot (9).jpg]

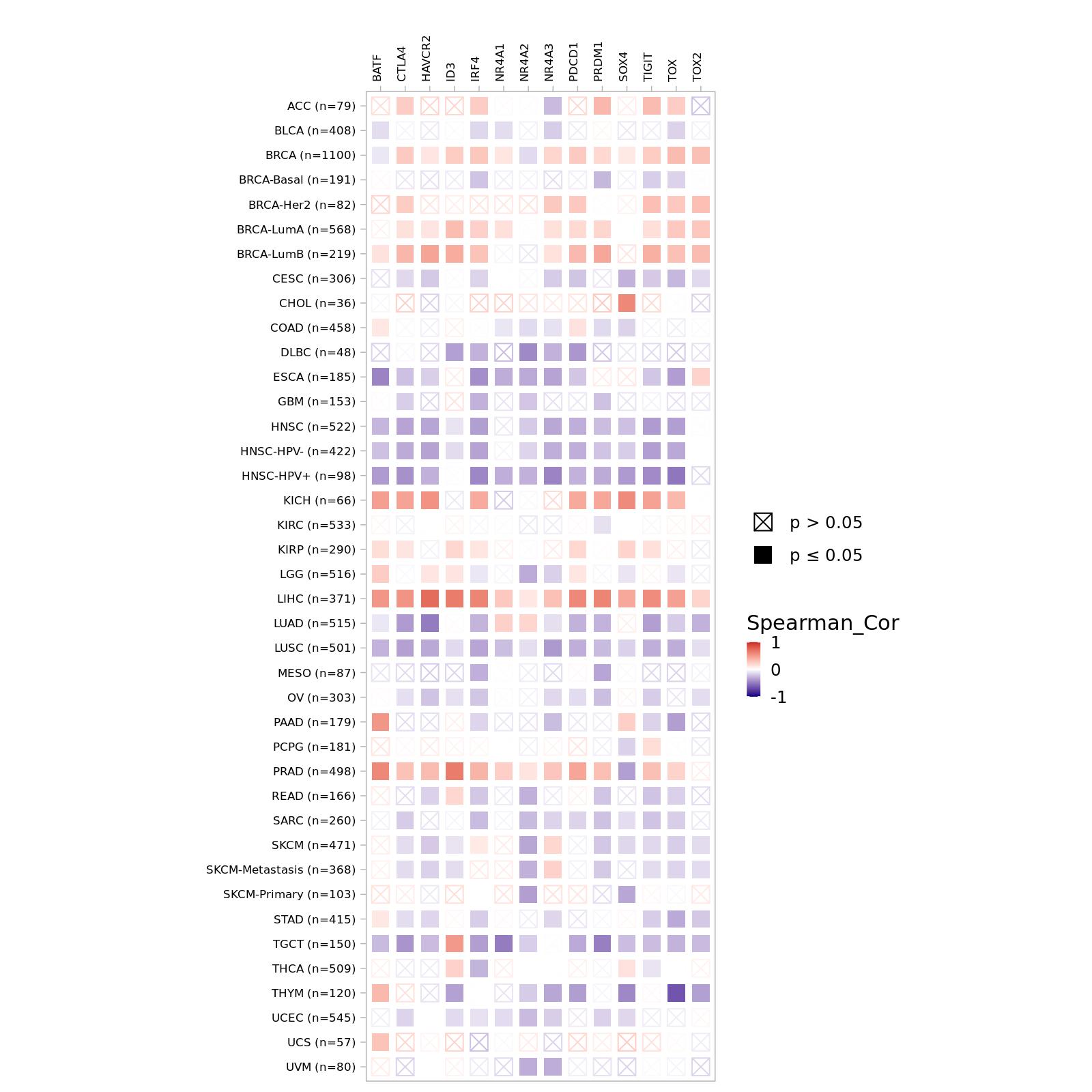

Supplement: Supplementary file 1 [file DataSheet1.zip › Raw date/Figure1/A/genecorr_table (2).jpg]

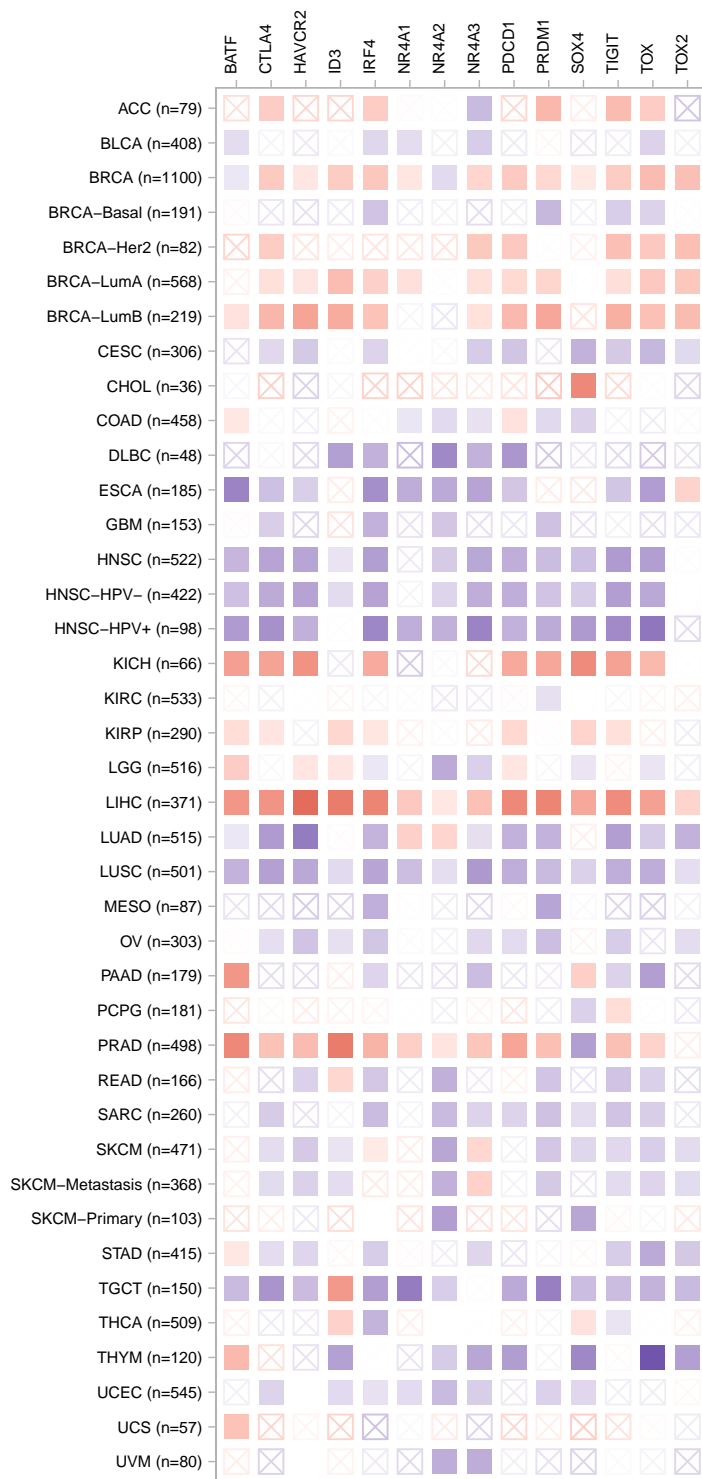

☐ p > 0.05

■ p ... 0.05

Spearman\_Cor

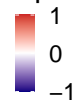

Supplement: Supplementary file 1 [file DataSheet1.zip › Raw date/Figure1/A/genecorr_table (2).pdf]

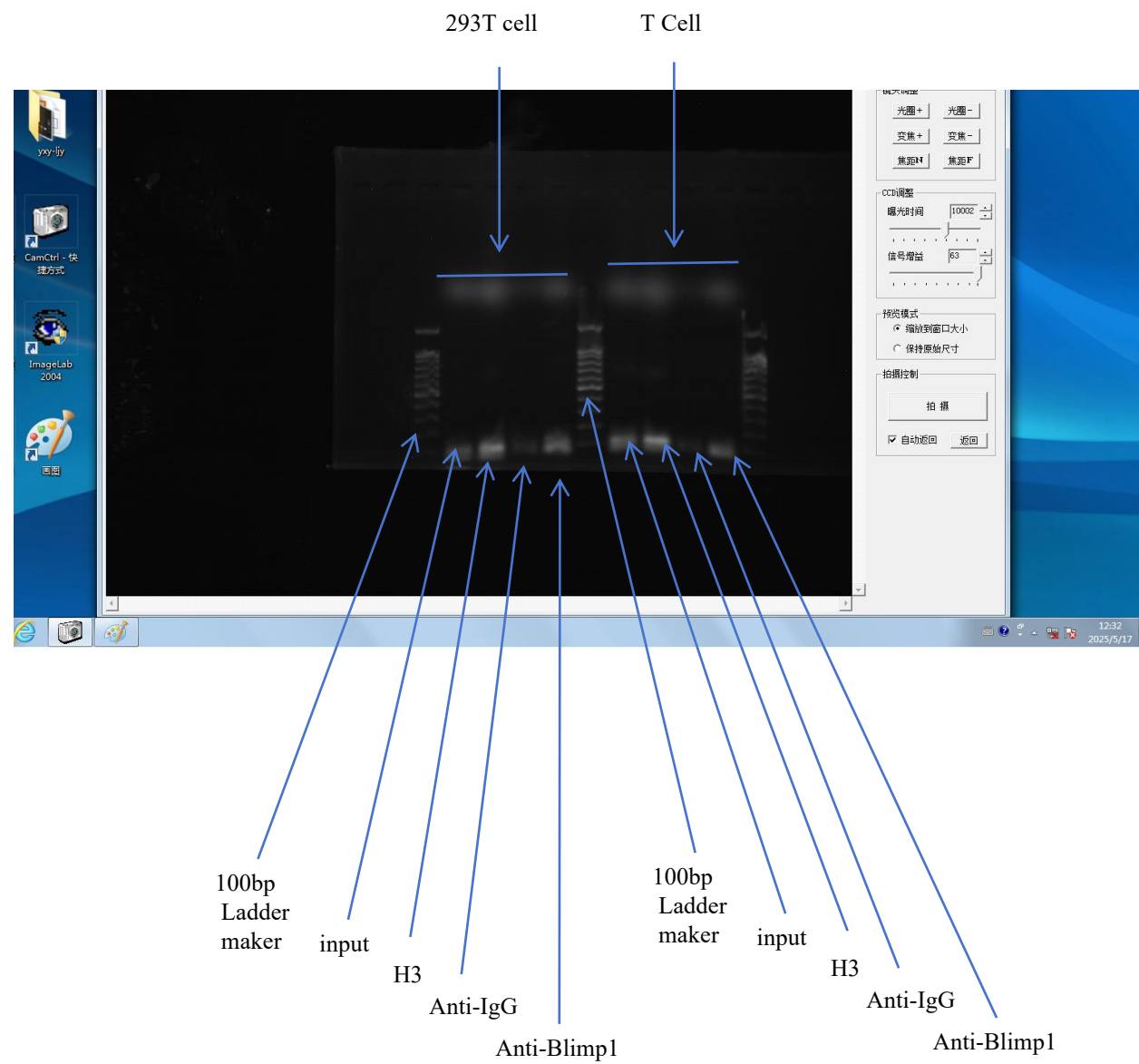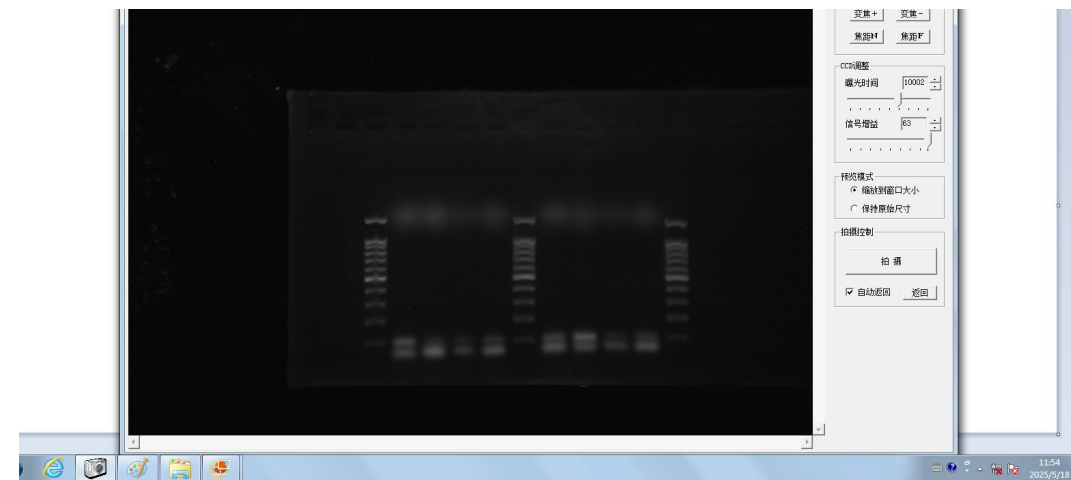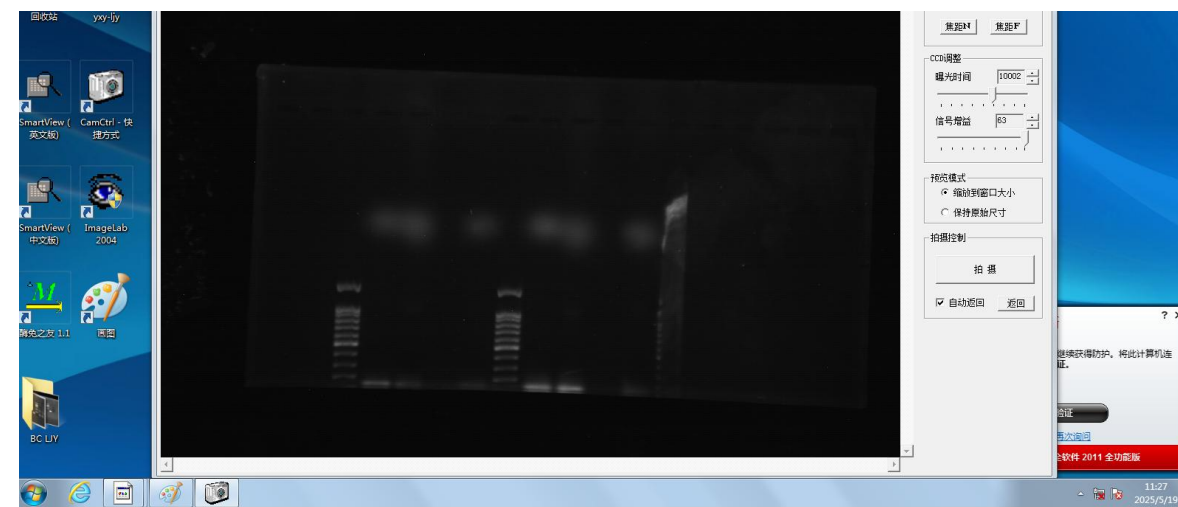

Supplement: Supplementary file 1 [file DataSheet1.zip › Raw date/Figure2/H/Raw date of ChIP-qPCR.pdf]

GAPDH

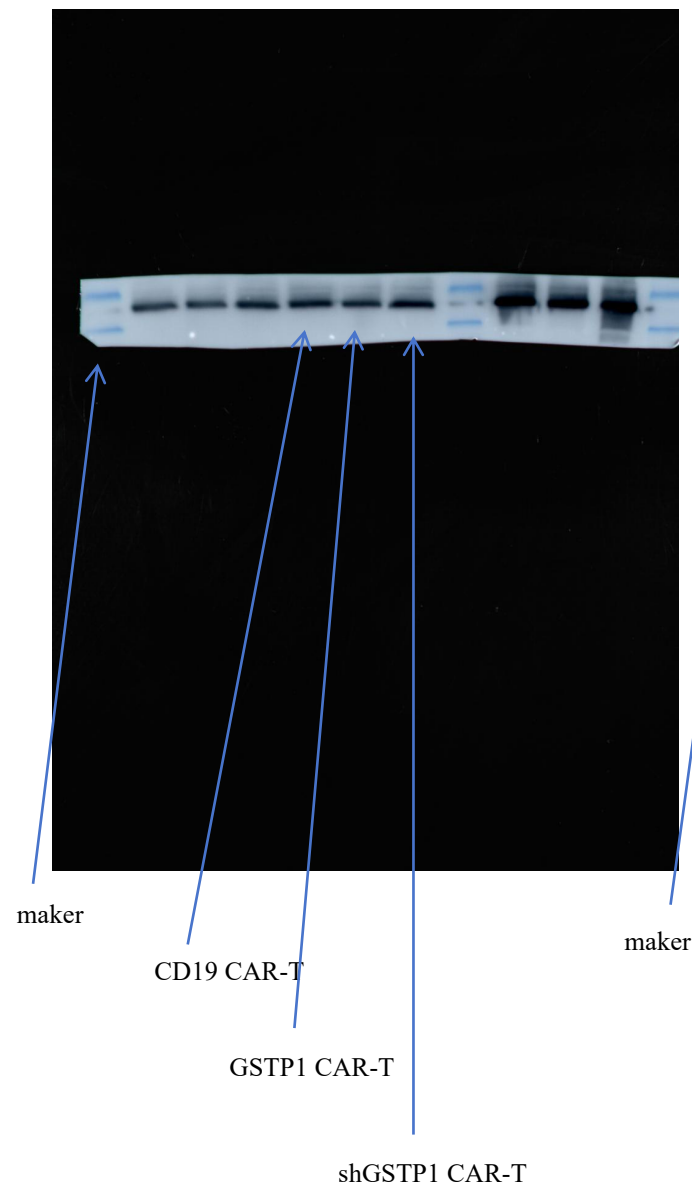

GSTP1

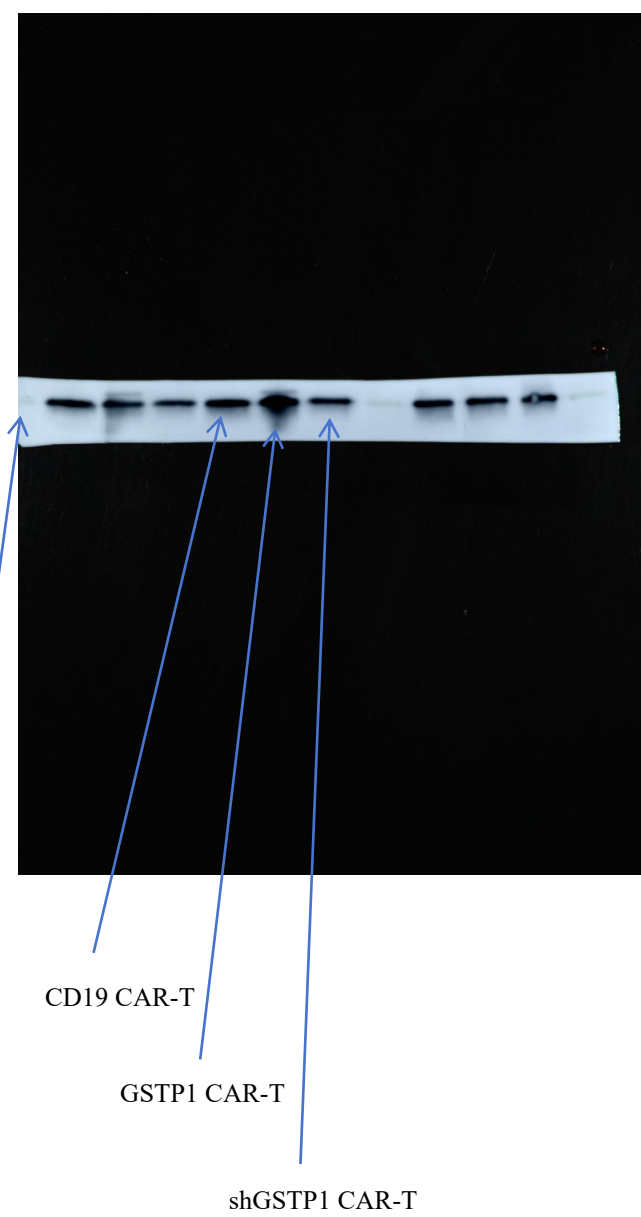

Supplement: Supplementary file 1 [file DataSheet1.zip › Raw date/Figure3/E/Validation of GSTP1 CAR-T and shGSTP1 CAR-Tú¿WB).pdf]
